# Supplementary material for: A living ex vivo platform for functional, personalized brain cancer diagnosis
Source: Cell Rep Med. 2023 May 15;4(6):101042. doi: 10.1016/j.xcrm.2023.101042 (PMC10313921; doi:10.1016/j.xcrm.2023.101042)
Supplement: Document S1. Figures S1–S7 and Tables S1–S4 [file mmc1.pdf]

Cell Reports Medicine, Volume *4*

Supplemental information

A living ex vivo platform for functional, personalized brain cancer diagnosis

Breanna Mann, Xiaopei Zhang, Noah Bell, Adebimpe Adefolaju, Morrent Thang, Rajaneekar Dasari, Krishna Kanchi, Alain Valdivia, Yang Yang, Andrew Buckley, Vivien Lettry, Carolyn Quinsey, Yasmeen Rauf, David Kram, Noah Cassidy, Cyrus Vaziri, David L. Corcoran, Stephen Rego, Yuchao Jiang, Lee M. Graves, Denise Dunn, Scott Floyd, Albert Baldwin, Shawn Hingtgen, and Andrew B. Satterlee

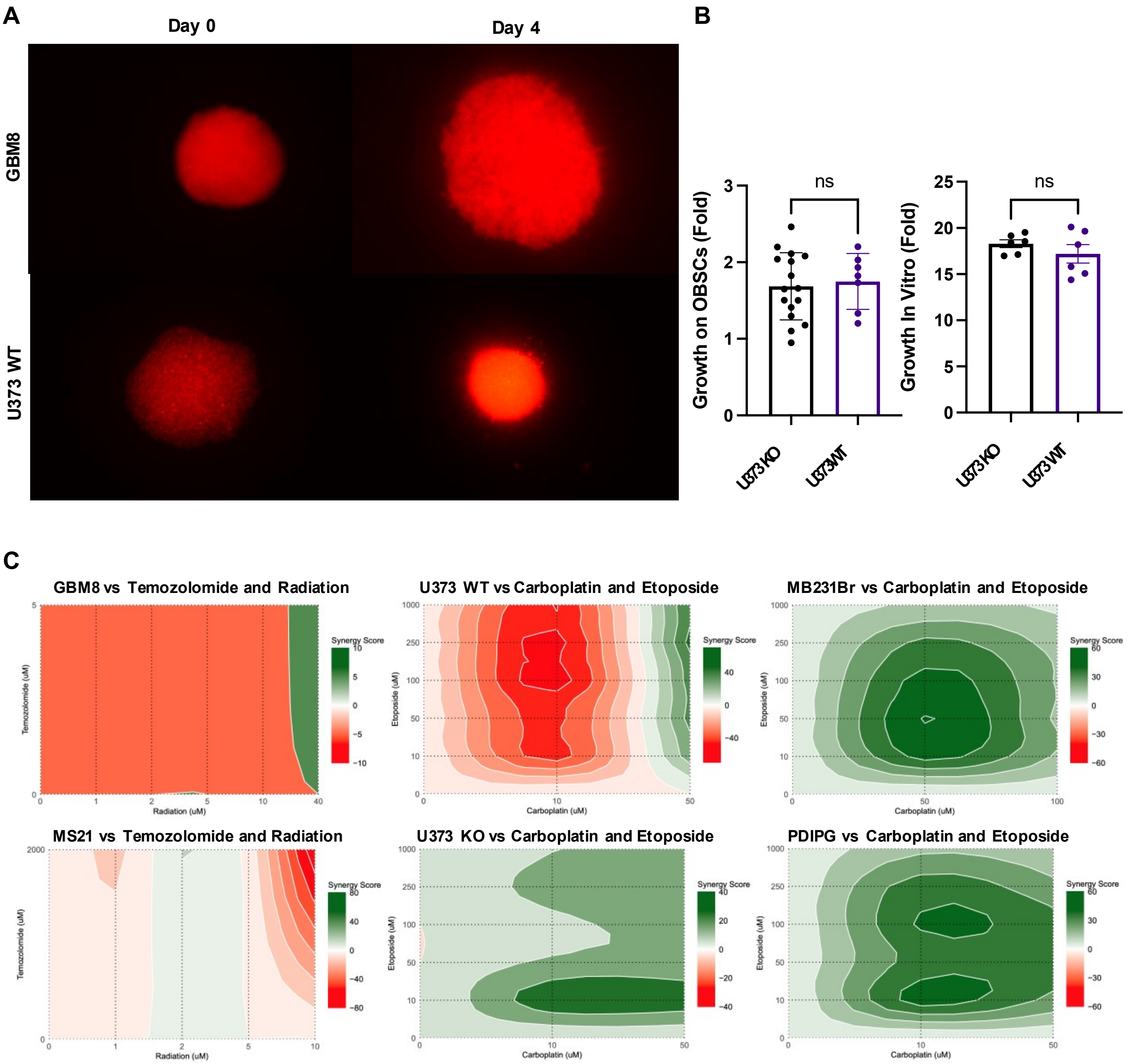

Figure S1, Related to Figures 2 and 3: Additional Tumor Growth and Synergy Plots. A) Fluorescence images of GBM8 and U373 WT tumor foci on day 0 and day 4. B) Comparison of U373WT and U373KO growth on OBSCs and *in vitro*. (n□6 technical replicates per group). Analyzed using an unpaired t-test. C) ZIP Synergy Score plots representing synergistic or antagonistic effects of combination therapies displayed in Fig 3C.

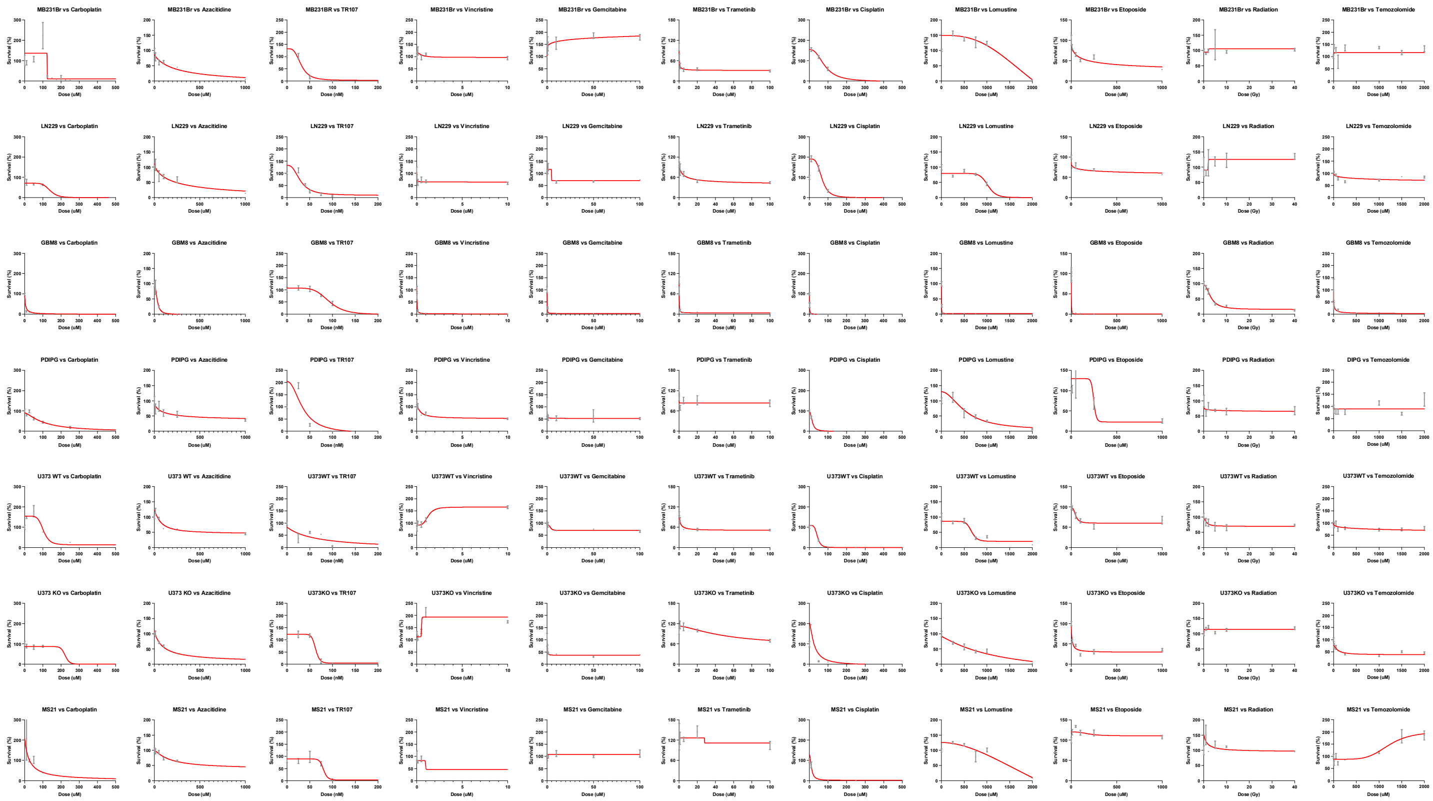

Figure S2, Related to Figure 3 and 4: Killing Curves on OBSCs. Dose-response curves of each cell line against each therapeutic. To enable qualitative comparison, all datasets are fit with an inhibitor vs response variable slope (four parameters) least squares fit. This method poorly fits some curve types, such as those with increases in growth at low concentrations. Our IC50 and DSS calculations do not use these fitted curves, but instead use linear interpolation between calculated data points.

	MB231BR										
	Carboplatin	Azacitidine	Temozolomide	TR107	Vincristine	Gemcitabine	Trametinib	Cisplatin	Lomustine	Etoposide	Radiation
Killing at highest dose	1.00E+00	9.50E-01	-3.30E-01	9.99E-01	7.00E-02	-7.90E-01	7.00E-01	1.00E+00	9.70E-01	7.20E-01	-2.00E-02
EC10	1.17E+02	4.76E+00	NR	2.84E-02	NR	NR	3.23E-02	7.41E+01	1.28E+03	5.26E+00	NR
EC25	1.19E+02	1.89E+01	NR	3.27E-02	NR	NR	8.06E-02	8.71E+01	1.40E+03	2.50E+01	NR
EC50	1.22E+02	2.08E+02	NR	3.98E-02	NR	NR	7.84E-01	1.25E+02	1.61E+03	1.00E+02	NR
EC75	1.25E+02	6.25E+02	NR	4.69E-02	NR	NR	NR	1.88E+02	1.82E+03	NR	NR
EC90	2.13E+02	9.06E+02	NR	5.72E-02	NR	NR	NR	2.25E+02	1.94E+03	NR	NR
Slope	-8.00E-02	-1.20E-03	NR	NR	-1.89E-02	1.56E-02	-2.78E-01	-4.00E-03	-1.20E-03	-3.00E-03	NR
AUC	1.89E+02	3.36E+02	2.58E+03	4.17E-02	1.02E+01	1.74E+02	3.32E+01	6.20E+01	1.96E+03	4.75E+02	4.02E+01
	**LN229**										
	Carboplatin	Azacitidine	Temozolomide	TR107	Vincristine	Gemcitabine	Trametinib	Cisplatin	Lomustine	Etoposide	Radiation
Killing at highest dose	1.00E+00	8.60E-01	3.50E-01	1.00E+00	4.10E-01	2.80E-01	5.60E-01	1.00E+00	1.00E+00	4.40E-01	-3.60E-01
EC10	4.17E+00	2.89E+01	5.94E+01	3.06E-02	6.67E-03	6.23E+00	6.68E-01	7.46E+01	4.17E+02	3.70E+00	NR
EC25	1.40E+01	4.56E+01	4.60E+02	3.44E-02	4.18E-01	8.40E+00	3.46E+00	8.13E+01	7.58E+02	9.26E+00	NR
EC50	1.33E+02	4.00E+02	NR	4.11E-02	NR	NR	1.86E+01	9.24E+01	9.60E+02	NR	NR
EC75	1.93E+02	8.17E+02	NR	4.98E-02	NR	NR	NR	1.36E+02	1.44E+03	NR	NR
EC90	2.29E+02	NR	NR	9.00E-02	NR	NR	NR	2.05E+02	1.78E+03	NR	NR
Slope	-4.20E-03	-6.00E-04	-4.00E-05	-2.87E+01	-8.89E-03	-5.45E-03	-1.47E-02	-2.20E-03	-1.24E-03	-1.73E-04	NR
AUC	1.22E+02	4.51E+02	1.53E+03	6.08E-02	6.37E+00	7.06E+01	4.96E+01	7.69E+01	9.70E+02	6.48E+02	5.01E+01
	**GBM8**										
	Carboplatin	Azacitidine	Temozolomide	TR107	Vincristine	Gemcitabine	Trametinib	Cisplatin	Lomustine	Etoposide	Radiation
Killing at highest dose	1.00E+00	1.00E+00	1.00E+00	1.00E+00	1.00E+00	9.80E-01	9.80E-01	1.00E+00	1.00E+00	1.00E+00	8.69E-01
EC10	1.16E+00	8.33E+00	1.52E+00	6.30E-02	4.35E-03	6.76E-02	3.13E-02	9.06E-02	6.85E-01	5.58E-01	8.33E-01
EC25	2.91E+00	1.81E+01	3.79E+00	7.65E-02	1.29E-02	1.69E-01	7.81E-02	2.26E-01	1.71E+00	1.74E+00	2.04E+00
EC50	5.81E+00	3.38E+01	7.58E+00	9.53E-02	4.86E-02	3.38E-01	3.84E-01	4.53E-01	3.43E+00	4.60E+00	3.84E+00
EC75	8.72E+00	4.94E+01	3.12E+01	1.21E-01	8.43E-02	5.33E-01	7.79E-01	2.74E+00	5.45E+00	7.47E+00	1.37E+01
EC90	2.60E+01	8.33E+01	1.67E+02	1.39E-01	2.60E-01	2.00E+00	1.67E+00	4.20E+00	8.71E+00	9.19E+00	NR
Slope	-8.60E-02	-1.60E-02	-6.60E-02	-1.33E+01	-7.00E+00	-1.48E+00	-6.33E-01	-1.10E+00	-1.46E-01	-8.73E-02	-1.38E-01
AUC	1.03E+01	4.08E+01	9.52E+01	9.94E-02	2.38E-01	2.79E+00	4.78E+00	1.76E-01	1.54E+01	8.40E+00	1.09E+01
	**PDIPG**										
	Carboplatin	Azacitidine	Temozolomide	TR107	Vincristine	Gemcitabine	Trametinib	Cisplatin	Lomustine	Etoposide	Radiation
Killing at highest dose	1.00E+00	6.40E-01	2.90E-01	1.00E+00	4.86E-01	4.80E-01	1.70E-01	1.00E+00	9.30E-01	7.50E-01	3.20E-01
EC10	4.76E+00	3.70E+00	5.00E+00	4.02E-02	2.42E-01	1.14E-01	3.00E+01	3.17E+00	3.54E+02	2.22E+02	1.11E+00
EC25	2.00E+01	9.26E+00	6.67E+02	4.25E-02	4.06E-01	2.84E-01	NR	7.94E+00	4.25E+02	2.42E+02	3.62E+00
EC50	8.61E+01	4.47E+02	1.06E+03	4.64E-02	NR	NR	NR	1.68E+01	6.88E+02	5.74E+02	NR
EC75	2.11E+02	NR	NR	5.19E-02	NR	NR	NR	2.73E+01	1.31E+03	1.00E+03	NR
EC90	5.83E+02	NR	NR	6.57E-02	NR	NR	NR	4.15E+01	1.88E+03	NR	NR
Slope	-3.59E-03	-2.53E-04	-4.80E-05	-6.40E+01	-2.32E-02	-3.03E-04	-1.00E-03	-2.73E-02	-4.80E-04	-5.87E-04	-2.86E-04
AUC	1.79E+02	5.03E+02	1.88E+03	6.60E-02	6.36E+00	5.72E+01	8.73E+01	4.87E+00	9.11E+02	6.75E+02	2.71E+01
	**U373 WT**										
	Carboplatin	Azacitidine	Temozolomide	TR107	Vincristine	Gemcitabine	Trametinib	Cisplatin	Lomustine	Etoposide	Radiation
Killing at highest dose	1.00E+00	5.40E-01	2.20E-01	9.80E-01	-6.60E-01	3.30E-01	4.80E-01	1.00E+00	9.10E-01	3.30E-01	2.60E-01
EC10	9.32E+01	6.11E+01	6.73E+01	1.32E-02	8.20E-01	3.85E-01	6.25E-01	2.03E+01	1.39E+02	2.56E+01	5.88E-01
EC25	1.03E+02	8.19E+01	9.62E+01	3.29E-02	NR	5.00E+01	2.16E+00	2.84E+01	5.54E+02	6.54E+01	3.50E+00
EC50	1.76E+02	8.00E+02	NR	7.65E-02	NR	NR	NR	4.19E+01	6.65E+02	2.60E+02	NR
EC75	2.50E+02	NR	NR	8.93E-02	NR	NR	NR	6.52E+01	1.38E+03	NR	NR
EC90	7.00E+02	NR	NR	9.69E-02	NR	NR	NR	8.79E+01	1.96E+03	NR	NR
Slope	-3.40E-03	-2.00E-04	-9.14E-06	-1.96E+01	5.78E-02	-1.60E-03	-2.50E-04	-1.85E-02	-2.24E-03	-1.26E-04	-6.50E-03
AUC	3.11E+02	5.88E+02	1.52E+03	6.00E-02	1.36E+01	7.29E+01	5.40E+01	1.33E+01	8.89E+02	6.12E+02	2.82E+01
	**U373 KO**										
	Carboplatin	Azacitidine	Temozolomide	TR107	Vincristine	Gemcitabine	Trametinib	Cisplatin	Lomustine	Etoposide	Radiation
Killing at highest dose	9.99E-01	8.30E-01	5.40E-01	9.50E-01	-7.40E-01	6.20E-01	3.10E-01	1.00E+00	9.70E-01	6.38E-01	-1.70E-01
EC10	9.09E+00	2.38E+01	3.85E+00	5.67E-02	NR	1.39E-01	4.58E+01	3.32E+01	8.33E+01	2.42E+00	NR
EC25	1.25E+02	4.45E+01	9.62E+00	6.02E-02	NR	3.47E-01	8.45E+01	3.65E+01	2.08E+02	6.06E+00	NR
EC50	1.72E+02	1.58E+02	1.16E+03	6.62E-02	NR	8.68E-01	NR	4.20E+01	6.07E+02	3.78E+01	NR
EC75	2.19E+02	6.47E+02	NR	7.21E-02	NR	NR	NR	4.76E+01	1.46E+03	NR	NR
EC90	2.48E+02	NR	NR	8.33E-02	NR	NR	NR	1.14E+02	1.83E+03	NR	NR
Slope	-5.29E-03	-1.73E-03	-8.67E-04	-4.20E+01	NR	-3.80E-01	-3.88E-03	-4.53E-02	-5.60E-04	-3.13E-03	4.25E-03
AUC	1.92E+02	3.39E+02	8.89E+02	9.00E-02	1.89E+01	3.67E+01	8.91E+01	1.56E+01	8.35E+02	3.37E+02	4.54E+01
	**MS21**										
	Carboplatin	Azacitidine	Temozolomide	TR107	Vincristine	Gemcitabine	Trametinib	Cisplatin	Lomustine	Etoposide	Radiation
Killing at highest dose	1.00E+00	5.50E-01	-8.20E-01	9.68E-01	5.30E-01	-1.20E-01	-5.00E-02	9.99E-01	9.10E-01	-8.00E-02	5.00E-02
EC10	5.97E+01	3.15E+01	-5.00E+02	5.74E-02	5.26E-03	NR	NR	4.55E+00	1.12E+03	NR	NR
EC25	7.18E+01	9.09E+01	4.17E+02	7.04E-02	1.72E-01	NR	NR	1.16E+01	1.28E+03	NR	NR
EC50	9.19E+01	8.21E+02	8.33E+02	8.26E-02	4.60E+00	NR	NR	2.47E+01	1.55E+03	NR	NR
EC75	1.57E+02	NR	1.25E+03	9.23E-02	NR	NR	NR	3.79E+01	1.83E+03	NR	NR
EC90	2.15E+02	NR	1.50E+03	9.82E-02	NR	NR	NR	4.57E+01	1.99E+03	NR	NR
Slope	-1.24E-02	-2.80E-04	5.47E-04	-2.58E+01	-5.56E-03	NR	NR	-1.90E-02	-9.20E-04	NR	NR
AUC	1.47E+02	6.06E+02	2.37E+03	8.81E-02	5.22E+00	1.06E+02	1.26E+02	4.20E+00	1.61E+03	1.15E+03	4.25E+01

Table S1, Related to Figure 3 and 4: Killing Parameters on OBSCs. Dose-response values used to generate DSS from each cell line against each therapeutic. Values for Killing at Highest Dose are given as fraction of tumor population killed. EC values are given as small molecule drug concentrations in μM or as XRad dose in Gy. NR indicates the IC50 was not reached within the dose range. Linear interpolation between calculated data points used to calculate individual EC values. Trapezoidal rule used to calculate AUC.

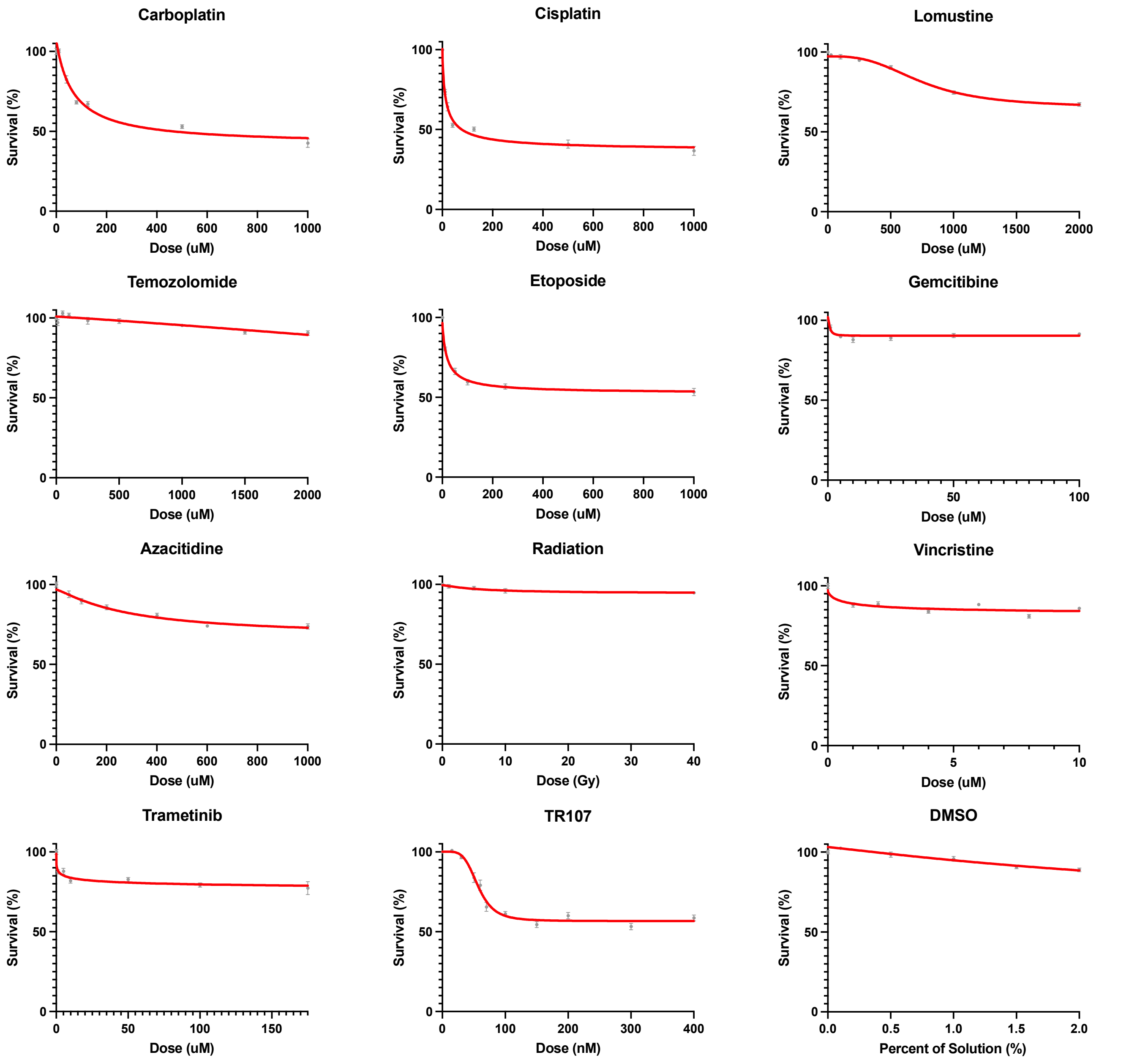

Figure S3, Related to Figure 4: OBSC Treatment Toxicity. Dose-response curves of OBSC survival via PI stain against each therapeutic. To enable qualitative comparison, all datasets are fit with an inhibitor vs response variable slope (four parameters) least squares fit. This method poorly fits some curve types, such as those with increases in growth at low concentrations. Our IC50 and DSS calculations do not use these fitted curves, but instead use linear interpolation between calculated data points.

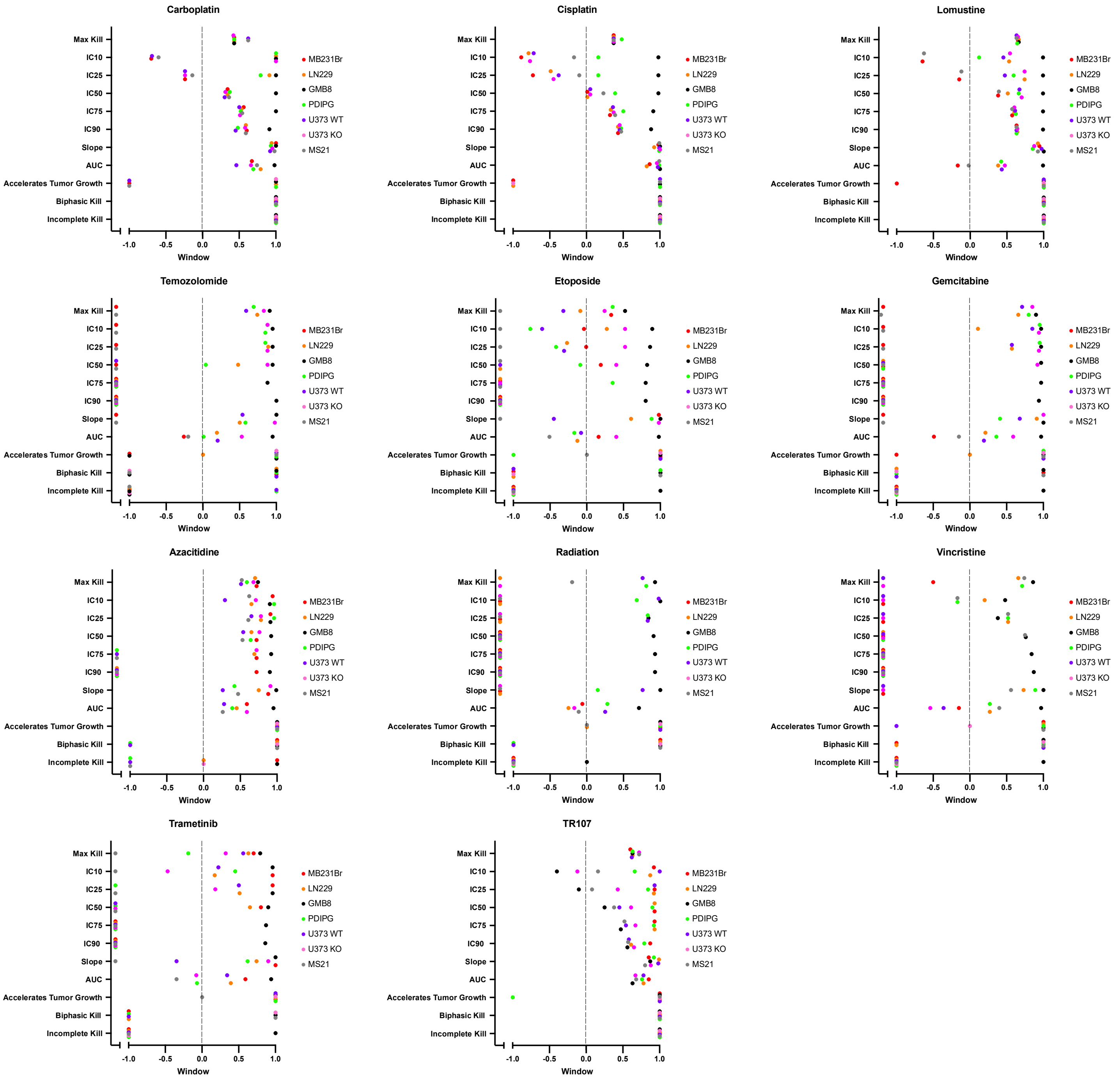

Figure S4, Related to Figure 4: DSS Window Plots. Therapeutic windows across all 11 DSS parameters for all drugs vs all tumor lines. Windows closer to 1 indicate more selective killing of the tumor. Windows closer to -1 indicate more selective killing of the OBSC. Windows left of -1 indicate the parameter was never reached. Windows for each parameter were weighted and summed to calculate a single DSS for each drug-tumor-OBSC interaction. Windows were calculated via linear interpolation of raw data, not from best-fit curve equations.

Top 250 Most Significantly Mutated Somatic Genes - From MMG-II DNAseq									
1	CCL4L2	51	USP2	101	ADGRB3	151	NDNF	201	TRIM41
2	FLG2	52	WASHC5	102	AGK	152	NPIPB5	202	TRPM2
3	NF2	53	XKR9	103	ANKRD18A	153	NRXN1	203	UBE2J1
4	ANKRD36	54	ZNF518A	104	ANKRD33B	154	NRXN2	204	UGT2A1
5	IGHV3-30	55	CENPB	105	AP4B1	155	NTN1	205	USP6
6	IGHV3-38	56	GNAS	106	APBB2	156	NUP160	206	WDFY4
7	TRBV6-8	57	IGHV3-11	107	BBS7	157	NUP58	207	WDR24
8	KCNE1B	58	IGHV3-53	108	BCL11A	158	OR4C45	208	WDR87
9	MUC4	59	MCU	109	BOP1	159	OR4Q3	209	WWC2
10	OR4M2B	60	NIBAN3	110	BRD8	160	PCDHB10	210	XPC
11	AGAP1	61	NID1	111	C10orf88	161	PDZRN4	211	ZNF138
12	AP000721.1	62	PRAMEF9	112	CDC45	162	PIGG	212	ZNF208
13	ARHGAP21	63	ACSL4	113	CELSR3	163	PLEKHG6	213	ZNF408
14	ARID4B	64	BACE1	114	CIT	164	POLD1	214	ZNF540
15	BHLHE41	65	CCZ1B	115	COL12A1	165	POLE	215	AGAP9
16	COLQ	66	IRS2	116	DMXL1	166	PPP1R12A	216	AMBRA1
17	CRLF1	67	KMT2B	117	DOCK6	167	PRAMEF14	217	ANKRD36B
18	DHCR7	68	MUC5AC	118	DOCK7	168	PRDM11	218	ATXN1L
19	DHX8	69	AGFG1	119	DRG2	169	PRPF4	219	BHLHA9
20	EIF3M	70	BNC2	120	EIF4G2	170	RAB1A	220	CASR
21	FAM160B2	71	C5orf34	121	EVC2	171	RELCH	221	CAT
22	FAM47A	72	C6orf62	122	FLG	172	RNF212B	222	CCDC15
23	FN1	73	CACNA1D	123	FO681492.1	173	RPAP3	223	CDIN1
24	GABRR1	74	CDS1	124	FUT11	174	RPGRIP1L	224	CKB
25	GTF3C2	75	DNAH1	125	GOLGA6L1	175	RSF1	225	CRAMP1
26	HIBCH	76	EPS8L2	126	GOLGA6L9	176	RSPH1	226	CSMD3
27	HIRIP3	77	FOXO6	127	GPN1	177	RWDD3	227	CYLC1
28	HIVEP1	78	GLI3	128	GTPBP8	178	SCN10A	228	DSCAML1
29	HSF4	79	IFFO1	129	HNRNPLL	179	SERAC1	229	ENOX2
30	KIAA0319	80	KANSL2	130	HOXD8	180	SERINC4	230	EPC2
31	LILRA6	81	LZTS1	131	HRC	181	SH3TC2	231	EPG5
32	MAP3K6	82	MSI2	132	HTR2B	182	SLC12A6	232	ERC1
33	OR51B6	83	NBPF14	133	IGHV2-70D	183	SLC13A4	233	FAM120B
34	OR5L2	84	NCOA6	134	IGHV4-4	184	SLC18A1	234	FBXO38
35	P3H2	85	NOVA1	135	ITIH6	185	SLC7A4	235	FHOD1
36	PLEKHS1	86	NPIPA5	136	IVD	186	SOX21	236	FOXN3
37	PLPPR3	87	NSD1	137	KANSL1	187	SOX3	237	HAT1
38	PRR29	88	PCDH19	138	KCNIP4	188	SPATA7	238	HERPUD1
39	PSG3	89	PTCHD4	139	KIAA1549	189	SULT1A1	239	HIPK1
40	RRAGC	90	PUM1	140	KIR3DL3	190	SYBU	240	HOXC11
41	RTN3	91	RALGAPA1	141	LAMC1	191	TBC1D3B	241	HPS1
42	RTP3	92	RNF11	142	LILRB3	192	TBXT	242	IL1RAPL2
43	SCAF11	93	RSPO4	143	LTN1	193	TEX10	243	KAT6B
44	SPTBN2	94	SLF1	144	LYSMD4	194	THADA	244	KIR2DL4
45	ST6GALNAC3	95	TRBV6-5	145	MBD5	195	TMEM164	245	KMT2A
46	SYNJ2	96	WNK2	146	MIDEAS	196	TMEM267	246	KMT2D
47	TMEM272	97	ZNF326	147	MTF2	197	TMPRSS15	247	KRTAP10-7
48	TRMT10B	98	AARS2	148	MYT1L	198	TNK2	248	MSL1
49	TYRO3	99	ABCA13	149	NBPF20	199	TNKS1BP1	249	PHF12
50	UNC93A	100	ADAM30	150	NBPF26	200	TOX	250	TCERG1

Table S2, Related to Figure 5: Top 250 Most Significantly Mutated Somatic Genes – From MG-II DNAseq.

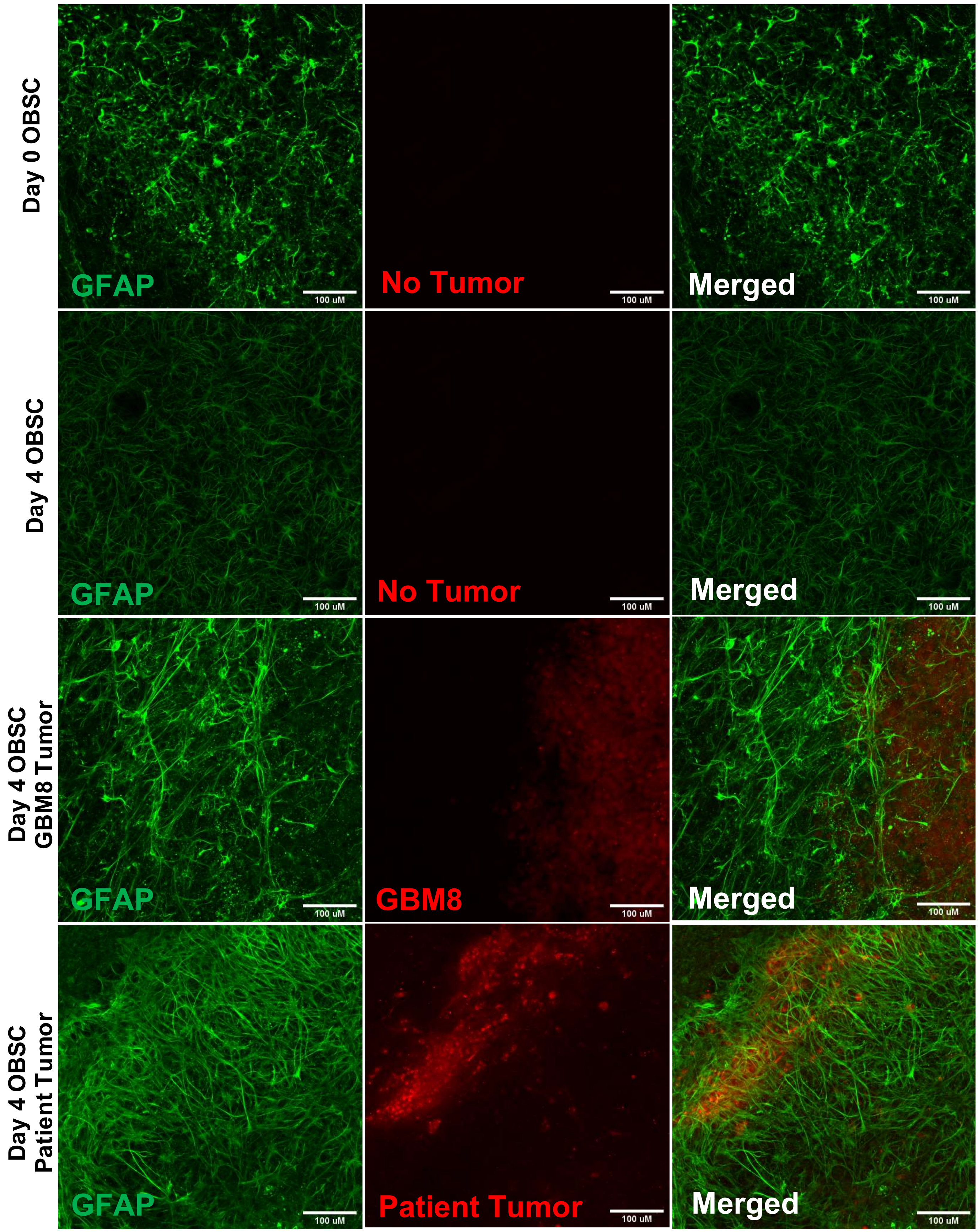

Figure S5, Related to Figures 1, 2, and 5: High-res IHC Images: High-resolution versions of IHC images shown in Figures 1, 2, and 5.

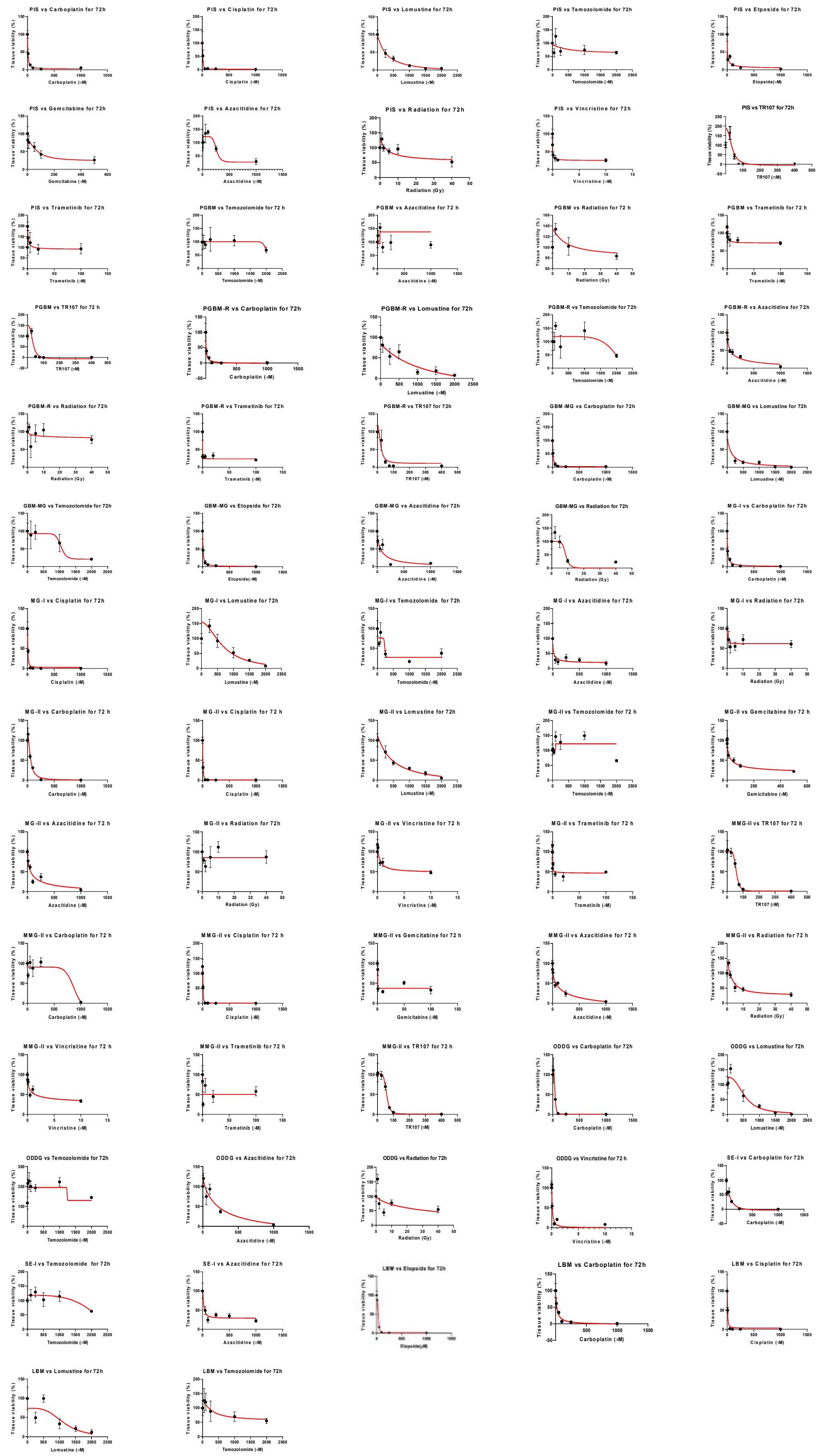

Figure S6, Related to Figure 7: Dose Response Curves for Patient Tissue Dose-response curves of each patient tumor against given therapeutic. To enable qualitative comparison, all datasets are fit with an inhibitor vs response variable slope (four parameters) least squares fit. This method poorly fits some curve types, such as those with increases in growth at low concentrations. Our IC50 and DSS calculations do not use these fitted curves, but instead use linear interpolation between calculated data points.

	**PIS**										
	**Carboplatin**	**Azacitidine**	**Temozolomide**	**TR107**	**Vincristine**	**Gemcitabine**	**Trametinib**	**Cisplatin**	**Lomustine**	**Etoposide**	**Radiation**
**Killing at highest dose**	9.47E-01	7.08E-01	3.58E-01	9.65E-01	7.41E-01	5.80E-01	6.58E-02	9.80E-01	9.55E-01	9.75E-01	4.75E-01
**EC10**	1.85E+00	2.19E+02	8.24E+01	3.99E-02	3.26E-03	2.78E-01	NR	2.09E+00	4.70E+01	1.40E+00	4.39E+00
**EC25**	4.62E+00	2.82E+02	2.06E+02	4.30E-02	8.16E-03	1.30E+01	NR	5.21E+00	1.17E+02	3.51E+00	1.77E+01
**EC50**	9.24E+00	6.74E+02	NR	4.81E-02	7.18E-02	8.10E+01	NR	1.17E+01	2.35E+02	7.02E+00	NR
**EC75**	3.62E+01	NR	NR	6.03E-02	NR	NR	NR	3.24E+01	6.78E+02	3.27E+01	NR
**EC90**	7.37E+01	NR	NR	7.03E-02	NR	NR	NR	4.49E+01	1.14E+03	1.74E+02	NR
**Slope**	-5.41E-02	-6.37E-04	-3.10E-05	-4.92E+01	-3.13E+00	-4.24E-03	-3.04E-03	-1.21E-02	-5.88E-04	-7.12E-02	-1.01E-02
**AUC**	5.93E+01	2.45E+02	1.47E+03	7.25E-02	2.71E+00	6.21E+01	9.67E+01	4.95E+01	4.56E+02	7.95E+01	1.47E+01
	**PGBM**										
	**Carboplatin**	**Azacitidine**	**Temozolomide**	**TR107**	**Vincristine**	**Gemcitabine**	**Trametinib**	**Cisplatin**	**Lomustine**	**Etoposide**	**Radiation**
**Killing at highest dose**		1.03E-01	3.07E-01	9.86E-01			2.80E-01				1.66E-01
**EC10**		9.32E+01	1.43E+03	3.22E-02			9.66E-01				2.93E+01
**EC25**		NR	1.84E+03	3.53E-02			7.00E+01				NR
**EC50**		NR	NR	4.06E-02			NR				NR
**EC75**		NR	NR	4.59E-02			NR				NR
**EC90**		NR	NR	4.90E-02			NR				NR
**Slope**		-1.08E-04	-3.60E-04	-4.74E+01			-1.00E-03				-6.12E-03
**AUC**		9.62E+02	1.92E+03	4.56E-02			7.73E+01				3.96E+01
	**PGBM-R**										
	**Carboplatin**	**Azacitidine**	**Temozolomide**	**TR107**	**Vincristine**	**Gemcitabine**	**Trametinib**	**Cisplatin**	**Lomustine**	**Etoposide**	**Radiation**
**Killing at highest dose**	9.91E-01	9.55E-01	5.33E-01	9.63E-01			7.92E-01		9.23E-01		2.23E-01
**EC10**	1.64E+00	5.21E+00	1.50E+02	1.04E-02			1.43E-02		5.43E+01		2.65E+01
**EC25**	4.10E+00	1.70E+01	5.22E+02	2.54E-02			3.58E-02		1.36E+02		NR
**EC50**	8.20E+00	4.73E+01	1.83E+03	3.57E-02			7.16E-02		3.26E+02		NR
**EC75**	3.56E+01	4.54E+02	NR	4.59E-02			7.22E+01		8.06E+02		NR
**EC90**	7.20E+01	8.54E+02	NR	6.12E-02			NR		1.68E+03		NR
**Slope**	-6.10E-02	-8.26E-03	-1.91E-04	-2.44E+01			-6.98E+00		-5.21E-04		-9.12E-03
**AUC**	2.72E+01	2.56E+02	2.06E+03	4.84E-02			2.77E+01		4.89E+02		3.66E+01
	**GBM-MG**										
	**Carboplatin**	**Azacitidine**	**Temozolomide**	**TR107**	**Vincristine**	**Gemcitabine**	**Trametinib**	**Cisplatin**	**Lomustine**	**Etoposide**	**Radiation**
**Killing at highest dose**	9.72E-01	9.01E-01	7.89E-01						9.96E-01	9.94E-01	7.70E-01
**EC10**	2.12E+00	3.64E+00	9.05E+01						3.04E+01	1.87E+00	1.37E+00
**EC25**	5.30E+00	9.11E+00	6.68E+02						7.61E+01	4.68E+00	6.64E+00
**EC50**	1.27E+01	1.33E+02	1.37E+03						1.52E+02	9.36E+00	8.39E+00
**EC75**	3.65E+01	2.00E+02	1.91E+03						2.28E+02	3.50E+01	2.48E+01
**EC90**	5.55E+01	2.40E+02	NR						1.15E+03	6.65E+01	NR
**Slope**	-1.05E-02	-3.73E-03	-4.58E-04						-3.29E-03	-5.34E-02	-1.43E-01
**AUC**	4.77E+01	1.73E+02	1.25E+03						-2.94E+00	4.37E+01	7.60E+00
	**MG-I**										
	**Carboplatin**	**Azacitidine**	**Temozolomide**	**TR107**	**Vincristine**	**Gemcitabine**	**Trametinib**	**Cisplatin**	**Lomustine**	**Etoposide**	**Radiation**
**Killing at highest dose**	9.88E-01	8.29E-01	6.20E-01					9.94E-01	9.19E-01		3.91E-01
**EC10**	1.79E+00	6.99E+00	1.02E+02					1.78E+00	5.27E+02		3.64E-01
**EC25**	4.49E+00	1.75E+01	1.43E+02					4.44E+00	7.17E+02		9.09E-01
**EC50**	8.97E+00	3.50E+01	2.12E+02					8.88E+00	1.05E+03		6.92E+01
**EC75**	4.31E+01	6.46E+02	NR					2.78E+01	1.55E+03		NR
**EC90**	8.41E+01	NR	NR					4.20E+01	1.95E+03		NR
**Slope**	-5.57E-02	-1.43E-02	-3.62E-03					-5.63E-02	-5.13E-04		-3.74E-03
**AUC**	4.42E+01	1.82E+02	6.50E+02					2.04E+01	1.24E+03		2.63E+01
	**MG-II**										
	**Carboplatin**	**Azacitidine**	**Temozolomide**	**TR107**	**Vincristine**	**Gemcitabine**	**Trametinib**	**Cisplatin**	**Lomustine**	**Etoposide**	**Radiation**
**Killing at highest dose**	9.95E-01	9.49E-01	3.46E-01	9.90E-01	5.22E-01	6.41E-01	5.11E-01	9.99E-01	9.44E-01		1.29E-01
**EC10**	2.82E+01	4.31E+00	3.53E+02	5.32E-02	3.12E-01	1.01E+00	2.39E-02	1.47E+00	8.58E+01		3.64E+00
**EC25**	3.90E+01	1.47E+01	1.36E+03	5.75E-02	9.64E-01	5.89E+00	5.98E-02	3.67E+00	2.15E+02		NR
**EC50**	6.70E+01	1.43E+02	NR	6.48E-02	9.21E+00	5.01E+01	2.85E+00	7.33E+00	4.39E+02		NR
**EC75**	1.31E+02	5.29E+02	NR	7.20E-02	NR	NR	NR	1.87E+01	1.19E+03		NR
**EC90**	2.06E+02	8.84E+02	NR	8.44E-02	NR	NR	NR	3.80E+01	1.81E+03		NR
**Slope**	-5.68E-03	-1.24E-03	-1.49E-04	-3.45E+01	-2.86E-02	-3.08E-03	-6.41E-02	-6.82E-02	-1.10E-03		-3.23E-03
**AUC**	1.03E+02	2.61E+02	2.69E+03	6.75E-02	1.93E+00	5.24E+01	4.37E+01	1.70E+01	7.14E+02		3.87E+01
	**MMG-II**										
	**Carboplatin**	**Azacitidine**	**Temozolomide**	**TR107**	**Vincristine**	**Gemcitabine**	**Trametinib**	**Cisplatin**	**Lomustine**	**Etoposide**	**Radiation**
**Killing at highest dose**	9.73E-01	9.58E-01		9.93E-01	6.61E-01	6.69E-01	5.74E-01	9.98E-01			7.20E-01
**EC10**	9.31E+01	4.40E+00		3.21E-02	7.86E-03	3.24E-01	4.73E-02	2.14E+00			2.33E+00
**EC25**	2.37E+02	1.28E+01		4.76E-02	1.78E-01	5.99E-01	2.07E+00	5.34E+00			3.39E+00
**EC50**	5.01E+02	4.40E+01		5.95E-02	4.84E-01	8.59E-01	1.58E+01	1.24E+01			7.03E+00
**EC75**	7.65E+02	2.42E+02		7.14E-02	NR	NR	NR	3.14E+01			NR
**EC90**	9.23E+02	7.81E+02		9.01E-02	NR	NR	NR	4.28E+01			NR
**Slope**	-9.49E-04	-8.02E-03		-2.10E+01	-8.17E-01	-9.63E-01	-1.78E-02	-1.32E-02			-1.14E-02
**AUC**	6.31E+02	2.19E+02		7.04E-02	4.98E+00	4.08E+01	4.98E+01	2.10E+01			1.82E+01
	**ODDG**										
	**Carboplatin**	**Azacitidine**	**Temozolomide**	**TR107**	**Vincristine**	**Gemcitabine**	**Trametinib**	**Cisplatin**	**Lomustine**	**Etoposide**	**Radiation**
**Killing at highest dose**	9.95E-01	9.61E-01	-4.46E-01		9.17E-01				9.86E-01		4.53E-01
**EC10**	2.15E+01	3.66E+01	NR		2.20E-02				1.33E+02		7.82E-01
**EC25**	2.98E+01	4.99E+01	NR		6.54E-02				3.33E+02		1.96E+00
**EC50**	4.35E+01	1.81E+02	NR		2.18E-01				6.81E+02		NR
**EC75**	6.83E+01	5.22E+02	NR		6.41E+00				1.07E+03		NR
**EC90**	8.94E+01	8.61E+02	NR		8.74E+00				1.40E+03		NR
**Slope**	-1.82E-02	-1.89E-03	2.23E-04		-3.76E-01				-6.90E-04		-7.52E-03
**AUC**	5.85E+01	3.43E+02	3.73E+03		1.24E-01				9.66E+02		9.09E+00
	**SE-I**										
	**Carboplatin**	**Azacitidine**	**Temozolomide**	**TR107**	**Vincristine**	**Gemcitabine**	**Trametinib**	**Cisplatin**	**Lomustine**	**Etoposide**	**Radiation**
**Killing at highest dose**	9.85E-01	8.04E-01	3.44E-01								
**EC10**	2.16E+00	2.04E+00	1.50E+03								
**EC25**	5.39E+00	5.11E+00	1.81E+03								
**EC50**	6.41E+01	1.69E+01	NR								
**EC75**	NR	7.23E+02	NR								
**EC90**	NR	NR	NR								
**Slope**	-6.39E-03	-1.50E-03	-4.93E-04								
**AUC**	7.32E+01	2.95E+02	2.00E+03								
	**LBM**										
	**Carboplatin**	**Azacitidine**	**Temozolomide**	**TR107**	**Vincristine**	**Gemcitabine**	**Trametinib**	**Cisplatin**	**Lomustine**	**Etoposide**	**Radiation**
**Killing at highest dose**	9.91E-01		4.48E-01					9.95E-01	8.79E-01	9.90E-01	
**EC10**	2.57E+00		2.42E+02					1.99E+00	4.98E+01	7.83E+00	
**EC25**	6.41E+00		5.19E+01					4.98E+00	1.25E+02	1.68E+01	
**EC50**	2.65E+01		NR					9.97E+00	2.49E+02	3.08E+01	
**EC75**	6.72E+01		NR					1.52E+01	1.35E+03	4.48E+01	
**EC90**	9.47E+01		NR					1.83E+01	NR	7.10E+01	
**Slope**	-6.66E-03		-1.50E-04					-5.02E-02	-2.01E-03	-1.79E-02	
**AUC**	6.97E+01		1.50E+03					1.46E+01	9.28E+02	4.31E+01	

Table S3, Related to Figure 7: Killing Parameters on OBSCs. Dose-response values used to generate DSS from each cell line against each therapeutic. Values for Killing at Highest Dose are given as fraction of tumor population killed. EC values are given as small molecule drug concentrations in µM or as XRad dose in Gy. NR indicates the IC50 was not reached within the dose range. Linear interpolation between calculated data points used to calculate individual EC values. Trapezoidal rule used to calculate AUC.

Tumor Name	Primary pathology diagnosis	Age	Mutations	Treatment
PGBM	GBM	Pediatric	IDH1/2 IDH1 c.395G>A mutation detected, TERT-, methylated MGMT, GFAP+, OLIG2+, IDH1132H+, H3K27M-, patchy expression of BRAF V600E, MS-Stable, IDH1 = R132H, PIK3CA = H1047R, CDK4 amplification, PAX5 = V129M, TP53 = R273C	Xrad, Veliparib, TMZ
MG-I	WHO I meningioma	Adult	Synaptophysin+, GATA3+, growth hormone-, ACTH-, prolactin-	No Further Treatment
GBM-MG	GBM w/ WHO I meningioma	Adult	GBM: GFAP+, rare positivity for OLIG2, IDH1-, ATRX-, p53-, p16-, Ki-67 5-10%. WHO I meningioma: GFAP-,	TMZ (treatment delayed)
LBM	Stage III NSCLC (adenocarcinoma)	Adult	Mock 31+, CK7+, rare scattered cells positive for TTF-1 and CDX-2, Napsin-A-, GATA 3-, CK20-, p40-, PAX 8-,	Xrad; GammaTiles internal radiation
PIS	Intracranial sarcoma	Pediatric	DICER1-mutant+	Xrad, etoposide, ICE
SE-I	WHO I subependymoma	Adult	GFAP+, EMA+, Ki-67 <1%	No Further Treatment
PGBM-R	GBM (Recurrent tumor from same patient as PGBM)	Pediatric	MS-stable, CDK4 amplification, IDH1 R132H, PAX5 V129M, PIK3CA H1047R, TP53 R273C	TMZ; REMIND Trial: associated antigen T-cell infusion
MG-II	WHO grade II meningioma	Adult	EMA+ with subset of cells, GFAP+, Ki-67+ with proliferation index at 15-20 percent	Xrad
MMG-II	WHO grade II meningioma (multiple tumors)	Adult	Ki-67+ with proliferation index at 5-10 percent	Xrad
ODDG	WHO grade III oligodendroglioma	Adult	GFAP+, OLGI2+, IDH1 R132H+, MGMT+, ATRX+, P16-, P53-, Positive for TERT promoter -146C>T (C250T) mutation, Abnormal FISH Result Consistent with Codeletion of 1p and 19q	TMZ + Xrad
GG-I	WHO grade I ganglioglioma	Adult	GFAP+, no immunohistochemical evidence of alterations in IDH1, BRAF, or H3 K27M, and low Ki-67 proliferation index	No Further Treatment

Table S4, Related to Figure 7: Patient Tumor Clinical Data. Data was derived from clinical records in accord with our IRB-approved protocol.

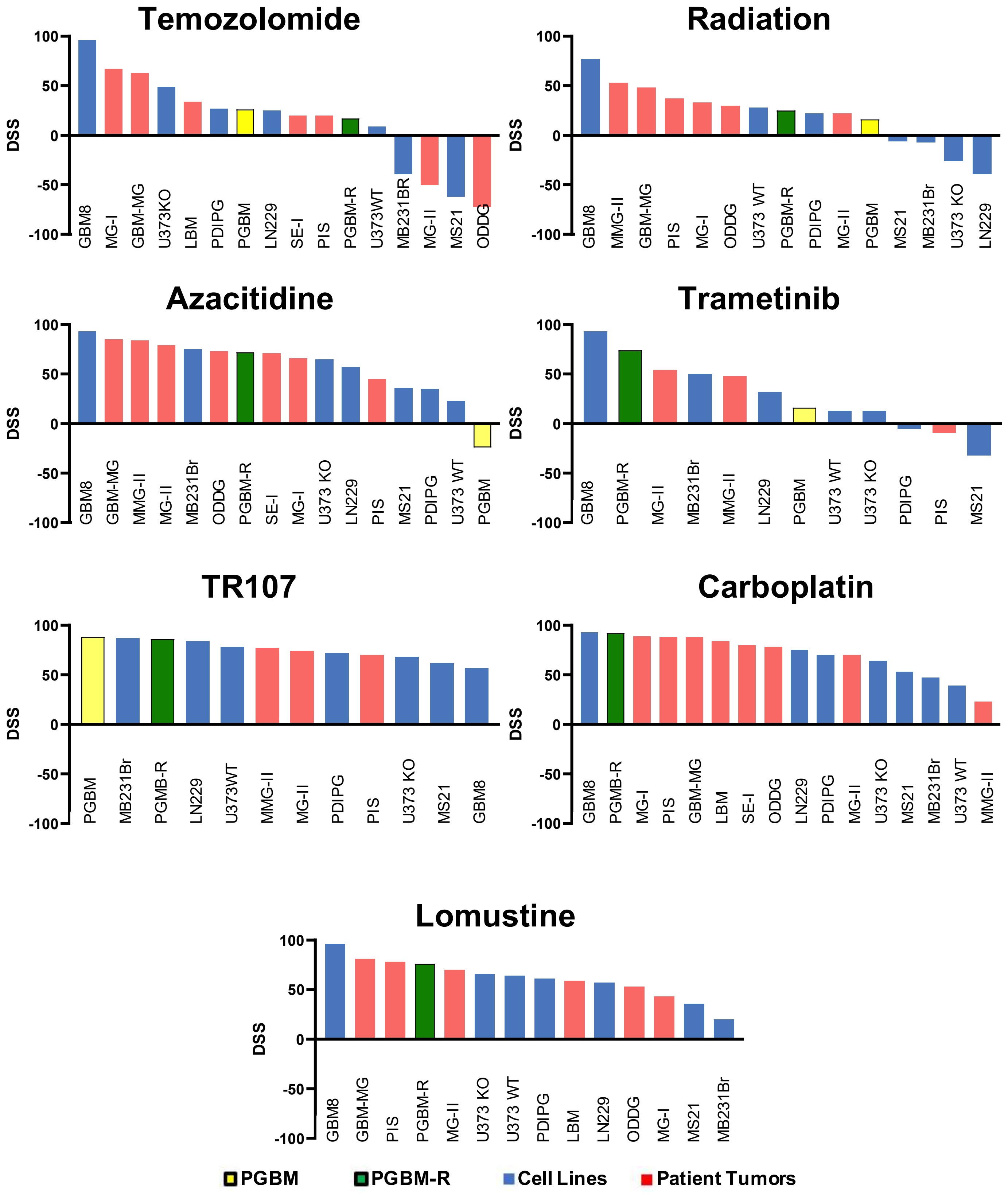

Figure S7, Related to Figure 7: Waterfall Plots with All Tumor IDs. Waterfall plots depicting relative sensitivities to individual therapeutics. All tumors treated with each therapeutic are included and identified on x-axis. Yellow lines with black outline = DSS for PGBM; green lines with black outline = DSS for PGBM-R; blue lines = DSS for established tumor lines; red lines = DSS for patient tumor tissue.
