# Supplementary material for: A living ex vivo platform for functional, personalized brain cancer diagnosis
Source: Cell Rep Med. 2023 May 15;4(6):101042. doi: 10.1016/j.xcrm.2023.101042 (PMC10313921; doi:10.1016/j.xcrm.2023.101042)
Supplement: Document S2. Article plus supplemental information [file mmc2.pdf]

Cell Reports
Medicine

Article

A living ex vivo platform for functional, personalized brain cancer diagnosis

Graphical abstract

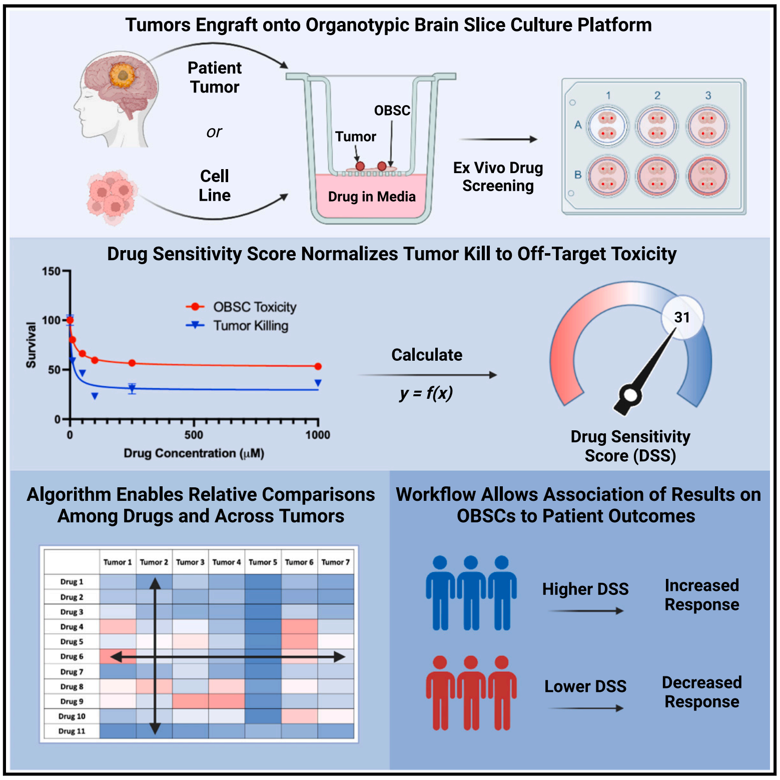

Highlights

- OBSCs allow rapid drug screening of cell lines and uncultured patient tumor tissue
- Drug sensitivity score normalizes tumor kill to off-target toxicity for each drug
- Algorithm enables relative comparisons among drugs and across tumors
- Workflow allows association of results on OBSCs to patient outcomes

Authors

Breanna Mann, Xiaopei Zhang, Noah Bell, ..., Albert Baldwin, Shawn Hingtgen, Andrew B. Satterlee

Correspondence

hingtgen@email.unc.edu (S.H.), satterle@email.unc.edu (A.B.S.)

In brief

Mann et al. develop an organotypic brain slice culture (OBSC)-based platform and multi-parametric algorithm that enable rapid engraftment, treatment, and analysis of uncultured patient brain tumor tissue and patient-derived cell lines. After treatment, the algorithm calculates summarized drug sensitivity scores, allowing relative comparisons that show positive associations to clinical outcomes.

Mann et al., 2023, Cell Reports Medicine *4*, 101042
June 20, 2023 © 2023 The Author(s).
https://doi.org/10.1016/j.xcrm.2023.101042

CellPress

Cell Reports Medicine

CellPress
OPEN ACCESS

Article

A living ex vivo platform for functional, personalized brain cancer diagnosis

Breanna Mann,[1,14] Xiaopei Zhang,[1,14] Noah Bell,[1] Adebimpe Adefolaju,[2] Morrent Thang,[3] Rajaneekar Dasari,[2] Krishna Kanchi,[4] Alain Valdivia,[1] Yang Yang,[5,6] Andrew Buckley,[1] Vivien Lettry,[1] Carolyn Quinsey,[7] Yasmeen Rauf,[7,8] David Kram,[9] Noah Cassidy,[10] Cyrus Vaziri,[5,6] David L. Corcoran,[4] Stephen Rego,[6] Yuchao Jiang,[4,11] Lee M. Graves,[12] Denise Dunn,[13] Scott Floyd,[13] Albert Baldwin,[6] Shawn Hingtgen,[1,*] and Andrew B. Satterlee[1,2,15,*]

[1]Eshelman School of Pharmacy, Division of Pharmacoengineering and Molecular Pharmaceutics, University of North Carolina at Chapel Hill, Chapel Hill, NC, USA
[2]Eshelman Institute for Innovation, University of North Carolina at Chapel Hill, Chapel Hill, NC, USA
[3]Department of Neuroscience, University of North Carolina at Chapel Hill, Chapel Hill, NC, USA
[4]Department of Genetics, University of North Carolina at Chapel Hill, Chapel Hill, NC, USA
[5]Department of Pathology and Laboratory Medicine, University of North Carolina at Chapel Hill, Chapel Hill, NC, USA
[6]Lineberger Comprehensive Cancer Center, University of North Carolina at Chapel Hill, Chapel Hill, NC, USA
[7]Department of Neurosurgery, University of North Carolina at Chapel Hill, Chapel Hill, NC, USA
[8]Department of Neurology, University of North Carolina at Chapel Hill, Chapel Hill, NC, USA
[9]Division of Pediatric Hematology-Oncology, University of North Carolina at Chapel Hill, Chapel Hill, NC, USA
[10]School of Medicine, University of North Carolina at Chapel Hill, Chapel Hill, NC, USA
[11]Department of Biostatistics, University of North Carolina at Chapel Hill, Chapel Hill, NC, USA
[12]Department of Pharmacology, University of North Carolina at Chapel Hill, Chapel Hill, NC, USA
[13]Department of Radiation Oncology, Duke University Medical Center, Durham, NC, USA
[14]These authors contributed equally
[15]Lead contact
*Correspondence: hingtgen@email.unc.edu (S.H.), satterle@email.unc.edu (A.B.S.)
https://doi.org/10.1016/j.xcrm.2023.101042

SUMMARY

Functional precision medicine platforms are emerging as promising strategies to improve pre-clinical drug testing and guide clinical decisions. We have developed an organotypic brain slice culture (OBSC)-based platform and multi-parametric algorithm that enable rapid engraftment, treatment, and analysis of uncultured patient brain tumor tissue and patient-derived cell lines. The platform has supported engraftment of every patient tumor tested to this point: high- and low-grade adult and pediatric tumor tissue rapidly establishes on OBSCs among endogenous astrocytes and microglia while maintaining the tumor's original DNA profile. Our algorithm calculates dose-response relationships of both tumor kill and OBSC toxicity, generating summarized drug sensitivity scores on the basis of therapeutic window and allowing us to normalize response profiles across a panel of U.S. Food and Drug Administration (FDA)-approved and exploratory agents. Summarized patient tumor scores after OBSC treatment show positive associations to clinical outcomes, suggesting that the OBSC platform can provide rapid, accurate, functional testing to ultimately guide patient care.

INTRODUCTION

The accurate classification of CNS tumors has made significant progress in the past two decades, moving from an exclusive reliance on histopathological features toward more integrated diagnoses based on molecular insights.[1] Although the improved classification of CNS tumors is already enhancing the scientific rigor of clinical trials, the impact of these integrated diagnoses on precision oncology medicine—the molecular-guided pairing of tumors with drugs—has yet to demonstrate widespread clinical benefit. Between 2006 and 2020, a time period during which an astounding number of new cancer-directed drugs were developed, eligibility for those drugs only increased from about 5% to 13%, and response to those drugs only increased from about 3% to 7%.[2] The reasons for this are myriad and include factors such as co-occurring oncogenic alterations,[3] tumor heterogeneity,[4] epistatic interactions,[5] and adaptive cellular circuitry.[6–8] It is becoming increasingly clear that static histopathological and molecular measurements of tumors are still insufficient to most accurately and usefully classify CNS tumors and that to identify precision medicines for the majority of CNS tumor patients, *functional* diagnostic platforms are needed.

Patient-derived models of cancer (PDMCs), including cell lines, patient-derived organoids (PDOs), patient-derived explants (PDEs), and patient-derived xenografts (PDXs), provide functional models of a patient's individual tumor that can be screened

This is an open access article under the CC BY license (http://creativecommons.org/licenses/by/4.0/).

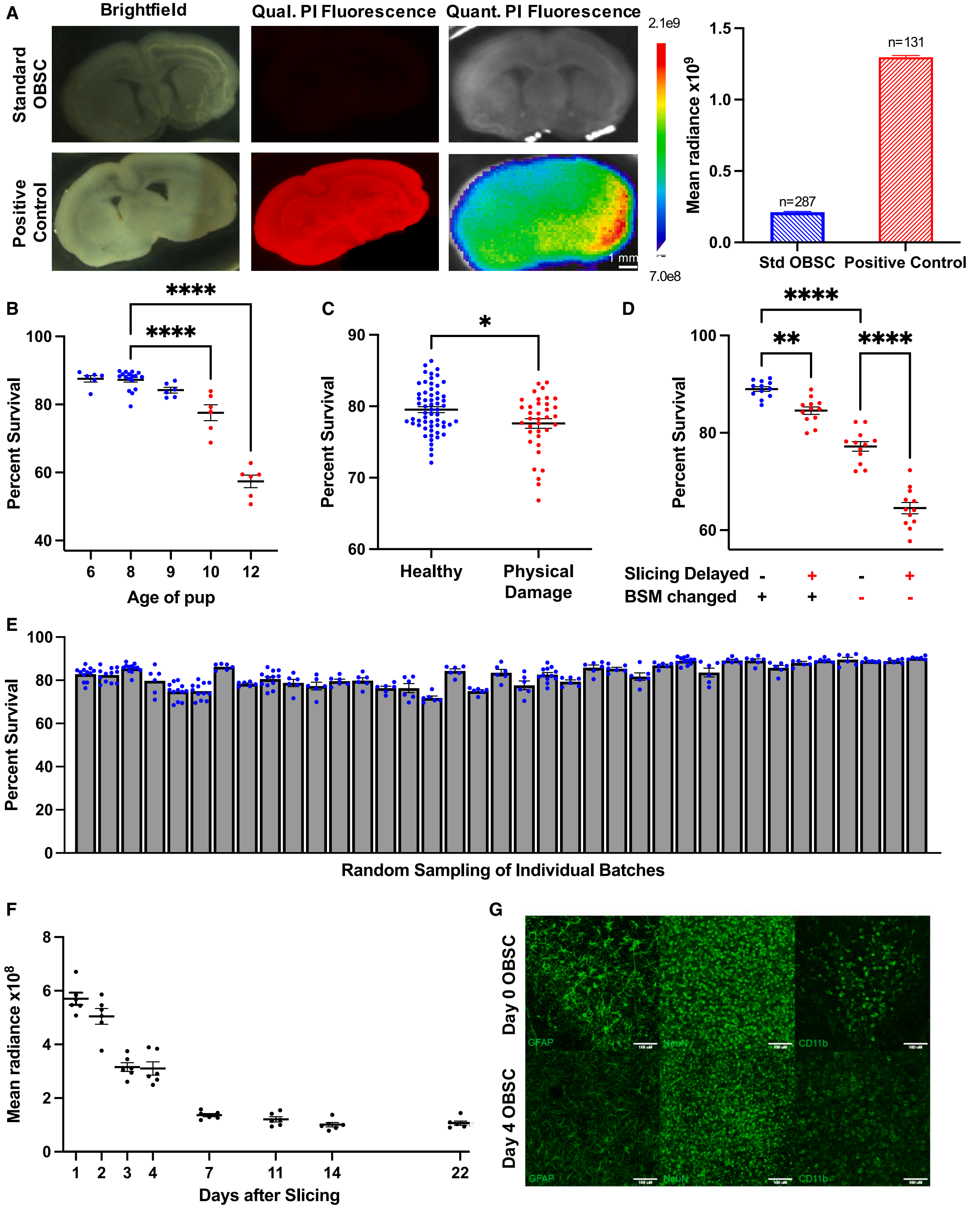

(legend on next page)

CellPress
OPEN ACCESS

with multiple drugs. The potential for PDMCs to guide personalized care has been demonstrated in several studies that show their ability to successfully predict antitumor response. Indeed, drug screening assays using PDMCs have successfully predicted antitumor responses in humans, demonstrating their potential.[9–13] Unfortunately, initiation time, cost, efficiency scales, and lack of similarity to the parent tumor limit applications of PDXs,[14] while patient-derived cell lines often lose the genetic and phenotypic heterogeneity of the parent tumor via cell selection during clonal expansion.[15] Some PDOs and PDEs can effectively capture the histological and mutational diversity of human cancers[16–20] but are most successfully generated from aggressive, high-grade tumors such as IDH-wild-type glioblastoma. Furthermore, generating these models from heterogeneous tumors also limits reproducibility among intra-tumor replicates. A platform that supports and rapidly engrafts both low- and high-grade brain tumor tissue, maintains genetic heterogeneity and resemblance to the parent tumor, and allows functional testing of approved and experimental therapeutics is still desperately needed.

In this study, we present our organotypic brain slice culture (OBSC) platform as a living tissue substrate capable of engrafting and testing uncultured patient brain tumor tissue for functional precision diagnosis. Our previous work has demonstrated that tumor lines grown on OBSCs replicate the tumor growth, invasion, and cellular interactions observed *in vivo*,[21] as well as how functional drug testing on OBSCs is supported by transcriptomic profiles.[22] We now use multiple additional assays and a multipoint algorithm to compare drug-induced killing of established tumor lines and uncultured patient tumor tissue engrafted onto OBSCs. Our standardized, optimized protocol has allowed successful engraftment and treatment of all tested patient tumor tissue samples across a variety of tumor types, grades, and patient ages. For each of nearly 150 drug-tumor-OBSC interactions, we calculated therapeutic windows by comparing both tumor kill and normal brain tissue (OBSC) toxicity, enabling normalized and direct drug-to-drug and tumor-to-tumor comparisons. Overall, these data highlight and validate our platform as a mature, clinically relevant technology.

RESULTS

Generation and characterization of living OBSCs

These studies use OBSCs generated from rat pups (RRID:MGI:5651135) as living tissue substrates to culture and treat tumor cell lines and uncultured patient brain tumor resection tissue. To generate this rapid, high-fidelity *ex vivo* testing platform, we first assessed OBSC quality and reproducibility, optimizing the process using quantitative fluorescence-based imaging methods. After piloting several assays, we adapted an established nuclear permeability assay using propidium iodide[23–30] (PI; catalog #P4170; Sigma-Aldrich) to quantify cell death within OBSCs. This method provided a large dynamic range to detect nuanced differences between healthy and unhealthy OBSCs, with higher fluorescence values indicating more dead cells (Figure 1A). OBSC quality was influenced by rat pup age at time of generation (Figure 1B); therefore, OBSCs were generated from eight-day-old pups for every experiment described in this study. We also found that optimal OBSCs were generated using improved methods for brain dissection and OBSC culture conditions (Figures 1C and 1D), establishing our robust, standardized procedure for OBSC generation. Following this optimization period, we reproducibly generated more than 6,000 living OBSCs across nearly 40 separate batches, randomly sampling and testing 6 OBSCs per batch for QC analysis (Figure 1E). All OBSCs with physical damage after slicing, such as a nick or a tear, were discarded, but this amounted to fewer than 2% of OBSCs over all experiments.

Just as brain tissue suffers acute injury after brain tumor resection, OBSCs suffer acute injury after slicing. Serial measurements of OBSC health showed an initially high PI signal that resolves over time, reaching baseline levels by day 7 (Figure 1F). In parallel, immunohistochemistry (IHC) analysis showed that astrocytes (GFAP; RRID:AB_305808) and macrophages/microglia (CD11b; RRID:AB_2650514) displayed reactive morphologies[31] immediately post-slicing (Figure 1G). This activation attenuates in astrocytes by day 4 but persists in macrophages/microglia, suggesting that the OBSCs may still contain dead cells and debris that are phagocytosed by the myeloid cells.[32,33] The morphology of neurons (NeuN; RRID:AB_2650514) remained unchanged between days 0 and 4. Together, these results describe a high-quality, well-controlled, reproducible method for generating and tracking the health of OBSCs. As such, we next focused on engrafting tumor cells onto these living tissue substrates.

Tumor modeling on OBSCs

Before attempting to move into uncultured patient tissue samples, we selected a diverse panel of human brain cancer lines (adult, pediatric, primary, and metastatic; defined in Figure 2 legend) to further identify the myriad capabilities of our OBSC

Figure 1. OBSC characterization, standardization, and quality control

(A) Left: bright-field and fluorescence images of standard healthy and dead (positive control; frozen in EtOH overnight) OBSCs, via PI stain. Right: average fluorescence from standard OBSCs (n = 287 biological replicates) and positive control OBSCs (n = 131 biological replicates) OBSCs.

(B) Effect of pup age on OBSC viability ($n \geq 6$ per day), analyzed using one-way ANOVA (****$p < 0.0001$).

(C) Viability comparison of healthy OBSCs vs. those damaged during dissection and/or slicing (n = 60 biological replicates for healthy, n = 36 biological replicates for damaged), analyzed using Welch's t test (p = 0.0172).

(D) Viability of OBSCs on the basis of brain slice media (BSM) quality and promptness during slicing (n = 12 biological replicates per group; slicing delay after dissection: 1 h), analyzed using one-way ANOVA (**p = 0.0052 and ****$p < 0.0001$).

(E) Representative batch-to-batch viability from $n \geq 6$ biological replicates randomly sampled OBSCs from each batch ($n \sim 150$ OBSCs per batch).

(F) PI signal from OBSCs measured on different days after slicing (n = 6 biological replicates).

(G) 10X immunofluorescent maximum intensity projection images of healthy OBSCs showing activity level of astrocytes (GFAP), neurons (NeuN), and microglia (CD11b) immediately after slicing and on day 4 (D4). All data, except where otherwise noted, were collected 4 days after slicing.

All data are shown as mean ± SEM.

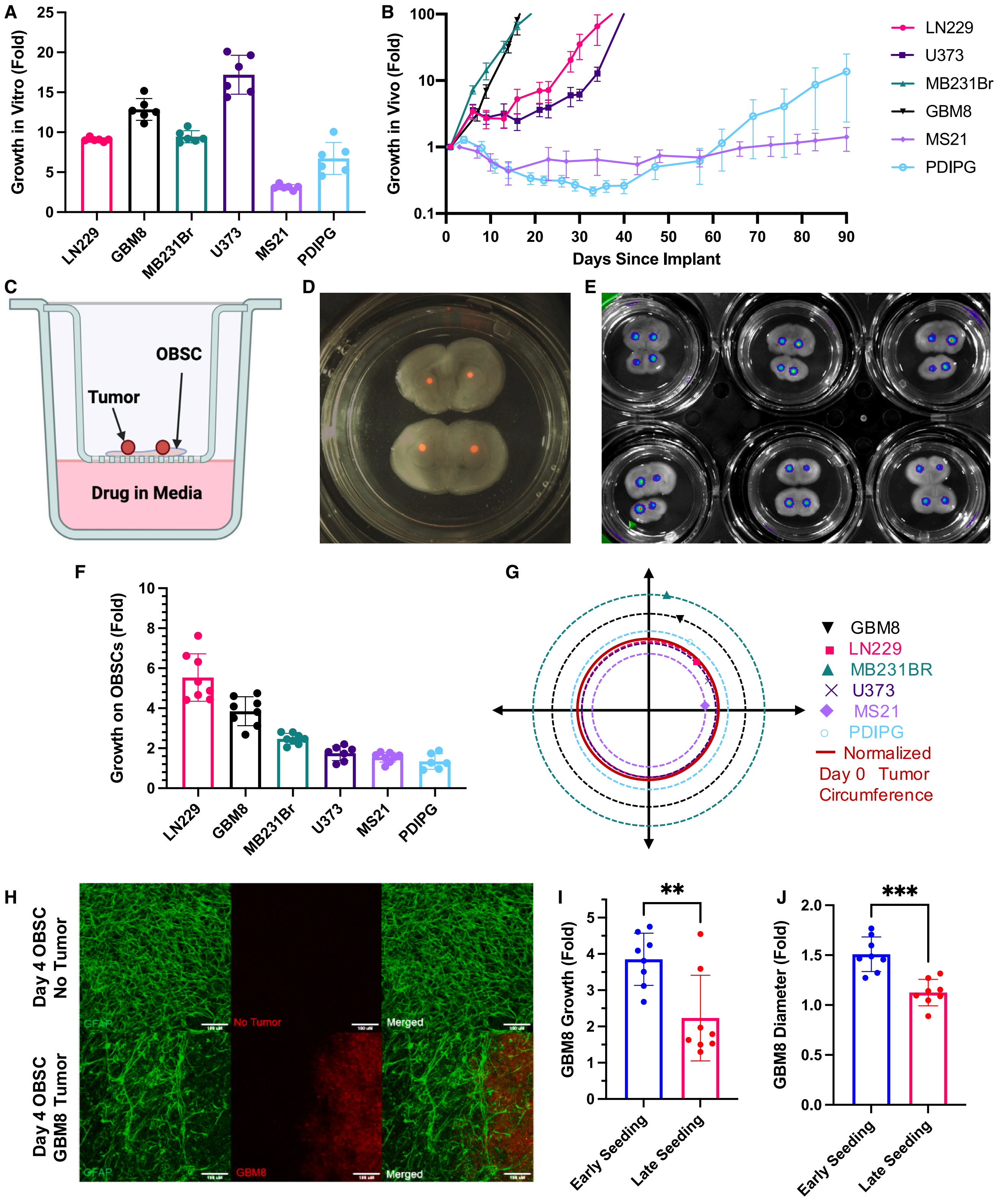

Figure 2. Tumor growth and interaction on OBSCs

(A) *In vitro* cell growth in 96 h. Fifteen hundred cells were seeded into 96-well plates and measured on days 0 and 4 via BLI; n = 6 technical replicates.

(B) *In vivo* tumor growth in mice following intracranial implantation of 250,000 tumor cells; n = 5 biological replicates.

(legend continued on next page)

assay and show how OBSCs provide a rapid, representative, and applicable niche for broad tumor engraftment and analysis. We first compared multiple tumor characteristics across *in vitro*, *in vivo*, and OBSC culture. All tumor lines were transduced with lentiviral vectors encoding mCherry-Firefly Luciferase (LV-mCh-FLuc) optical reporters, then the lines were seeded in 96-well culture plates, orthotopically xenografted into the brains of nude mice, or seeded atop the living OBSCs. We leveraged the bilateral symmetry of each coronally sectioned OBSC to seed tumor cells within the anatomically homogeneous thalamic region of each hemisphere. Serial imaging and quantitative analysis was performed to track growth, morphology, and rate of invasion.

Four high-passage tumor lines commonly used within the field, LN229, U373, MB231Br, and GBM8, grew rapidly *in vitro* (Figure 2A), *in vivo* via orthotopic implantation (Figure 2B), and on OBSCs (Figures 2C–2F). These tumor lines all expanded several-fold in just four days *in vitro* and on OBSCs and more than 100-fold *in vivo* within 40 days. The low-passage adult GBM line MS21 and pediatric DIPG line PDIPG displayed a different set of results. These two lines had been initially cultured via clonal expansion *in vitro* after human tumor biopsy, and both were able to be cultured/expanded *in vitro* between passage 3 and 20 during this work. Both lines grew several fold within four days *in vitro*, but neither reproducibly established *in vivo*, even after 90 days. On OBSCs, MS21 and PDIPG tumors both nearly doubled in size over four days, displaying how OBSCs can support growth of low-passage tumor cells not supported *in vivo*.

Tumors seeded on OBSCs rapidly establish among the endogenous astrocytes and microglia. Consistent with our previous data showing rapid invasion of only invasive tumor types along the corpus callosum within OBSCs,[21] we now observe tumor-specific trends in outward invasion or inward retraction of tumor cells on the thalamic region of OBSCs.[19] We found the invasive cell line GBM8, metastatic cell line MB231Br, and diffuse pediatric DIPG all invade radially outward on OBSCs, while other established lines that grow more densely *in vivo* retract inward (Figure 2G; qualitative examples in Figure S1A). Interestingly, microglia within OBSCs also reciprocally respond to the presence of tumor, maintaining activated morphologies and associating with nearby tumor cells, indicating a two-way interaction between the tumor and this *ex vivo* microenvironment (Figure 2H). Adding tumor cells to OBSCs on day 0 after slicing ("early seeding," our standard method), compared with day 7 ("late seeding") when microglial activation and PI signaling have attenuated, also leads to faster tumor growth and invasion (Figures 2I and 2J), consistent with *in vivo* tumor response.[34] Together, this suggests that OBSCs provide a niche for tumor growth and interrogation that is more appropriate and accurate than *in vitro* culture systems and less harsh than *in vivo* models.

Tumor treatment on OBSCs

We next used the panel of OBSC tumor models to investigate treatment responses within the platform. To assess the molecular specificity of drug-induced tumor killing on OBSCs, we used a CRISPR-modified variant of the U373 tumor line harboring a single-gene knockout in RAD18. Deletion of this DNA repair mediator should increase sensitivity and killing for agents that use this pathway. The deletion did not affect the growth of untreated wild-type or knockout cells, now termed U373WT and U373KO, both *in vitro* and on the OBSCs (Figure S1B).

We subjected all seven OBSC-engrafted tumors to treatment with a panel of approved CNS tumor therapeutics and external radiation therapy (XRad), as well as the experimental agent TR107 (Madera), a second-generation derivative of ONC201.[35,36] We tested six concentrations of each agent, assessing treatment response by quantitative bioluminescence imaging (BLI) to specifically measure tumor kill. Dose-response curves and half maximal inhibitory concentration (IC_{50}) values were then calculated for each agent (Figures 3A and S2; Table S1). If treatment elicited <50% death at the highest dose, we report "not reached" (NR).

By comparing IC_{50} values (Figure 3A), we found that 7 of 7 lines showed robust response to the proven agents carboplatin (catalog #C2538-100MG; Sigma-Aldrich) cisplatin (catalog #232120-50MG; Sigma-Aldrich), and lomustine (catalog #S1 840; Selleck Chemicals), with the PDX GBM cell line GBM8 showing markedly higher sensitivity. Several agents, including temozolomide (TMZ; catalog #T2577-100MG; Sigma-Aldrich), vincristine (catalog #V8388-1MG; Sigma-Aldrich), XRad, and gemcitabine (catalog #S1149; Sigma-Aldrich), failed to induce significant killing at even the highest tested doses in more than 5 of the 7 cell lines, resulting in designations of NR. Azacitidine (catalog #A2385-250MG; Sigma-Aldrich) and etoposide (catalog #E1383-100MG; Sigma-Aldrich) showed broad potency, inducing killing in 7 of 7 and 6 of 7 lines, respectively, at IC_{50} values in the 200–400 μM range.

When comparing U373WT vs. U373KO, we found that IC_{50} values were significantly decreased and killing significantly

(C) Graphic of cell line tumor engraftment onto OBSCs.

(D) Representative fluorescence image of 4 MB231Br tumor foci seeded into the thalamic region of two OBSCs within one well.

(E) Representative bioluminescence image depicting tumor seeding of 24 tumor foci onto 12 OBSCs in one six-well plate.

(F) Cell line growth in 96 h on OBSCs. Bioluminescence images were taken on days 0 and 4. Day 0 fluorescence images were used to normalize initial tumor size. n = 4 biological replicates.

(G) Migratory behavior from initial tumor engraftment site on OBSCs; t = 96 h. Circles larger than the normalized tumor circumference indicate outward migratory behavior while smaller circles indicate inward retraction. n = 4 biological replicates.

(H) 10X immunofluorescent maximum intensity projection images of astrocytes and tumor interaction: astrocytes stained by GFAP (green) without or in the presence of GBM8 tumor cells (red) 96 h after engraftment.

(I) Growth of GBM8 on OBSCs seeded early (day of slicing) or seeded late (7 days post-slicing). n = 8 biological replicates. Analyzed using an unpaired t test. p = 0.0051

(J) Diameter change of GBM8 on OBSCs seeded early or late. n = 8 biological replicates. Analyzed using an unpaired t test. p = 0.0001

All data are shown as mean ± SEM.

See also Figure S1.

A

	MB231Br	LN229	U373 WT	U373 KO	GBM8	MS21	PDIPG
Carboplatin	122	133	176	172	5.8	92	86
Cisplatin	125	92	42	42	0.45	25	17
Lomustine	1608	960	665	607	3.4	1554	688
Temozolomide	NR	NR	NR	1156	7.6	833	NR
Etoposide	100	NR	260	38	5.2	313	574
Gemcitabine	NR	NR	NR	0.9	0.34	NR	NR
Azacitidine	208	400	800	158	34	821	447
Radiation	NR	NR	NR	NR	3.8	NR	NR
Vincristine	NR	NR	NR	NR	0.05	4.6	NR
Trametinib	0.78	19	NR	NR	0.38	NR	NR
TR107	0.04	0.04	0.08	0.07	0.10	0.08	0.05

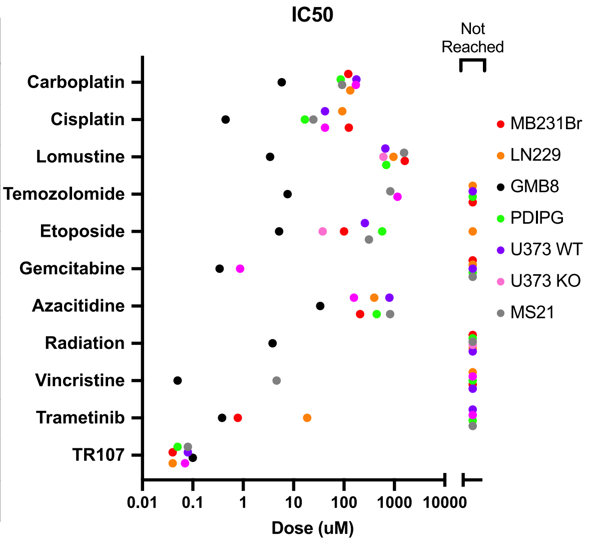

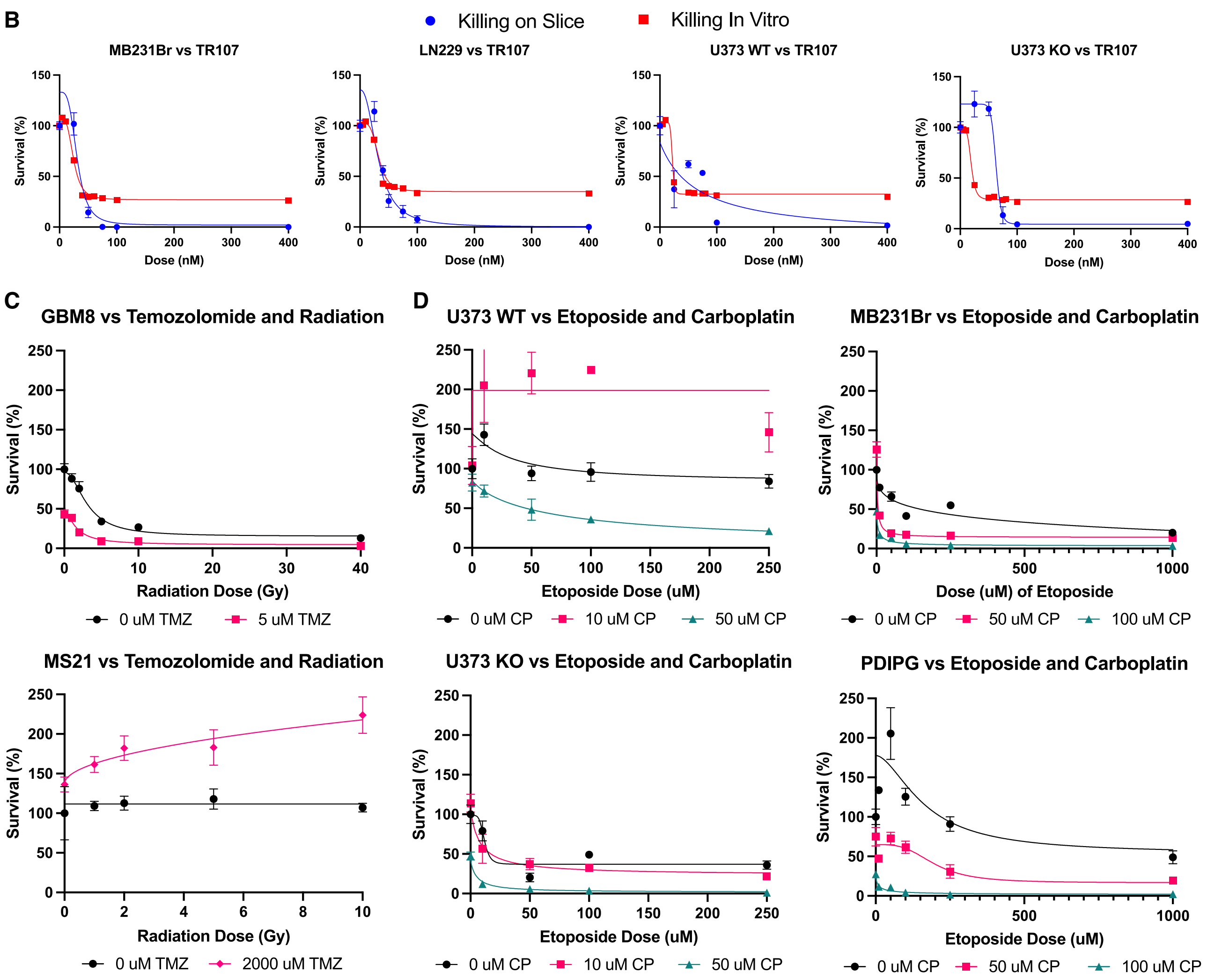

(legend on next page)

increased in U373KO following treatment with TMZ, azacitidine, gemcitabine, and etoposide. Interestingly, U373KO also showed increased resistance to radiation, possibly due to an upregulation of the nonhomologous end joining pathway.[37]

The experimental agent TR107 was the most potent therapeutic in our panel, inducing complete killing in 7 of 7 cell lines with IC_{50} values in the 0.04–0.1 μM range. Interestingly, potent but incomplete killing of tumor cells by TR107 is often observed in standard *in vitro* culture, both by us and in the literature (Figure 3B).[36] Because ClpP inhibitors such as TR107 and ONC201 can both directly act on tumor cells and indirectly increase tumor kill by acting on normal cells in the tumor microenvironment (TME),[38] the complete killing we observe when tumors are grown on OBSCs highlights a difference in functional killing patterns *in vitro* and on OBSCs that may be due to drug-tumor-OBSC interactions. Further defining the roles of OBSCs as active contributors to TME-based tumor kill is the aim of several future studies.

In the clinical setting, combination regimens are the mainstay of patient care. Following our investigation of single agent therapy, we next tested drug responses for clinically relevant therapeutic combinations on OBSCs (Figures 3C and 3D). Treatment of GBM8 across low doses of TMZ and varying doses of radiation resulted in additive killing with synergistic kill at high radiation doses, as indicated by the ZIP synergy score (Figure S1C),[39,40] while combining TMZ and radiation against MS21 revealed strong antagonism. When we compared treatment response of etoposide and carboplatin combination therapy against U373WT and U373KO, the addition of low-dose carboplatin to etoposide surprisingly produced an antagonistic effect in U373WT, which was reversed as higher doses of carboplatin led to enhanced killing by the combination regimen. This effect was not observed in U373KO, in which the elevated sensitivity to etoposide monotherapy led to robust killing and synergy from the combination regimen. Synergistic killing of MB231Br and PDIPG is also observed when treating these tumor lines with carboplatin and etoposide. These data highlight the ability of the OBSC platform to measure synergistic effects of combination therapies.

Advancing OBSCs as a tool for standardized drug testing

As therapeutics are developed for clinical use, assessment of (1) direct tumor killing and (2) toxicity to normal brain tissue both play central roles in defining efficacy. To provide a normalized comparison among drugs with varying potencies, we created a drug scoring system on the basis of OBSC functional testing. Leveraging our established quantitative imaging methods to measure tumor volumes and health of OBSCs, we assessed multiple parameters of drug activity, developed methods for normalized assessment that established a "therapeutic window," and collapsed data into a single ranked scoring system for multi-component comparison. This process was designed and optimized using our panel of brain tumor lines before moving into uncultured patient brain tumor tissue.

Numerous parameters, such as IC_{10}, IC_{50}, and area under the dose-response curve (AUC), are commonly used to define drug activity. Our analysis calculates 11 such parameters from tumor dose-response curves to determine multiple efficacy values for each agent against each cell line (Figures 4A and 4B; STAR Methods). Our ability to separately define values across tumor foci using BLI and normal brain tissue (OBSCs with no tumor added) using our predefined PI assay allowed us to measure both efficacy and off-target toxicity within many parameters and generate normalized therapeutic window ratios for each drug-tumor-OBSC interaction (Figures S3 and S4). Within these windows, values ranged from +1 to −1, where values approaching +1 signify increasing tumor kill relative to normal tissue toxicity, and values approaching −1 indicate agents where tumors remained highly viable while toxicity to the normal OBSC tissue was elevated. Incorporating OBSC toxicity in this way allowed comparison among drugs with less bias toward more potent compounds.

We first applied this approach to GBM8, U373KO, MS21, and LN229 treated with etoposide (Figure 4A). Multi-parameter analysis was driven by comparing each tumor's dose-response curve, with survival normalized to untreated tumor BLI, with the OBSC toxicity curve (Figure 4B). MS21 displays a consistent increase in growth in response to treatment, producing therapeutic windows close to −1 for many parameters. LN229 is moderately killed by etoposide treatment but still less than the OBSC itself, producing slightly negative therapeutic window values. U373KO and GBM8 all display incrementally more sensitivity to etoposide relative to the OBSC and thus each produce slightly more positive therapeutic windows across all parameters.

We next developed a multi-parametric algorithm to collapse the normalized parameters into a single score that simplifies comparison of each drug-tumor-OBSC interaction. This formula integrates more than 100 data points from tumor kill and OBSC toxicity dose-response curves across the 11 individually weighted parameters to derive an overall drug sensitivity score (DSS; described more fully in STAR Methods). The DSS was set to range from −100 to +100, where +100 describes maximal performing agents (greatest tumor kill, lowest toxicity) and −100 describes the lowest performing agents (poorest tumor kill,

Figure 3. Tumor killing on OBSCs

(A) Left: IC_{50} values calculated on the basis of linear interpolation of dose-response data on OBSCs. Cells were seeded on day 0 and dosed with therapeutics on day 1; survival was measured via bioluminescence on day 4. Concentrations of small molecule drugs are given in micromolar; XRad dose is given in grays. NR indicates the IC_{50} was not reached within the dose range. Right: graphical representation of IC_{50} values of all drugs vs. all tumor lines.

(B) Killing of MB231Br, LN229, U373WT, and U373KO by TR107 on OBSCs and *in vitro*. n = 6 biological replicate tumor foci per dose, 6 doses per cell line.

(C) Combination therapy of radiation and subsequent temozolomide against GBM8 and MS21.n = 4 biological replicate tumor foci per dose, 6 doses per cell line.

(D) Combination therapy of etoposide and carboplatin against U373WT, U373KO, MB231Br, and PDIPG.n = 4 biological replicate tumor foci per dose, 6 doses per cell line.

All data are shown as mean ± SEM.

See also Figures S1 and S2 and Table S1.

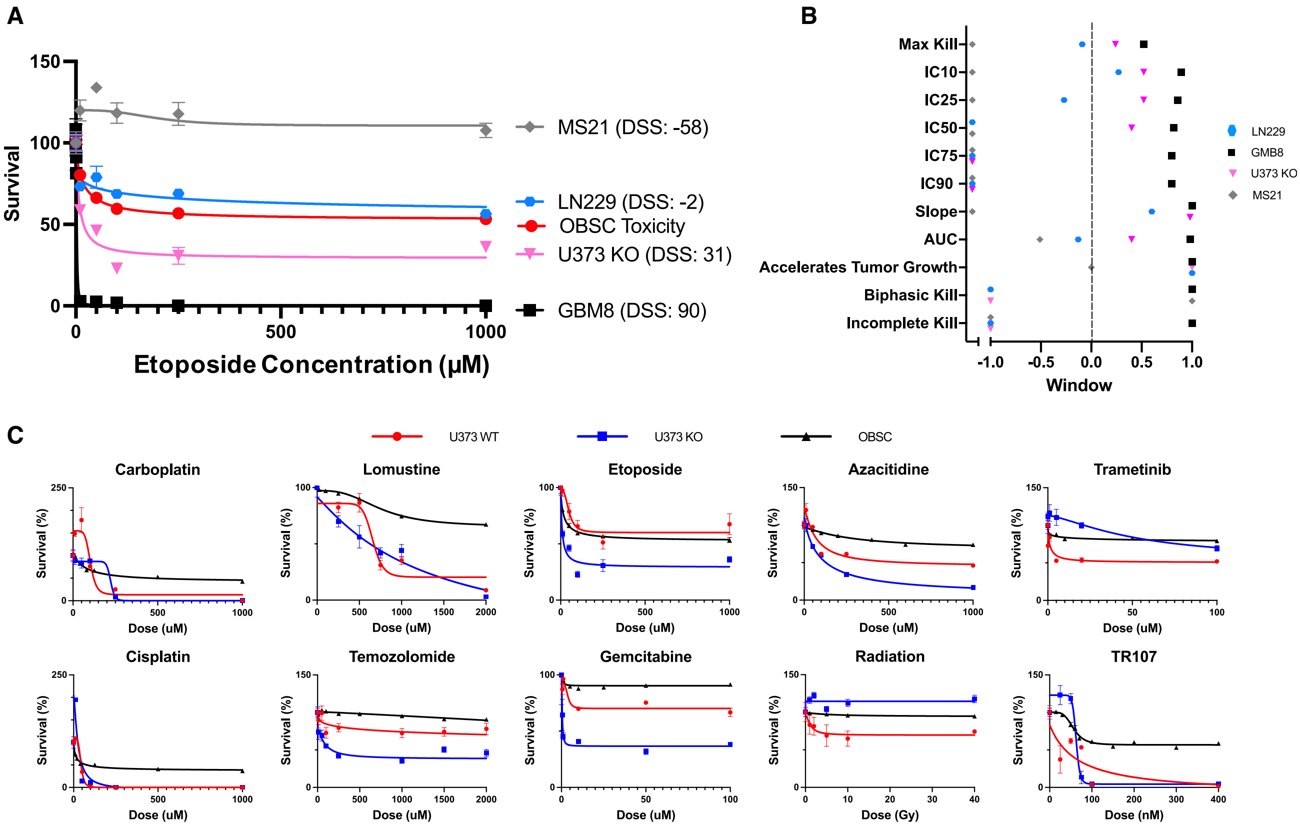

D

	MB231Br	LN229	U373 WT	U373 KO	GBM8	MS21	PDIPG
Carboplatin	43	75	39	64	93	53	70
Cisplatin	44	44	62	51	92	69	75
Lomustine	20	57	64	66	96	36	61
Temozolomide	-39	25	9	49	96	-62	27
Etoposide	15	-2	-13	31	90	-58	-2
Gemcitabine	-67	16	22	53	97	-25	21
Azacitidine	75	57	23	65	93	36	35
Radiation	-7	-39	28	-26	77	-6	22
Vincristine	-6	22	-63	-64	88	41	32
Trametinib	50	32	13	13	93	-32	-5
TR107	87	84	78	68	57	62	72

Color scale: 100, 90, 80, 70, 60, 50, 40, 30, 20, 10, 0, -10, -20, -30, -40, -50, -60, -70, -80, -90, -100

Figure 4. Drug sensitivity score algorithm and array

(A) Dose-response curves of GBM8, U373 KO, MS21, and LN229 on OBSCs t = 3 days after administration of etoposide. n = 4 biological replicate tumor foci per dose, 6 doses per cell line.

(B) Therapeutic windows across all DSS parameters for each treated tumor line from Figure 3A. See STAR Methods for further details.

(C) Dose-response curves of U373WT (red), U373KO (blue), and OBSC (black) against ten therapeutics.

(legend continued on next page)

highest toxicity). Figure 4A shows how DSS increases as tumor kill increases.

The value of our algorithm is strongest in *relative comparisons*, for example, when comparing tumor responses to several drugs or when comparing dose-response curves of varying shapes. We first applied the algorithm to define DSS for U373WT cells treated with different therapeutic agents (Figure 4C; red lines, U373WT; black lines, OBSC). As shown in Figures 4C and 4D, the established agents cisplatin and lomustine resulted in DSS of 62 and 64, respectively, near the top of the range for this cell line. TR107 received the highest DSS at 78, while etoposide scored near the bottom, with a DSS of −13. Although TR107 was extremely potent, displaying robust tumor kill at concentrations orders of magnitude lower than most other drugs, it also presented some toxicity to OBSCs within that range. By basing our DSS on therapeutic window, TR107 could be more fairly compared alongside less potent drugs such as TMZ or therapeutics such as XRad, which use different dosage units.

To investigate the ability of the algorithm to detect differences in single-gene alterations, we then compared drug response profiles of U373WT with those of the RAD18-knockout line U373KO (Figure 4C; blue lines, U373KO). As expected, we detected a marked difference in DSS for several therapies between the two cell lines, where RAD18 deletion led to increased sensitivity to TMZ, etoposide, and azacitidine and a corresponding increase in DSS. In comparing each pair of dose-response profiles against each therapeutic, some showed the most significant differences at low drug doses, while others showed greater curve separation at high doses, and still others displayed distinct killing profiles altogether, underscoring the importance of calculating a unified DSS which incorporates many aspects of each curve.

Last, we expanded our testing and generated DSS for 11 therapies across 8 different tumor types (Figure 4D), enabling broader comparisons and providing insight into drug-tumor activity. This allowed us to compare (1) the relative efficacy of a single agent across multiple tumor types (horizontal rows) and (2) the relative sensitivity of a single tumor to multiple agents (vertical columns). We found that GBM8 was the most sensitive tumor line to most therapeutics, with the majority of DSS scores near 90. TR107 was, on average, the most effective agent across tumor lines but, interestingly, was least effective against GBM8. TMZ and radiation, agents commonly used in clinical care for GBM, showed wide variability among tumors, with DSS scores ranging from −62 to 96 (TMZ) and −39 to 77 (radiation).

Using OBSCs as a diagnostic platform for uncultured patient brain tumor tissue

Although the field can choose from many models and assays when testing tumor lines, generating a viable, representative tumor model from uncultured patient tumor tissue has historically been a difficult task, especially in the field of brain cancer. Significant initial cell death is often observed when culturing cells *in vitro*, and *in vivo* PDX models require extensive lead times while yielding low rates of establishment.[41–43] Even brain tumor PDOs are most successfully established when modeling the most aggressive GBM subtypes.[20] To fill this need, we designed a method to prepare and engraft a diverse panel of living, uncultured patient brain tumor tissues onto OBSCs for rapid, functional drug screening and diagnosis. To increase flexibility in timing and assay selection, we also created and validated a method to cryopreserve patient tumor tissue while preserving the tumor's original genetic profile, persistence, and drug response on OBSCs. These methods (1) maximize tumor engraftment and viability independent of tumor grade or subtype, (2) limit cell loss/selection and genetic drift, (3) maintain a normal distribution of tumor per OBSC engraftment site to combat intra-tumoral heterogeneity, and (4) maximize the number of replicate tumor foci per mass of clinical tumor biopsy tissue.

We obtained fresh surgical biopsies from patients undergoing standard-of-care resection surgeries at University of North Carolina at Chapel Hill (UNC) hospitals following informed consent. Following cryopreservation and thaw according to our optimized protocol, the tissue was dissociated into a homogeneous near single-cell suspension, rapidly transduced with LV-mCh-FLuc, and seeded as tumor foci each containing a representative sample of ∼0.5 mg tissue onto OBSCs. To measure the ability of OBSCs to engraft patient brain tumor tissue, we first compared tumor tissue survival four days after seeding on (1) OBSCs in our culture insert setup (top left), (2) the culture insert membrane without OBSCs (bottom left), and (3) standard *in vitro* culture (right) via BLI (Figure 5A). Uncultured tumor resection tissue from three different patients bearing three different tumor types (grade I ganglioglioma [GG-I], grade 1 meningioma [MG-1], and a heterogeneous glioblastoma/meningioma tumor [GBM-MG]) showed consistent survival on OBSCs but not in other culture formats. Compared with their growth on OBSCs, GG-I, MG-I, and GBM-MG showed <1%, 29%, and <1% viability when cultured on the culture insert alone and <1%, 2%, and <1% viability, respectively, when cultured in standard *in vitro* culture plates.

We then applied this tumor seeding method to eight additional types of adult, pediatric, primary, and metastatic patient brain tumor resection tissue, 11 tumors in all (Figure 5B). As before, quantitative optical imaging revealed that every patient tumor we tested reproducibly persisted on OBSCs at t = 4 days after seeding, regardless of tumor type. As we sought to understand why OBSCs could provide such an accommodating niche for patient tumor engraftment, we again measured OBSC astrocyte activation around tumor tissue via GFAP. We found that the engrafted patient tumor tissue also induces activation of, and interaction with, OBSC-embedded astrocytes in a similar manner to the astrocyte activation observed after tumor line engraftment (Figures 5C and S5).

We then used uncultured tumor resection tissue from an NF2 mutation-driven grade II meningioma (MG-II) to investigate whether tumors engrafted onto OBSCs maintained the genetic

(D) DSS array for all cell lines against all drugs. DSSs from 0 to 100 signify increasing efficacy in tumor kill relative to OBSC toxicity, while scores from 0 to −100 describe scenarios in which tumors thrive more effectively than OBSCs for a given treatment.
All data are shown as mean ± SEM.
See also Figures S2–S4 and Table S1.

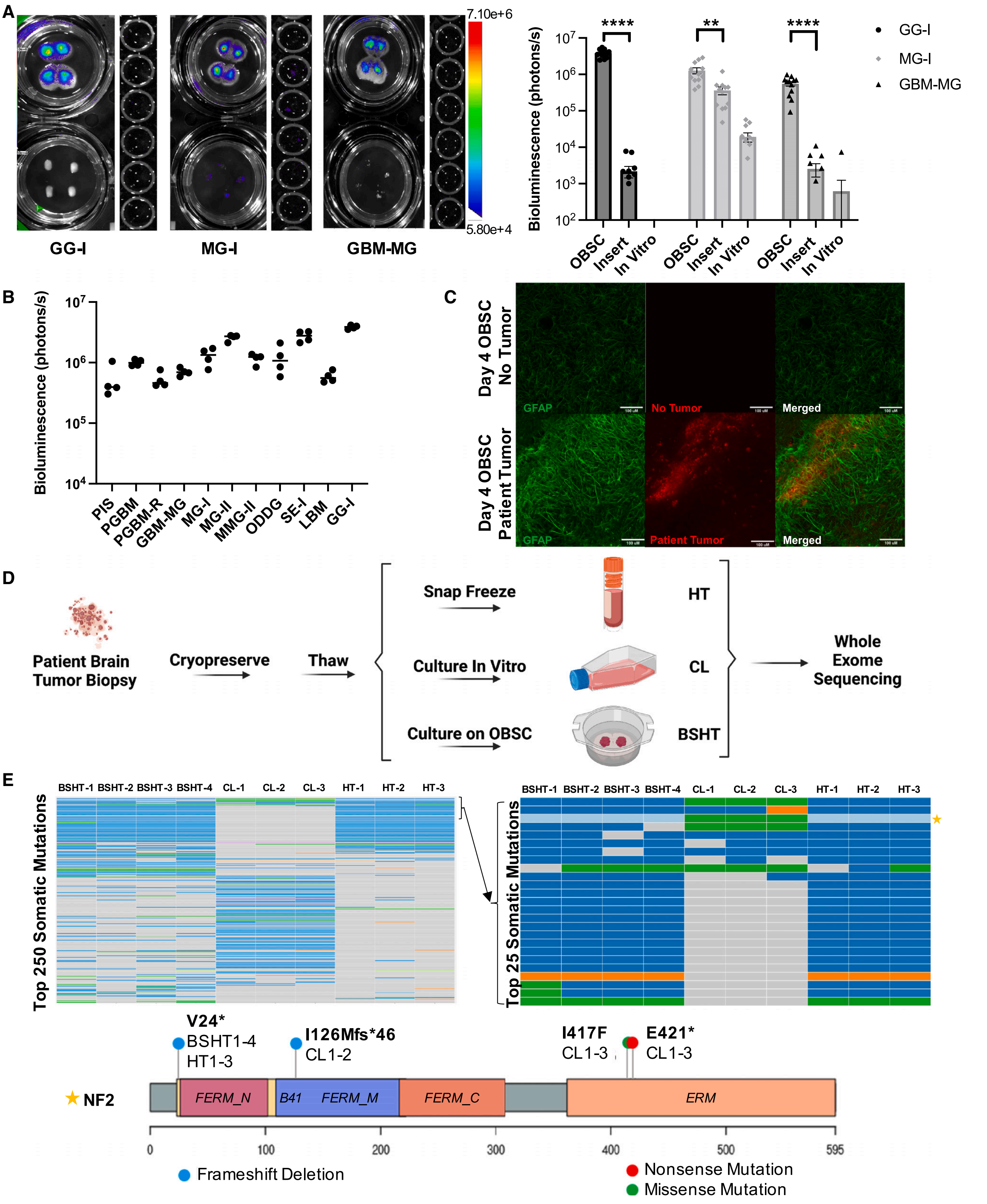

(legend on next page)

profile of the parent tumor. We prepared three groups of samples for whole-exome sequencing (WES) from the same pool of dissociated MG-II tissue (Figure 5D). Group HT is the original uncultured human tumor tissue (n = 3). Group CL is the parent tumor tissue expanded in standard *in vitro* cell culture until the minimum number of cells required for WES had grown. Because of initial cell loss and subsequent clonal expansion, this required six passages over the span of approximately one month (n = 3). Group BSHT is the uncultured tumor resection tissue engrafted onto OBSCs and subsequently dissected from the OBSCs at the conclusion of our standardized assay length (4 days; n = 4).

WES analysis showed that tumor tissue engrafted onto OBSCs maintained a significant genetic resemblance to the parent tumor, while tumor tissue expanded *in vitro* displayed a distinctly different profile (Figure 5E; left plot displays the top 250 most significantly mutated somatic genes [listed in Table S2], and right plot displays the top 25, derived from left plot). Furthermore, the mutational profiles of all four BSHT biological replicates were markedly similar, indicating that each OBSC-engrafted tumor indeed contained a representative sample of the original patient tumor. A closer look at the hallmark NF2 mutations existing within all samples revealed that while all samples from the original tumor (HT1–3) and the OBSC-engrafted tumor (BSHT1–4) maintain the frameshift deletion at V24, this mutation was lost in all samples expanded *in vitro* (CL1–3) and replaced by mutations in other areas. Together, these data suggest that the rapid assay design and tumor-accommodating niche of our OBSC platform enables effective maintenance of the original patient tumor profile.

Consistency in seeding and viability is essential for accurate and reproducible results when assessing therapeutic agents. To investigate consistency in initial seeding, we labeled patient tumor tissue with a fluorescent dye and compared the initial sizes of 24 tumor foci at 1 h post-seeding (two foci per OBSC, two OBSCs per well). We found that well-to-well reproducibility was high and yielded no statistically significant differences (Figure 6A). We found similar consistency in longitudinal tumor persistence, with no statistical differences in viability (via BLI) among unique samples on days 4, 6, and 8 after seeding (Figure 6B; n = 6 foci).

OBSCs can also support the persistence of cryopreserved and thawed patient tumor tissue. Our method of cryo-storage, thaw, and engraftment onto OBSCs generates reproducible persistence (Figure 6C) and response to therapeutic agents (Figure 6D) compared with fresh tumor tissue from the same patient tumor, significantly enhancing the versatility and flexibility of this platform.

Patient tumor sensitivities assessed by DSS

Viable brain tumor resection tissue is often limited, leading in part to the dearth of functional testing strategies for brain cancers. Although functional diagnostic platforms for other solid tumors generally test many therapeutics and require a large amount of tissue, we purposefully limit the number of screened therapeutics to those already being considered by attending clinicians. We have set up our workflow to accept any amount of available tumor tissue and screen even a small number of drugs in order to facilitate clinical decisions among top therapeutic options. For each tissue we acquire, our clinical team helps curate our panel of relevant therapeutics and determine which drugs to test on each tumor on the basis of tumor type, mutational status, and amount of available tissue. By working with clinicians in this way, we focus on comparisons that could, in future studies, directly guide care for each patient in real time.

Uncultured brain tumor resection tissue from ten patients was seeded onto OBSCs and treated with various therapeutics to generate DSSs (Figures 7A and S6; Table S3). Figure 7A compares patient DSSs alongside DSSs for all established tumor lines (data repeated from Figure 4D). Trends across every DSS for all tumors (Figures 7B and S7) show that our DSS algorithm can calculate scores throughout the entire scoring range: patient tumors are generally more sensitive to treatment than the cell culture-selected established lines in our panel, save for the ubiquitously sensitive GBM8 tumor line, but there is still a wide distribution in patient tumor sensitivities across all drugs and tumors.

Table S4 describes all tumor types and reported mutational statuses of patient tumor tissue used in this study. Multitudes of co-occurring mutations appear frequently in this set of tumors, presenting difficulty in making treatment decisions on the basis of mutational status alone. We can begin to correlate our results to this clinical data by comparing mutations, treatments, and outcomes in individual patients with DSSs calculated from treating matched live patient tissue on OBSCs.

Correlating OBSC-derived patient sensitivities to clinical data

We next investigated the correlation between treatment responses predicted by the OBSC platform and the true clinical response using this small cohort of patient biopsies. To this end, we collected clinical pathologic and genomic data on patient biopsies under our open protocol. Here, we report correlative data on specimens that received additional treatment after surgical resection. The OBSC platform and DSSs did not inform any prospective patient treatment in this work.

Figure 5. Patient tumor tissue on OBSCs

(A) Qualitative (left) and quantitative (right) BLI of three different patient brain tumor tissues cultured *in vitro*, on culture insert and on OBSC 4 days after seeding (t test, $^{**}p < 0.001$ and $^{****}p < 0.0001$; n = 12 biological replicates).

(B) Bioluminescence of each patient tumor t = 4 days after seeding on OBSCs (n = 4 biological replicates per tumor; full tumor names in Table S4).

(C) 10X immunofluorescent maximum intensity projection images of astrocytes and patient tumor interaction: astrocytes stained by GFAP (green) without or in the presence of PGBM patient tumor (red via mCherry expression) 96h after engraftment. See also Figure S5.

(D) Schematic of experimental design for DNA sequencing of patient tumor tissue (MG-II).

(E) Heatmap of the top 250 most significantly mutated somatic genes showing maintenance of HT profile in BSHT but not in CL, (top left), top 25 most significantly mutated genes (top right; starred row, NF2), and mutations within the NF2 gene on chromosome 22 in HT, CL, and BSHT (bottom). BSHT, n = 4 biological replicates; HT, n = 3 technical replicates; CL, n = 3 biological replicates.

All data are shown as mean ± SEM.

See also Figure S5 and Tables S2 and S4.

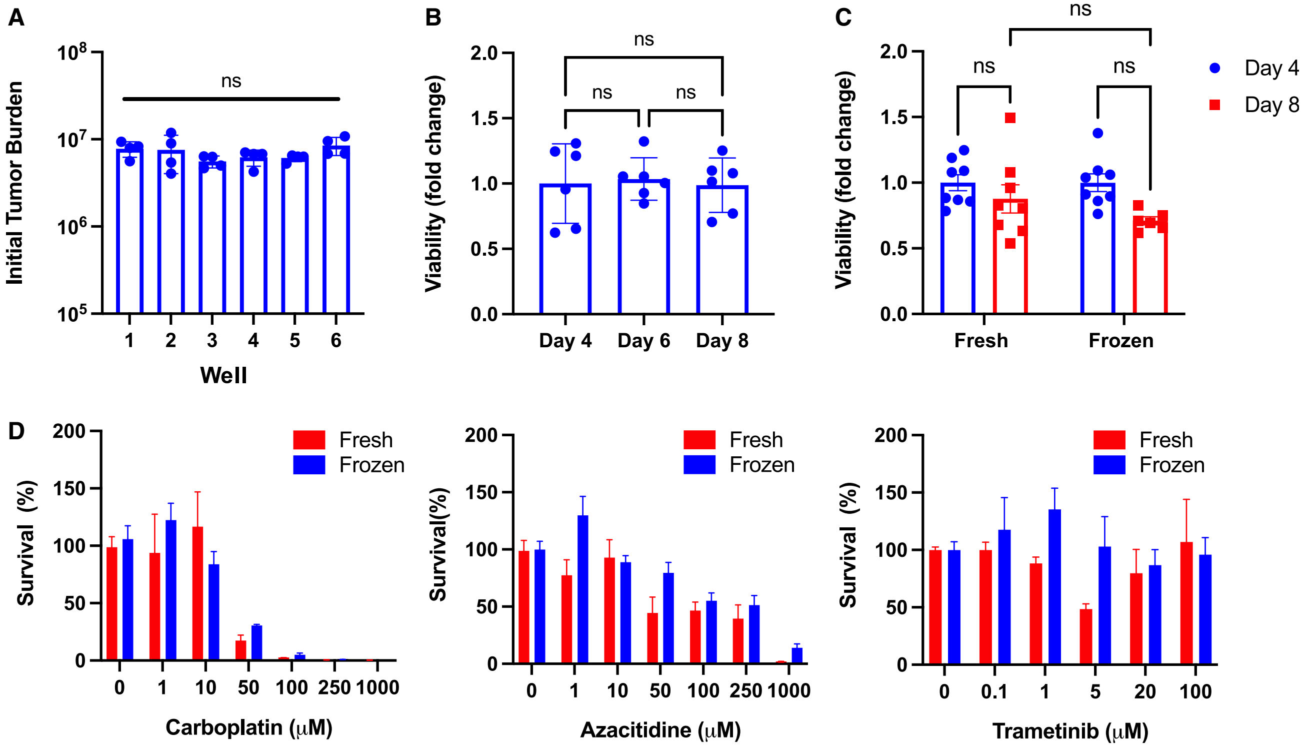

Figure 6. Patient tumor tissue on OBSCs

(A) Reproducibility in PGBM-R patient tumor seeding via Cell Tracker fluorescence at t = 1 h after seeding on OBSCs. Analyzed using one-way ANOVA. p = 0.3484

(B) Reproducibility in persistence of unique tumor foci from MMG-II patient tumor 4, 6, and 8 days after seeding; n = 6 biological replicates. Analyzed using one-way ANOVA. p = 0.9345

(C) Reproducibility in persistence of unique tumor foci from fresh and cryopreserved/thawed (frozen) tissue from the same GG-I patient tumor sample 4 and 8 days after seeding on OBSCs; n = 6 biological replicates. Analyzed using two-way ANOVA. p = 0.2881

(D) Reproducibility in survival of fresh and cryopreserved/thawed (frozen) tumor tissue from the same GG-I patient tumor sample t = 3 days after treatment with carboplatin, azacitidine and trametinib; n = 4 biological replicate foci per dose.

All data are shown as mean ± SEM.

A pediatric patient underwent subtotal tumor resection for a rim-enhancing, centrally necrotic, left temporal mass. Tumor PGBM underwent extensive histopathological and genomic analysis for clinical purposes and demonstrated diffuse and high-grade glioblastoma histology, *IDH1* mutation (*IDH1* c.395G>A [p.Arg132His]), *MGMT* promoter methylation profile, *TERT* promoter wild-type, and absence of 1p/19q codeletion. IHC also demonstrated "patchy" *BRAF V600E* positivity, although DNA analysis of the tumor did not demonstrate any *BRAF V600E* alteration. DNA analysis for hotspot mutations also demonstrated *CDK4* amplification, *PAX5* V129M, *PIK3CA* H1047R, and *TP53* R273C (Table S4).

Despite the many identified histopathological and genomic events in this tumor, the optimal choice on the basis of driver predictions and drug efficacy is simply not well understood within the current paradigm of precision oncology. Several potential targets for precision medicine are theoretically conceivable, including TMZ to target *MGMT* methylation,[44] IDH inhibitor to target *IDH1* mutation,[45] CDK4/6 inhibitor to target *CDK4* amplification,[46] PIK3CA inhibitor to target *PIK3CA* mutation-induced *AKT* activation,[47] and BRAF and MEK inhibitors to target the patchy BRAF V600E positivity on IHC,[48] but it is difficult to predict which will be most effective.

Bolstered by the clinical prediction that this *MGMT*-methylated tumor should respond well to TMZ, the patient underwent standard-of-care treatment with concurrent XRad and TMZ following subtotal resection. The patient exhibited minimal response to therapy and the tumor rapidly progressed. Although this outcome was not predicted by MGMT methylation status, it was consistent with OBSC data: our DSSs suggested that TMZ (DSS = 26) and XRad (DSS = 16) would show limited efficacy against this tumor (Figure 7A).

The discrepant IHC and molecular results surrounding the BRAF V600E status of this tumor may have suggested a possible role for BRAF or MEK inhibition. Although this patient never received a MEK inhibitor to target MAPK pathway upregulation associated with the possible *BRAF V600E* mutation, trametinib vs. PGBM on OBSCs killed about one-third of the tumor at the highest dose (DSS = 16), correlating slightly better with the negative DNA mutation analysis of the tumor rather than the patchy positive IHC, although the term "patchy" is certainly qualitative.

A

	MB231Br	LN229	U373 WT	U373 KO	GBM8	MS21	DIPG	PIS	PGBM	PGBM-R	GBM-MG	MG-I	MG-II	MMG-II	ODDG	SE-I	LBM
Carboplatin	43	75	39	64	93	53	70	88		90	88	89	70	23	78	80	84
Cisplatin	44	44	62	51	92	69	75	73				78	80	73			78
Lomustine	20	57	64	66	96	36	61	78		76	81	43	70		53		59
Temozolomide	-39	25	9	49	96	-62	27	20	26	17	63	67	-50		-72	20	34
Etoposide	15	-2	-13	31	90	-58	-2	80			48						72
Gemcitabine	-67	16	22	53	97	-25	21	48					50	61			
Azacitidine	75	57	23	65	93	36	35	45	-24	72	85	66	79	84	73	71	
Radiation	-7	-39	28	-26	77	-6	22	37	16	25	48	33	22	53	30		
Vincristine	-6	22	-63	-64	88	41	32	51					48	48	85		
Trametinib	50	32	13	13	93	-32	-5	-9	16	74			54	48			
TR107	87	84	78	68	57	62	72	70	88	86			74	77			

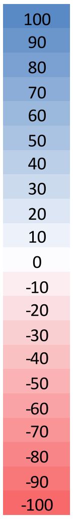

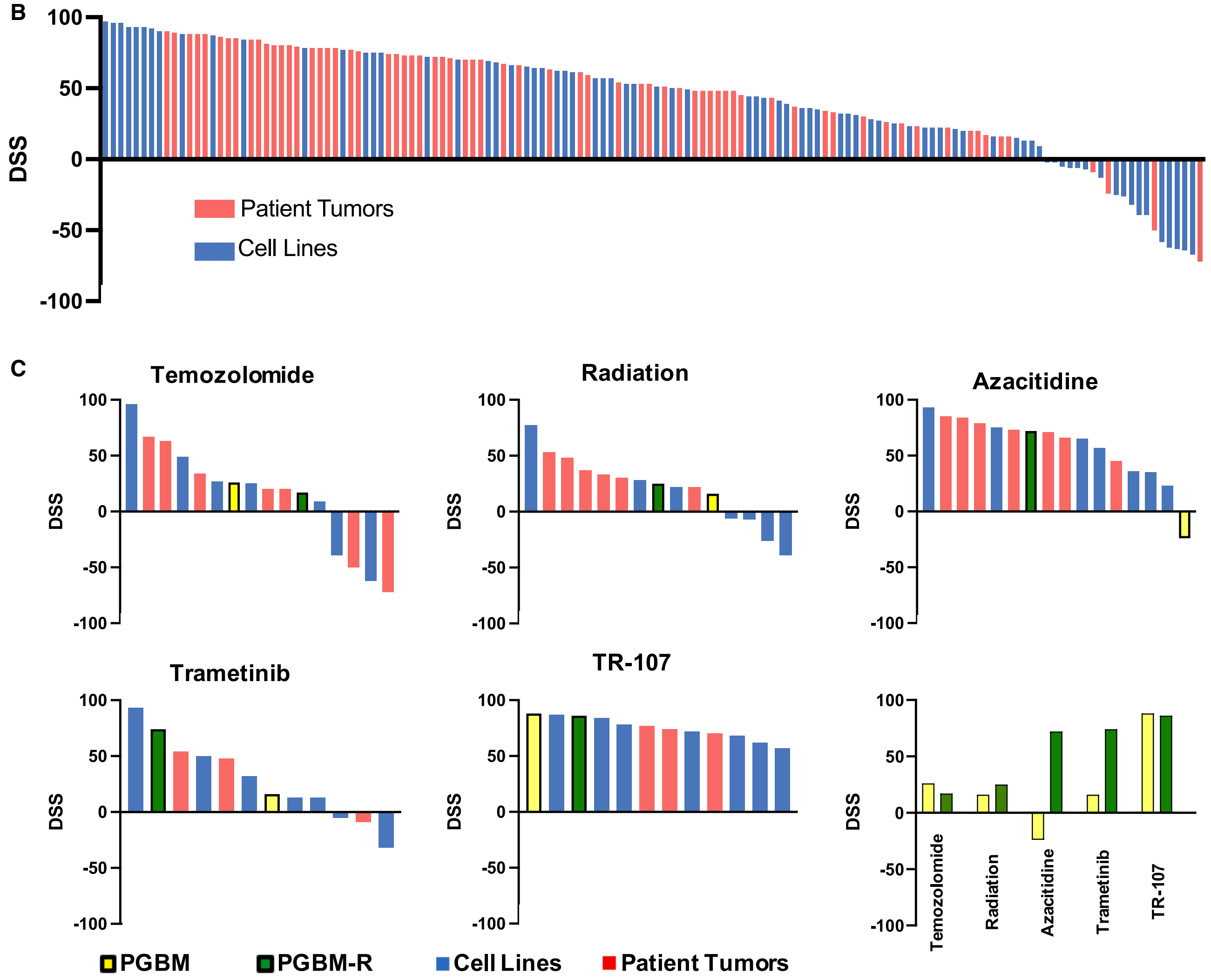

(legend on next page)

This patient underwent re-resection of the recurrent tumor, yielding PGBM-R. The patient resumed TMZ treatment but again showed minimal response to therapy. This again aligns with OBSC results for PGBM-R, which suggested that TMZ (DSS = 17) would continue to be ineffective. Clinical genomic profiling reported that the MGMT promoter was no longer methylated in the post-treatment tumor, suggesting development of a possible resistance mechanism.[49]

Genomic profiles are known to change upon tumor recurrence, and these differences can lead to changes in drug sensitivities as well. Figure 7C shows DSS profiles of PGBM (yellow) and PGBM-R (green) relative to all other tested tumor lines (blue) and patient tissues (red). Although sensitivity to TMZ and XRad did not significantly change upon tumor recurrence, DSSs for azacitidine and trametinib increased by 96 and 58 points, respectively, in the recurrent tumor. This functional data could be integral in developing updated treatment plans for recurrent tumors which fail first-line treatment.

Analysis of other tumor samples revealed additional associations between DSS scoring, clinical outcomes, and genomic analysis. Patient tumor PIS received XRad, etoposide, and carboplatin/etoposide/ifosfamide (ICE) treatment (DSS via OBSC for XRad = 37, etoposide = 80, carboplatin = 88) and has not experienced recurrence. Patient tumor MMG-II received XRad (DSS = 53; top among patient tumors) and continues to be in relapse-free remission. Other low-grade tumors we tested may have been surgically cured, although our ability to functionally screen these tumors for drug sensitivities could provide additional data to inform more comprehensive future diagnoses which include functional data.

DISCUSSION

Although significant advancement in CNS tumor diagnostics have been made in the past decades, with a meaningful move away from grouping tumors exclusively on the basis of their appearance under light microscopy and toward integrated histopathological and molecular diagnoses, there remains a critical functional gap in the diagnostic schema: how will the tumor respond to therapy? Even within contemporary, well-defined, uniform tumor subtypes, there exist profoundly stubborn non-uniform clinical outcomes.[50,51] The data presented here describes our OBSC-based technology as a functional diagnostic tool that can begin to fill this critical gap.

Here, we (1) further characterize and standardize our OBSC platform and assay, (2) describe how OBSCs can model tumor lines that are not readily established *in vivo*, (3) treat a diverse set of brain cancer cell lines with a panel of therapeutics, (4) introduce a multi-parametric algorithm to analyze dose-response data of both tumor kill and normal brain tissue toxicity, (5) translate the technology toward the clinic via testing on a wide variety of uncultured patient brain tumor specimens, and (6) begin to validate this translational approach by identifying areas of association between patient tumor DSS and clinical outcomes/genomic data.

Our DSS array generated from established and low-passage tumor lines helps substantiate our findings in patient tumor tissue. This array acts as a reference dataset, defining therapeutic window and DSS values for responders and non-responders while probing responses to known mutational profiles and predicted sensitivities. MB231Br and GBM8 are both tumor cell lines that express upregulation or dependency on the MAPK pathway—MB231Br via the BRAF V600E mutation[52] and GBM8 via the PTEN deletion[53]—suggesting sensitivity to the MEK inhibitor trametinib. Indeed, these demonstrated the two highest DSS vs. trametinib among cell lines. Furthermore, the invariably high DSS in the GBM8 cell line correlates with other experiments using this cell line that have demonstrated a broad sensitivity to many drugs.[53,54] Furthermore, the RAD18 knockout U373 cell line, a line with an inability to activate the DNA damage response after alkylator chemotherapy exposure, resulted in expected changes in therapeutic sensitivity to TMZ compared with the wild-type line.[55] The increased sensitivity to TMZ exhibited by U373KO on OBSCs reflects what has previously been shown (Yang, submitted) and furthers the reliability of OBSCs to capture nuanced differences in sensitivity and reflect these differences in a DSS. Together, these data indicate the ability of OBSCs to preserve targetable mutations in tumor cells while also affording the requisite DSS sensitivity to differentiate between those lines that are more and less sensitive.

As evidenced by our work with TR107, this OBSC assay and algorithm can also evaluate the efficacy of experimental therapeutics. TR107 averaged the highest DSS among all treated tumors and obtained the highest individual DSS in 5 of 12 tumors, suggesting that this drug may have potential for further pre-clinical development in these indications. Furthermore, the distinct tumor killing patterns of TR107 on OBSCs compared with *in vitro* data suggest that OBSCs may model drug interactions within the TME as well. Other experimental therapeutics may be screened in this way to determine relative efficacy vs. standard of care, help interrogate resistance mechanisms to a specific therapeutic or class of drugs as they are being developed, or even use this platform to screen patients for eligibility on a clinical trial. Future work will test the clinical feasibility of OBSCs as

Figure 7. Combined DSS

(A) Combined DSS array for all cell lines and patient tumor tissue against all drugs. Tumor line data from Figure 4 are repeated here for comparison. DSSs from 0 to 100 signify increasing efficacy in tumor kill relative to slice toxicity, while scores from 0 to −100 describe scenarios in which tumors thrive more effectively than OBSCs for a given treatment. Gray boxes indicate drugs that were not tested against a certain tumor.

(B) Waterfall plot of all 145 DSSs presented in (A). DSSs from established tumor lines are represented as blue lines; DSSs from patient tumor tissues are represented as red lines.

(C) Waterfall plots depicting relative sensitivities to individual therapeutics. All tumors treated with each therapeutic are included; individual tumor names are shown in Figure S5. Bottom right: head-to-head comparison of DSSs from drugs used to treat PGBM and PGBM-R. Yellow lines, DSS for PGBM; green lines, DSS for PGBM-R; blue lines, DSS for established tumor lines; red lines, DSS for patient tumor tissue.

See also Figures S6 and S7 and Tables S2–S4.

living tissue substrates in a trial that is currently being initiated at UNC.

Limitations of the study

It is important to acknowledge the aims of our platform in light of its constraints. We are not seeking to merely answer the question, Will the drug kill the patient's tumor? Even the most robust models, by definition, will provide incomplete answers here. Instead, our assay frames its questions in *relative* terms: Will a drug kill the patient's tumor more effectively *than other drugs* or more completely in one tumor *than in other tumors*? Our therapeutic window calculations allow us to base our analysis in these relative comparisons, enabling us to help physicians make decisions among top options. For example, when measured alone, a DSS of 70 might be seen as a "good" score because it is close to 100; however, comparisons alongside other DSSs are required to provide sufficient context. Was this DSS of 70 the lowest of all drugs tested against a particularly sensitive tumor? Was the DSS of 70 the highest score for this drug across a panel of high-grade gliomas? As more tumors are tested using this platform, the cumulative data will more and more effectively inform the physician on which drug(s) may be most, or least, effective against a patient's tumor, but not which dose to use or how much of the patient's residual tumor we expect that dose to kill.

Furthermore, we acknowledge that our OBSC model does not contain functional vasculature or elements of the blood-brain barrier (BBB) and does not score or rank drugs on the basis of their BBB permeability. Because BBB properties can fluctuate among tumor types and will be compromised after tumor resection, we have elected not to include these considerations within our algorithm. Future work could focus on pairing our platform alongside models of BBB drug permeability, especially to compare experimental or off-label therapeutics.

Conclusions

In summary, our OBSC platform fills an unmet gap in the diagnosis and treatment of CNS tumors: functional precision diagnosis. This assay and algorithm treat the patient's own tumor cells with both approved and experimental therapeutics and provide a normalized and simply summarized DSS output, which we aim to use to help guide treatment and evaluate new therapies.

STAR★METHODS

Detailed methods are provided in the online version of this paper and include the following:

- KEY RESOURCES TABLE
- RESOURCE AVAILABILITY
  - Lead contact
  - Materials availability
  - Data and code availability
- EXPERIMENTAL MODEL AND SUBJECT DETAILS
  - Animal ethics statement
  - Human subjects ethics statement
  - Cell lines
  - Generating OBSCs
  - In vivo tumor studies
- METHOD DETAILS
  - Chemicals
  - Lentiviral vectors
  - Propidium iodide assay
  - Immunohistochemistry
  - In vitro studies
  - Tumor growth on OBSCs
  - Drug screening on OBSCs
  - Combination therapy
  - Preparing patient tissue for engraftment onto OBSCs
  - Preparing cell line from patient tissue
  - DNASeq
- QUANTIFICATION AND STATISTICAL ANALYSIS
  - Calculating drug sensitivity scores
  - Calculating ZIP synergy scores and plots
  - Statistical analysis

SUPPLEMENTAL INFORMATION

Supplemental information can be found online at https://doi.org/10.1016/j.xcrm.2023.101042.

ACKNOWLEDGMENTS

We would like to thank all of the patients and their families who generously donated tissue and the Tissue Procurement Facility at UNC for obtaining the tissue. We would also like to thank the Biomedical Research Imaging Center at UNC; Ian's Friends Foundation for their cell lines; H. Wakimoto and T. Yoneda for gifting us with GBM8 and MDA-MB231-Br; and Madera Therapeutics for their generous gift of TR107. This work was supported by National Center for Advancing Translational Sciences grant U01TR003715, the Eshleman Institute for Innovation, and Accelerate Brain Cancer Cure. Some figures were created using BioRender.com.

AUTHOR CONTRIBUTIONS

Conceptualization, B.M., X.Z., S.H., and A.B.S.; Methodology, B.M., X.Z., N.B., M.T., and A.B.S.; Validation, A.A. and R.D.; Formal Analysis, K.K. and A. Buckley; Investigation, B.M., X.Z., N.B., A.A., M.T., R.D., A.V., and V.L.; Resources, Y.Y., C.Q., N.C., C.V., and L.M.G.; Data Curation, B.M. and X.Z.; Writing – Original Draft, B.M., X.Z., and A.B.S.; Writing – Review & Editing, B.M., X.Z., D.K., S.R., A. Baldwin, S.H., and A.B.S.; Visualization, B.M., X.Z., A.A., M.T., R.D., K.K., and A.B.S.; Supervision, C.Q., Y.R., D.K., C.V., D.L.C., S.R., Y.J., L.M.G., D.D., S.F., A. Baldwin, S.H., and A.B.S.; Project Administration, A.B.S.; Funding Acquisition, S.F., A. Baldwin, S.H., and A.B.S.

DECLARATION OF INTERESTS

B.M., X.Z., N.B., A.V., A. Baldwin, S.H., and A.B.S. have submitted a provisional patent application on the basis of the work presented. All other authors declare no competing interest.

INCLUSION AND DIVERSITY

We worked to ensure gender balance in the recruitment of human subjects. We worked to ensure ethnic or other types of diversity in the recruitment of human subjects. We worked to ensure that the study questionnaires were prepared in an inclusive way. We worked to ensure sex balance in the selection of non-human subjects. We worked to ensure diversity in experimental samples through the selection of the cell lines. One or more of the authors of this paper self-identifies as an underrepresented ethnic minority in their field of research or within their geographical location. One or more of the authors of this paper self-identifies as a gender minority in their field of research. One or more of the authors

of this paper self-identifies as a member of the LGBTQIA+ community. One or more of the authors of this paper received support from a program designed to increase minority representation in their field of research.

Received: December 16, 2022
Revised: March 8, 2023
Accepted: April 19, 2023
Published: May 15, 2023

REFERENCES

1. Louis, D.N., Wesseling, P., Aldape, K., Brat, D.J., Capper, D., Cree, I.A., Eberhart, C., Figarella-Branger, D., Fouladi, M., Fuller, G.N., et al. (2020). cIMPACT-NOW update 6: new entity and diagnostic principle recommendations of the cIMPACT-Utrecht meeting on future CNS tumor classification and grading. Brain Pathol. *30*, 844–856. https://doi.org/10.1111/bpa.12832.
2. Haslam, A., Kim, M.S., and Prasad, V. (2021). Updated estimates of eligibility for and response to genome-targeted oncology drugs among US cancer patients, 2006-2020. Ann. Oncol. *32*, 926–932. https://doi.org/10.1016/j.annonc.2021.04.003.
3. Blakely, C.M., Watkins, T.B.K., Wu, W., Gini, B., Chabon, J.J., McCoach, C.E., McGranahan, N., Wilson, G.A., Birkbak, N.J., Olivas, V.R., et al. (2017). Evolution and clinical impact of co-occurring genetic alterations in advanced-stage EGFR-mutant lung cancers. Nat. Genet. *49*, 1693–1704. https://doi.org/10.1038/ng.3990.
4. de Bruin, E.C., McGranahan, N., Mitter, R., Salm, M., Wedge, D.C., Yates, L., Jamal-Hanjani, M., Shafi, S., Murugaesu, N., Rowan, A.J., et al. (2014). Spatial and temporal diversity in genomic instability processes defines lung cancer evolution. Science *346*, 251–256. https://doi.org/10.1126/science.1253462.
5. Sulkowski, P.L., Corso, C.D., Robinson, N.D., Scanlon, S.E., Purshouse, K.R., Bai, H., Liu, Y., Sundaram, R.K., Hegan, D.C., Fons, N.R., et al. (2017). 2-Hydroxyglutarate produced by neomorphic IDH mutations suppresses homologous recombination and induces PARP inhibitor sensitivity. Sci. Transl. Med. *9*, eaal2463. https://doi.org/10.1126/scitranslmed.aal2463.
6. Chandarlapaty, S., Sawai, A., Scaltriti, M., Rodrik-Outmezguine, V., Grbovic-Huezo, O., Serra, V., Majumder, P.K., Baselga, J., and Rosen, N. (2011). AKT inhibition relieves feedback suppression of receptor tyrosine kinase expression and activity. Cancer Cell *19*, 58–71. https://doi.org/10.1016/j.ccr.2010.10.031.
7. Manchado, E., Weissmueller, S., Morris, J.P., Chen, C.-C., Wullenkord, R., Lujambio, A., de Stanchina, E., Poirier, J.T., Gainor, J.F., Corcoran, R.B., et al. (2016). A combinatorial strategy for treating KRAS-mutant lung cancer. Nature *534*, 647–651. https://doi.org/10.1038/nature18600.
8. Poulikakos, P.I., Zhang, C., Bollag, G., Shokat, K.M., and Rosen, N. (2010). RAF inhibitors transactivate RAF dimers and ERK signalling in cells with wild-type BRAF. Nature *464*, 427–430. https://doi.org/10.1038/nature08902.
9. Barretina, J., Caponigro, G., Stransky, N., Venkatesan, K., Margolin, A.A., Kim, S., Wilson, C.J., Lehár, J., Kryukov, G. v, Sonkin, D., et al. (2012). The Cancer Cell Line Encyclopedia enables predictive modelling of anticancer drug sensitivity. Nature *483*, 603–607. https://doi.org/10.1038/nature11003.
10. Gao, H., Korn, J.M., Ferretti, S., Monahan, J.E., Wang, Y., Singh, M., Zhang, C., Schnell, C., Yang, G., Zhang, Y., et al. (2015). High-throughput screening using patient-derived tumor xenografts to predict clinical trial drug response. Nat. Med. *21*, 1318–1325. https://doi.org/10.1038/nm.3954.
11. Lu, M., Zessin, A.S., Glover, W., and Hsu, D.S. (2017). Activation of the mTOR pathway by oxaliplatin in the treatment of colorectal cancer liver metastasis. PLoS One *12*, e0169439. https://doi.org/10.1371/journal.pone.0169439.
12. Majumder, B., Baraneedharan, U., Thiyagarajan, S., Radhakrishnan, P., Narasimhan, H., Dhandapani, M., Brijwani, N., Pinto, D.D., Prasath, A., Shanthappa, B.U., et al. (2015). Predicting clinical response to anticancer drugs using an ex vivo platform that captures tumour heterogeneity. Nat. Commun. *6*, 6169. https://doi.org/10.1038/ncomms7169.
13. Vlachogiannis, G., Hedayat, S., Vatsiou, A., Jamin, Y., Fernández-Mateos, J., Khan, K., Lampis, A., Eason, K., Huntingford, I., Burke, R., et al. (2018). Patient-derived organoids model treatment response of metastatic gastrointestinal cancers. Science *359*, 920–926. https://doi.org/10.1126/science.aao2774.
14. Drost, J., and Clevers, H. (2018). Organoids in cancer research. Nat. Rev. Cancer *18*, 407–418. https://doi.org/10.1038/s41568-018-0007-6.
15. Namekawa, T., Ikeda, K., Horie-Inoue, K., and Inoue, S. (2019). Application of prostate cancer models for preclinical study: advantages and limitations of cell lines, patient-derived xenografts, and three-dimensional culture of patient-derived cells. Cells *8*, 74. https://doi.org/10.3390/cells8010074.
16. Jenkins, R.W., Aref, A.R., Lizotte, P.H., Ivanova, E., Stinson, S., Zhou, C.W., Bowden, M., Deng, J., Liu, H., Miao, D., et al. (2018). Ex vivo profiling of PD-1 blockade using organotypic tumor spheroids. Cancer Discov. *8*, 196–215. https://doi.org/10.1158/2159-8290.CD-17-0833.
17. Neal, J.T., Li, X., Zhu, J., Giangarra, V., Grzeskowiak, C.L., Ju, J., Liu, I.H., Chiou, S.-H., Salahudeen, A.A., Smith, A.R., et al. (2018). Organoid modeling of the tumor immune microenvironment. Cell *175*, 1972–1988.e16. https://doi.org/10.1016/j.cell.2018.11.021.
18. Yuki, K., Cheng, N., Nakano, M., and Kuo, C.J. (2020). Organoid models of tumor immunology. Trends Immunol. *41*, 652–664. https://doi.org/10.1016/j.it.2020.06.010.
19. Nickl, V., Schulz, E., Salvador, E., Trautmann, L., Diener, L., Kessler, A.F., Monoranu, C.M., Dehghani, F., Ernestus, R.-I., Löhr, M., and Hagemann, C. (2022). Glioblastoma-derived three-dimensional ex vivo models to evaluate effects and efficacy of tumor treating fields (TTFields). Cancers *14*, 5177. https://doi.org/10.3390/cancers14215177.
20. Jacob, F., Salinas, R.D., Zhang, D.Y., Nguyen, P.T.T., Schnoll, J.G., Wong, S.Z.H., Thokala, R., Sheikh, S., Saxena, D., Prokop, S., et al. (2020). A patient-derived glioblastoma organoid model and biobank recapitulates inter- and intra-tumoral heterogeneity. Cell *180*, 188–204.e22. https://doi.org/10.1016/j.cell.2019.11.036.
21. Satterlee, A.B., Dunn, D.E., Lo, D.C., Khagi, S., and Hingtgen, S. (2019). Tumoricidal stem cell therapy enables killing in novel hybrid models of heterogeneous glioblastoma. Neuro Oncol. *21*, 1552–1564. https://doi.org/10.1093/neuonc/noz138.
22. Satterlee, A.B., Dunn, D.E., Valdivia, A., Malawsky, D., Buckley, A., Gershon, T., Floyd, S., and Hingtgen, S. (2022). Spatiotemporal analysis of induced neural stem cell therapy to overcome advanced glioblastoma recurrence. Mol. Ther. Oncolytics *26*, 49–62. https://doi.org/10.1016/j.omto.2022.06.004.
23. Lossi, L., Alasia, S., Salio, C., and Merighi, A. (2009). Cell death and proliferation in acute slices and organotypic cultures of mammalian CNS. Prog. Neurobiol. *88*, 221–245. https://doi.org/10.1016/j.pneurobio.2009.01.002.
24. Hezel, M., Ebrahimi, F., Koch, M., and Dehghani, F. (2012). Propidium iodide staining: a new application in fluorescence microscopy for analysis of cytoarchitecture in adult and developing rodent brain. Micron *43*, 1031–1038. https://doi.org/10.1016/j.micron.2012.04.006.
25. Zimmer, J., Kristensen, B.W., Jakobsen, B., and Noraberg, J. (2000). Excitatory amino acid neurotoxicity and modulation of glutamate receptor expression in organotypic brain slice cultures. Amino Acids *19*, 7–21. https://doi.org/10.1007/s007260070029.
26. Romero-Leguizamón, C.R., Elnagar, M.R., Kristiansen, U., and Kohlmeier, K.A. (2019). Increasing cellular lifespan with a flow system in organotypic culture of the Laterodorsal Tegmentum (LDT). Sci. Rep. *9*, 1486. https://doi.org/10.1038/s41598-018-37606-3.
27. Buskila, Y., Breen, P.P., Tapson, J., van Schaik, A., Barton, M., and Morley, J.W. (2014). Extending the viability of acute brain slices. Sci. Rep. *4*, 5309. https://doi.org/10.1038/srep05309.
28. Happ, D.F., and Tasker, R.A. (2016). A method for objectively quantifying propidium iodide exclusion in organotypic hippocampal slice cultures.

CellPress
OPEN ACCESS

J. Neurosci. Methods *269*, 1–5. https://doi.org/10.1016/j.jneumeth.2016.05.006.

29. Noraberg, J. (2004). Organotypic brain slice cultures: an efficient and reliable method for neurotoxicological screening and mechanistic studies. Altern. Lab. Anim. *32*, 329–337. https://doi.org/10.1177/026119290403200403.
30. Suh, E.C., Jung, Y.J., Kim, Y.A., Park, E.M., and Lee, K.E. (2008). Aβ25–35 induces presynaptic changes in organotypic hippocampal slice cultures. Neurotoxicology *29*, 691–699. https://doi.org/10.1016/j.neuro.2008.04.001.
31. Escartin, C., Galea, E., Lakatos, A., O'Callaghan, J.P., Petzold, G.C., Serrano-Pozo, A., Steinhäuser, C., Volterra, A., Carmignoto, G., Agarwal, A., et al. (2021). Reactive astrocyte nomenclature, definitions, and future directions. Nat. Neurosci. *24*, 312–325. https://doi.org/10.1038/s41593-020-00783-4.
32. Fu, R., Shen, Q., Xu, P., Luo, J.J., and Tang, Y. (2014). Phagocytosis of microglia in the central nervous system diseases. Mol. Neurobiol. *49*, 1422–1434. https://doi.org/10.1007/s12035-013-8620-6.
33. Katayama, T., Kobayashi, H., Okamura, T., Yamasaki-Katayama, Y., Kibayashi, T., Kimura, H., Ohsawa, K., Kohsaka, S., and Minami, M. (2012). Accumulating microglia phagocytose injured neurons in hippocampal slice cultures: involvement of p38 MAP kinase. PLoS One *7*, e40813. https://doi.org/10.1371/journal.pone.0040813.
34. Wu, S.-Y., and Watabe, K. (2017). The roles of microglia macrophages in tumor progression of brain cancer and metastatic disease. Front. Biosci. *22*, 1805–1829. https://doi.org/10.2741/4573.
35. Bonner, E.R., Waszak, S.M., Grotzer, M.A., Mueller, S., and Nazarian, J. (2021). Mechanisms of imipridones in targeting mitochondrial metabolism in cancer cells. Neuro Oncol. *23*, 542–556. https://doi.org/10.1093/neuonc/noaa283.
36. Fennell, E.M.J., Aponte-Collazo, L.J., Wynn, J.D., Drizyte-Miller, K., Leung, E., Greer, Y.E., Graves, P.R., Iwanowicz, A.A., Ashamalla, H., Holmuhamedov, E., et al. (2022). Characterization of TR-107, a novel chemical activator of the human mitochondrial protease ClpP. Pharmacol. Res. Perspect. *10*, e00993. https://doi.org/10.1002/prp2.993.
37. Huang, J., Huen, M.S.Y., Kim, H., Leung, C.C.Y., Glover, J.N.M., Yu, X., and Chen, J. (2009). RAD18 transmits DNA damage signalling to elicit homologous recombination repair. Nat. Cell Biol. *11*, 592–603. https://doi.org/10.1038/ncb1865.
38. Prabhu, V.V., Morrow, S., Rahman Kawakibi, A., Zhou, L., Ralff, M., Ray, J., Jhaveri, A., Ferrarini, I., Lee, Y., Parker, C., et al. (2020). ONC201 and imipridones: anti-cancer compounds with clinical efficacy. Neoplasia *22*, 725–744. https://doi.org/10.1016/j.neo.2020.09.005.
39. Yadav, B., Wennerberg, K., Aittokallio, T., and Tang, J. (2015). Searching for drug synergy in complex dose-response landscapes using an interaction potency model. Comput. Struct. Biotechnol. J. *13*, 504–513. https://doi.org/10.1016/j.csbj.2015.09.001.
40. Zheng, S., Wang, W., Aldahdooh, J., Malyutina, A., Shadbahr, T., Tanoli, Z., Pessia, A., and Tang, J. (2022). SynergyFinder plus: toward better interpretation and annotation of drug combination screening datasets. Dev. Reprod. Biol. *20*, 587–596. https://doi.org/10.1016/j.gpb.2022.01.004.
41. Chokshi, C.R., Savage, N., Venugopal, C., and Singh, S.K. (2020). A patient-derived xenograft model of glioblastoma. STAR Protoc. *1*, 100179. https://doi.org/10.1016/j.xpro.2020.100179.
42. Kerstetter-Fogle, A.E., Harris, P.L.R., Brady-Kalnay, S.M., and Sloan, A.E. (2020). Generation of glioblastoma patient-derived intracranial xenografts for preclinical studies. Int. J. Mol. Sci. *21*, 5113. https://doi.org/10.3390/ijms21145113.
43. William, D., Mullins, C.S., Schneider, B., Orthmann, A., Lamp, N., Krohn, M., Hoffmann, A., Classen, C.-F., and Linnebacher, M. (2017). Optimized creation of glioblastoma patient derived xenografts for use in preclinical studies. J. Transl. Med. *15*, 27. https://doi.org/10.1186/s12967-017-1128-5.
44. Alnahhas, I., Alsawas, M., Rayi, A., Palmer, J.D., Raval, R., Ong, S., Giglio, P., Murad, M.H., and Puduvalli, V. (2020). Characterizing benefit from temozolomide in MGMT promoter unmethylated and methylated glioblastoma: a systematic review and meta-analysis. Neurooncol. Adv. *2*, vdaa082. https://doi.org/10.1093/noajnl/vdaa082.
45. DiNardo, C.D., Stein, E.M., de Botton, S., Roboz, G.J., Altman, J.K., Mims, A.S., Swords, R., Collins, R.H., Mannis, G.N., Pollyea, D.A., et al. (2018). Durable remissions with ivosidenib in IDH1-mutated relapsed or refractory AML. N. Engl. J. Med. *378*, 2386–2398. https://doi.org/10.1056/NEJMoa1716984.
46. Tien, A.-C., Li, J., Bao, X., Derogatis, A., Kim, S., Mehta, S., and Sanai, N. (2019). A phase 0 trial of ribociclib in recurrent glioblastoma patients incorporating a tumor pharmacodynamic- and pharmacokinetic-guided expansion cohort. Clin. Cancer Res. *25*, 5777–5786. https://doi.org/10.1158/1078-0432.CCR-19-0133.
47. Janku, F., Wheler, J.J., Naing, A., Falchook, G.S., Hong, D.S., Stepanek, V.M., Fu, S., Piha-Paul, S.A., Lee, J.J., Luthra, R., et al. (2013). PIK3CA mutation H1047R is associated with response to PI3K/AKT/mTOR signaling pathway inhibitors in early-phase clinical trials. Cancer Res. *73*, 276–284. https://doi.org/10.1158/0008-5472.CAN-12-1726.
48. Fusco, M.J., Piña, Y., Macaulay, R.J., Sahebjam, S., Forsyth, P.A., Peguero, E., and Walko, C.M. (2021). Durable progression-free survival with the Use of BRAF and MEK inhibitors in four cases with BRAF V600E-mutated gliomas. Cancer Control *28*, 10732748211040012. https://doi.org/10.1177/10732748211040013.
49. Feldheim, J., Kessler, A.F., Feldheim, J.J., Schulz, E., Wend, D., Lazaridis, L., Kleinschnitz, C., Glas, M., Ernestus, R.-I., Brandner, S., et al. (2022). Effects of long-term temozolomide treatment on glioblastoma and astrocytoma WHO grade 4 stem-like cells. Int. J. Mol. Sci. *23*, 5238. https://doi.org/10.3390/ijms23095238.
50. Hegi, M.E., Diserens, A.-C., Gorlia, T., Hamou, M.-F., de Tribolet, N., Weller, M., Kros, J.M., Hainfellner, J.A., Mason, W., Mariani, L., et al. (2005). MGMT gene silencing and benefit from temozolomide in glioblastoma. N. Engl. J. Med. *352*, 997–1003. https://doi.org/10.1056/NEJMoa043331.
51. Nobre, L., Zapotocky, M., Ramaswamy, V., Ryall, S., Bennett, J., Alderete, D., Balaguer Guill, J., Baroni, L., Bartels, U., Bavle, A., et al. (2020). Outcomes of BRAF V600E pediatric gliomas treated with targeted BRAF inhibition. JCO Precis. Oncol. *4*, 561–571. https://doi.org/10.1200/PO.19.00298.
52. Hollestelle, A., Nagel, J.H.A., Smid, M., Lam, S., Elstrodt, F., Wasielewski, M., Ng, S.S., French, P.J., Peeters, J.K., Rozendaal, M.J., et al. (2010). Distinct gene mutation profiles among luminal-type and basal-type breast cancer cell lines. Breast Cancer Res. Treat. *121*, 53–64. https://doi.org/10.1007/s10549-009-0460-8.
53. Piccirillo, S.G.M., Colman, S., Potter, N.E., van Delft, F.W., Lillis, S., Carnicer, M.-J., Kearney, L., Watts, C., and Greaves, M. (2015). Genetic and functional diversity of propagating cells in glioblastoma. Stem Cell Rep. *4*, 7–15. https://doi.org/10.1016/j.stemcr.2014.11.003.
54. Pavel, A.B., and Korolev, K.S. (2017). Genetic load makes cancer cells more sensitive to common drugs: evidence from Cancer Cell Line Encyclopedia. Sci. Rep. *7*, 1938. https://doi.org/10.1038/s41598-017-02178-1.
55. Hanisch, D., Krumm, A., Diehl, T., Stork, C.M., Dejung, M., Butter, F., Kim, E., Brenner, W., Fritz, G., Hofmann, T.G., and Roos, W.P. (2022). Class I HDAC overexpression promotes temozolomide resistance in glioma cells by regulating RAD18 expression. Cell Death Dis. *13*, 293. https://doi.org/10.1038/s41419-022-04751-7.
56. Wakimoto, H., Kesari, S., Farrell, C.J., Curry, W.T., Zaupa, C., Aghi, M., Kuroda, T., Stemmer-Rachamimov, A., Shah, K., Liu, T.-C., et al. (2009). Human glioblastoma-derived cancer stem cells: establishment of invasive glioma models and treatment with oncolytic herpes simplex virus vectors. Cancer Res. *69*, 3472–3481. https://doi.org/10.1158/0008-5472.CAN-08-3886.

STAR★METHODS

KEY RESOURCES TABLE

REAGENT or RESOURCE	SOURCE	IDENTIFIER
**Antibodies**		
Glial Fibiralliry Acidic Protein antibody	Abcam	Cat# ab7260; RRID:AB_305808
Anti-NeuN antibody [EPR12763]	Abcam	Cat# ab177487; RRID:AB_2532109
Anti-CD11b antibody [EPR1344]	Abcam	Cat# ab133357; RRID:AB_2650514
Goat Anti-Rabbit IgG (H + L) Antibody, Alexa Fluor 488 Conjugated	Molecular Probes	Cat# A-11008; RRID:AB_143165
Anti-NeuN antibody [EPR12763]	Abcam	Cat# ab177487; RRID:AB_2532109
Anti-CD11b antibody [EPR1344]	Abcam	Cat# ab133357; RRID:AB_2650514
Goat Anti-Rabbit IgG (H + L) Antibody, Alexa Fluor 488 Conjugated	Molecular Probes	Cat# A-11008; RRID:AB_143165
**Bacterial and virus strains**		
FLuc-mCh-puro Lentivirus non-concentrated (Lentivirus expressing Firefly Luciferase-mCherry, puromycin selection)	Duke Viral Vector Core	N/A
**Biological samples**		
Human glioblastoma samples	University of North Carolina Chapel Hill	N/A
**Chemicals, peptides, and recombinant proteins**		
Carboplatin	Sigma-Aldrich	Cat# C2538-100MG
Temozolomide, ≥98% (HPLC)	Sigma-Aldrich	Cat# T2577-100MG
Etoposide, synthetic, ≥98%, powder	Sigma-Aldrich	Cat# E1383-100MG
5-Azacytidine, ≥98% (HPLC)	Sigma-Aldrich	Cat# A2385-250MG
Vincristine sulfate, meets USP testing specifications	Sigma-Aldrich	Cat #V8388-1MG
cis-Platin ≥98%, Calbiochem®	EMD Millipore	Cat# 232120-50MG
Lomustine	Selleck Chemicals	Cat# S1840
Gemcitabine HCl	Selleck Chemicals	Cat# S1149
Trametinib (GSK1120212)	Selleck Chemicals	Cat# S2673
TR-107	Madera Therapeutics, LLC; Dr. Edwin Iwanowicz	N/A
Neurobasal A Media	Thermo Fisher Scientific	CAT#10888022
L-glutamine	Life Technologies Inc.	CAT#25030081
B-27™ Plus Supplement	Life Technologies Inc.	CAT#A3582801
N2 supplement	Life Technologies Inc.	CAT#17502048
Heparin	STEMCELL Technologies	CAT#07980
EGF	Shenandoah Biotechnology	CAT#100-26-100ug
FGF-basic 154	Shenandoah Biotechnology	CAT#100-146
Antibiotic-antimycotic	Thermo Fisher Scientific	CAT#15240062
Dulbecco's Modified Eagle Medium	Sigma	CAT#D6429-500ML
Fetal Bovine Serum	OMEGA SCIENTIFC	CAT#FB11FB-11
Penicillin-streptomycin	Sigma	CAT#P0781-100ML
DMEM/F12 with Glutamax	Thermo Fisher Scientific	CAT#10-565-018
Sodium Pyruvate	Life Technologies Inc.	CAT#11360070
MEM Non Essential Ammino Acids	Thermo Fisher Scientific	CAT#11-140-050

(*Continued on next page*)

Continued		
REAGENT or RESOURCE	SOURCE	IDENTIFIER
HEPES buffer	Thermo Fisher Scientific	CAT#15-630-080
GlutaMax	Thermo Fisher Scientific	CAT#35050061
B27 minus vitamin A	Thermo Fisher Scientific	CAT#12587010
PDGF-AA	Shenandoah Biotechnology	CAT#100-16
PDGF-BB	Shenandoah Biotechnology	CAT#100-18
Nuerobasal A minus phenol red	Thermo Scientific	CAT#12349015
Rat Serum	VWR	CAT#103219-652
Porcine Serum	VWR	CAT#103219-058
XenoLight D-Luciferin - K+ Salt Bioluminescent Substrate	PerkinElmer	CAT#122799
Propidium iodide, ≥94.0% (HPLC)	Sigma-Aldrich	CAT#P4170-10MG
Paraformaldehyde solution 4% in PBS	Santa Cruz Biotechnology	CAT#sc-281692
Triton X-100	Sigma-Aldrich	CAT#T9284-500ML
Deposited data		
Whole exome sequencing	This paper	dbGaP: phs003268.v1.p1
Experimental models: Cell lines		
LN-229	ATCC	RRID:CVCL_0393
GBM8	Gifted from H. Wakimoto	N/A
MDA-MB231-Br	Obtained through MTA with T. Yoneda	N/A
MS21	Derived from a GBM patient biopsy in Hingtgen Lab	N/A
U373WT	Provided by C. Vaziri	N/A
U373KO	Provided by C. Vaziri	N/A
IFF-BT105 PDIPG	Ian's Friends Foundation	N/A
Experimental models: Organisms/strains		
Rat: Sprague Dawley	Charles River Labs	RRID:MGI:5651135
Mouse: Female athymic nude	UNC Rodent Breeding Colony Management Core	N/A
Software and algorithms		
Fiji	ImageJ	https://imagej.net/software/fiji/
GraphPad Prism	GraphPad	https://www.graphpad.com
BioRender	N/A	https://biorender.com
Aura Imaging Software	Spectral Instruments	https://spectralinvivo.com/software/?utm_term=aura/20software&utm_campaign=/5BSearch/5D/5BBrand/5D&utm_source=adwords&utm_medium=ppc&hsa_acc=7526384108&hsa_cam=9975368639&hsa_grp=106473335891&hsa_ad=433511020441&hsa_src=g&hsa_tgt=kwd-355924183890&hsa_kw=aura/20software&hsa_mt=e&hsa_net=adwords&hsa_ver=3&gclid=Cj0KCQiAkMGcBhCSARIsAIW6d0Aqz8jVdzgj4D95hrrlI9MPJ-qF_tBTTc2iVJHbwIplJbJmf04GNxcaAgmrEALw_wcB
Living Image Software	Perkin Elmer	https://www.perkinelmer.com/product/spectrum-200-living-image-v4series-1-128113
LAS X	Lecia Microscopes	https://www.leica-microsystems.com/products/microscope-software/p/leica-las-x-ls/
ZEN	Zeiss	https://www.zeiss.com/microscopy/en/products/software/zeiss-zen.html

(*Continued on next page*)

Continued

REAGENT or RESOURCE	SOURCE	IDENTIFIER
Agilent SureSe-lectXT2 All Exon V6 kit	Agilent	https://www.agilent.com/cs/library/datasheets/public/SureSelect%20V6%20DataSheet%205991-5572EN.pdf
BBsplit from BBtools	Joint Genome Institute	https://jgi.doe.gov/data-and-tools/software-tools/bbtools/bb-tools-user-guide/bbmap-guide/
GATK/Picard v4.1.7.0 toolkit	Broad Institute	https://gatk.broadinstitute.org/hc/en-us/community/posts/360062628652-GATK-4-1-7-0-VariantAnnotator-Processing-Speed-Drop
MuTect2 algorithm v4.1.7.0	Broad Institute	https://gatk.broadinstitute.org/hc/en-us/articles/360037593851-Mutect2
SynergyFinder	Network Pharmacology for Precision Medicine in the Research Program of System Oncology, Faculty of Medicine at University of Helsinki	https://synergyfinder.org/
**Other**		
Millicell® Cell Culture Inserts	Millipore	PICM03050

RESOURCE AVAILABILITY

Lead contact

Further information and requests for resources and reagents should be directed to and will be fulfilled by the lead contact, Andrew Satterlee (satterle@e-mail.unc.edu).

Materials availability

This study did not generate new unique reagents.

Data and code availability

- Standardized WES dataset has been deposited on the dbGaP repository. dbGaP: phs003268.v1.p1.
- This paper does not report original code.
- Any additional information required to reanalyze the data reported in this paper is available from the lead contact upon request.

EXPERIMENTAL MODEL AND SUBJECT DETAILS

Animal ethics statement

All work performed on female athymic nude mice (therapy studies) or Sprague-Dawley rats (OBSC preparation) was approved by the Institutional Animal Care and Use Committee at the University of North Carolina-Chapel Hill. All OBSCs were generated from P8 Sprague-Dawley rat pups. Sprague-Dawley rats are housed with one mom and ten pups at a single time. Pups are used prior to needing to be weaned. All athymic nude mice are females and housed in grouped cages of 5 or less.

Human subjects ethics statement

Brain tumor samples were collected at the University of North Carolina Hospitals. Patients gave informed consent under protocol 20–1878 approved by the University of North Carolina's Institutional Review Board. All patient samples were de-identified through an honest broker prior to processing. A total of 10 patient cases (including recurrent cases) from both male and female subjects between the ages of 6–77 years old were included in the present study.All genders are listed in Table S4.

Cell lines

Cell lines used: LN229 (established GBM line), GBM8 (high-passage patient-derived GBM line[56]), MS21 (low-passage patient-derived GBM line), U373WT (wild-type established GBM line), U373KO (RAD18 knockout of U373WT via CRISPR), MB231Br (breast cancer metastasis to brain), and PDIPG (low-passage pediatric patient-derived line).

GBM8 cells were gifts from H. Wakimoto (Massachusetts General Hospital). GBM8 cells were cultured in Neurobasal-A medium (Gibco) with 7.5 mL L-glutamine, 10mL B27 supplement, 2.5 mL N2 supplement, 1 mg heparin, 10 μg EGF, 10 μg FGF, and 2.5 mL anti-anti. LN229 cells were from American Type Culture Collection. MDA-MB231-Br cells were obtained through a material transfer agreement (MTA) (T. Yoneda). MS21 cells were derived in the Hingtgen Laboratory from a GBM patient biopsy. U373WT and U373KO cells were provided by C. Vaziri (University of North Carolina). LN229, MDA-MB231-Br, MS21, U373WT and U373KO cells were cultured in Dulbecco's Modified Eagle Medium (Gibco) supplemented with 10% fetal bovine serum and 1% penicillin-streptomycin (Gibco). IFF-BT105 PDIPG cells were obtained from Ian's Friends Foundation and cultured in Neurobasal Medium(-A):DMEM/F-12, GlutaMAX Medium (1:1) supplemented with 1x Antibiotic-Antimycotic, 1x Sodium Pyruvate, 1x MEM Non-Essential Amino Acids, 10mM HEPES buffer, 1x GlutaMAX-I, 1X B27 minus vitamin A, 20 ng/ml EGF, 20 ng/ml bFGF, 10 ng/ml PDGF-AA, 10 ng/ml PDGF-BB, 2ug/ml heparin. All cell lines were cultured at 37°C, 5% CO_2 and 95% humidity.

Generating OBSCs

All OBSCs were generated from P8 Sprague-Dawley rat pups. Dissected brains were mounted on a vibratome (Leica VT1000S) platform submerged in ice-cold brain slice media (BSM). Coronal OBSCs were sliced at a thickness of 300 μm at ~15 OBSCs/animal. Visibly damaged brains or OBSCs were discarded. Acceptable OBSCs were transferred onto Millicell culture inserts in a 6-well culture plate. 1mL of OBSC media (BSM[22]) was added under each insert. BSM comprised of Neurobasal-A medium supplemented with 10% heat-inactivated pig serum, 5% heat-inactivated rat serum, 1 mM L-glutamine, 10 mM KCl, 10 mM HEPES, 1 mM sodium pyruvate and 100 U/mL penicillin-streptomycin. The plates were then transferred to a 37°C incubator with 5% CO2 and 95% humidity. For QC purposes, six random OBSCs from every batch were selected to undergo the PI assay test for cell death. On D4, the PI signals from the QC group were compared to those from previous batches.

In vivo tumor studies

6-8 week-old female athymic nude mice were used; n = 5 mice per cell line with no exclusion criteria, randomization, or blinding as all mice were used in one group. 250,000 tumor cells were stereotactically implanted into the brain parenchyma (1 mm, 1 mm, 2.5 mm) of mice anesthetized with isoflurane. All mice underwent serial bioluminescence imaging to measure tumor growth over time and were monitored for changes in weight or behavior to indicate the endpoint had been reached. Luciferin was injected i.p. into mice at 3mg/mouse in 250 mL PBS. Brains from each group, dissected at time of death, were coronally sectioned along the tumor implant site and imaged for tumor fluorescence.

METHOD DETAILS

Chemicals

The following chemicals were purchased from Sigma-Aldrich: carboplatin, temozolomide, etoposide, azacitidine, vincristine. The following chemicals were purchased from EMD Millipore: cisplatin. The following chemicals were purchased from Selleck Chemicals: lomustine, gemcitabine, and trametinib. TR-107 was generously provided by Dr. Edwin Iwanowicz (Madera Therapeutics, LLC).

Lentiviral vectors

The following LVs were used in this study: eGFP fused to firefly luciferase (LV–eGFP-FL) and mCherry protein fused to firefly luciferase (LV–mCh-FL).

Propidium iodide assay

PI fluorescence was used to quantify the health/quality of OBSCs as well as the toxicity of each treatment vs. OBSCs at the end of each assay: t = 4 days after OBSC generation and t = 3 days after treatment initiation. 5 μg/mL PI was mixed with BSM and added under culture inserts 60 min before fluorescence measurement on an AMI optical imaging system (Spectral Instruments). Positive control was generated by killing the OBSC via incubation with 70% ethanol and freezing overnight between days 3 and 4 of the assay. For all DSS calculations, n = 12 OBSCs from N = 2 separate experiments.

Immunohistochemistry

OBSCs dedicated for Day 0 IHC were fixed in 4% paraformaldehyde immediately following sectioning and stored at 4°C. After 48 h of fixation, the sections were transferred to 30% sucrose and stored at 4°C until IHC was conducted. The sections dedicated for Day 4 IHC were transferred to 6 well plates with BSM and stored at 37°C. GBM8-mch-FLuc cells were engrafted atop select OBSCs as described below. On Day 1, the sections were transferred to new 6 well plates with fresh BSM. The sections were fixed in 4% paraformaldehyde on Day 4 and stored at 4°C. After 48 h of fixation, the sections were transferred to 30% sucrose and stored at 4°C. When day 0 and day 4 sections were ready for IHC, they were first washed for 10 min in 0.1% Triton X-100 in 1X Dulbecco's phosphate-buffered saline (PBST) at room temperature. Sections were then blocked in 5% fetal bovine serum in PBST for 1h at room temperature. The sections were incubated in a primary antibody solution consisting of primary antibodies and blocking buffer rotating overnight at room temperature. The primary antibodies used were glial fibrillary acidic protein (GFAP [Abcam, ab7260] at 1:1000), neuronal nuclear protein (NeuN [Abcam, ab177487] at 1:1000), and cluster of differentiation molecule 11B (CD11B [Abcam,

ab133357], 1:500). The sections were washed 3 times in PBST for 10 min after 18–24 h in primary antibodies. They were then incubated in a secondary antibody consisting of blocking buffer solution and Alexa Fluor 488 goat anti-rabbit IgG (Thermo Fisher Scientific, A-11008, 1:1000) for 1 h in a darkroom. The sections were washed 3 times in PBST for 10 min and mounted on microscopic slides. Liquid mountant (Pro-Long Gold Antifade Mountant) was applied on the sections, coverslips were added, and slides were allowed to cure overnight. z stack images were acquired using a Zeiss 780 confocal microscope at UNC Neuroscience Microscopy Core. z stack images are processed by converting to maximum intensity projection (Max IP) images. The brightness of Max IP images was then further adjusted to accurately assess and present the morphological differences of astrocytes.

In vitro studies

Cells were seeded at a density of 5000 cells/μL in 96 well plates. 24h after seeding, drugs were added and allowed to incubate for 72 h. Cell viability was then assessed via bioluminescence, measured using an AMI optical imaging system.

Tumor growth on OBSCs

Tumor cells (0.17 μL, 25,000 cells) were seeded onto OBSCs 2h after slicing, with one tumor seeded in the center of each hemisphere for a total of two tumor foci per OBSC. BSM was changed 24h after slicing. Fluorescence images were taken 0 h, 24 h, and 96 h post-seeding for normalization of tumor size. Bioluminescence readouts were also taken at 0 h, 24 h, and 96 h post seeding for assessment of cell viability. Luciferin was added underneath the culture insert and allowed to incubate for 10 min before bioluminescence measurement on an AMI optical imaging system.

Drug screening on OBSCs

Tumor cells were seeded onto OBSCs 2h after slicing as described above. One day after tumor seeding, six concentrations of each drug were diluted in the media underneath each culture insert, or XRad was applied using an X-Rad 320 Precision X-ray machine ($n = 4$ tumor foci per concentration; $n \geq 24$ tumor foci per drug per cell line). Three days after treatment, the bioluminescence of live tumor cells was measured using an AMI optical imager (Spectral Instruments) and dose-response curves were generated. Tumor survival was normalized to day one tumor fluorescence and compared to an untreated control group.

Combination therapy

Carboplatin + etoposide: On Day 1 after slicing, both small molecules were administered in the BSM underneath the culture inserts at the desired concentrations.

TMZ + radiation: On Day 1 after slicing, radiation was first administered by an X-Rad 320 Precision X-ray machine. TMZ was then administered in the BSM underneath the inserts at the desired concentrations.

Preparing patient tissue for engraftment onto OBSCs

Fresh brain tumor tissue surgically resected at UNC hospitals was placed in sterile 4°C Neurobasal-A medium and immediately taken to the UNC Tissue Procurement Facility (TPF). The amount of brain tumor tissue received ranged from 0.05g to 2g. Either at TPF or in the Hingtgen Laboratory, the resected tumor tissue was minced into approximately 0.5 mm diameter pieces using a disposable scalpel and washed with PBS. Tumor pieces which were to be assayed at a later time were placed in a cryogenic vial and frozen in tissue freezing medium (CryoStor CS10) in a FreezeCell at - 80°C overnight before transfer into liquid nitrogen.

To engraft onto OBSCs, the cryopreserved/thawed or fresh brain tumor tissue was filtered through a 100 μm cell strainer. Each 50 mg of tissue was transfected with 1mL mcherry-FLuc Lentivirus at 1.5e7 vg/ml with 1μL polybrene for 4h at 37°C. After incubation, the brain tumor tissue was washed with PBS three times to remove the residual virus and reconstituted in PBS with a final volume of 150 μL. Then the tissue solution was engrafted atop OBSCs at ~0.5 mg tissue in 3 μL of PBS on each hemisphere of the slice. The OBSC engrafted with patient tumor tissue was incubated at 37°C with 5% CO_2 and 95% humidity. The BSM was changed after 24h and subsequently every 3 days. Tumor treatment studies were executed as described above for tumor lines. The viability of the patient tissue was measured by an AMI optical imaging system with the addition of 1.5 mg/mL luciferin underneath the culture insert.

Patient tumor drug screening results were not shown to clinicians with information that could identify the patients they reference.

Preparing cell line from patient tissue

For the primary patient cell line generation, the fresh or frozen tissue were mechanically dissected to approximately single cell under sterile conditions. The resulting single-cell suspension was then washed with PBS, resuspended in growth medium chosen to allow growth of greatest number of tumor cells (DMEM with 20% FBS and 1% PS), and dispensed into 6-well plates. All cultures were initiated in a volume of 2 mL per well and incubated at 37°C and 5% CO_2. The viable cells and tissue chunks were allowed to settle and attach to the bottom of the plates for 2–3 days. The floating cellular debris was then carefully aspirated, the attached cells were carefully washed with PBS, and 2 mL of fresh medium was added to each well. Culture medium was routinely changed every 3–5 days, and proliferating adherent cells were passaged after detachment with trypsin.

DNASeq

Cryopreserved/thawed human brain tumor tissue was prepared as described above and either added onto OBSCs (BSHT group), cultured *in vitro* (CL group) or snap frozen (HT group). After 4 days, tumor tissue was carefully excised from OBSCs using forceps and snap frozen. After 30 days, when cell line had established, cells were collected and snap frozen. All replicates of all samples were shipped to Novogene, Inc. for DNA sequencing. Whole Exome Sequencing was run on the DNA samples following library preparation with an Agilent SureSe-lectXT2 All Exon V6 kit. Raw data was downloaded and analyzed at the UNC Bioinformatics core facility.

The BBsplit algorithm from the BBtools suite was run on all samples to eliminate rat DNA contamination in the BSHT samples as well as to account for any biases that may result as a part of that process. Only the reads that were binned to the human reference were used for subsequent analysis. Reads were then mapped to the GRCh38 version of the human genome with BWA v0.7.17 and realigned together with ABRA2 v.23. Quality control was implemented using the GATK/Picard v4.1.7.0 toolkit. Somatic variants were called for each sample using the MuTect2 algorithm v4.1.7.0. Variants were merged into a single cohort variant call file and then converted to MAF via vcf2maf v1.6.21 tool. Variants were annotated using VEP v87. To identify mutations with potentially high biological impact, multiple filtering steps were applied to somatic mutation calling. First, we selected only the somatic variants that passed all filters from the MuTect2 FilterMutectCalls algorithm and second, only high/moderate impact (change coding) variants as defined by the VEP annotation were further analyzed. Over 1900 single nucleotide variants (SNVs) were detected across all samples. Figures that summarized the results were generated using maftools.

QUANTIFICATION AND STATISTICAL ANALYSIS

Calculating drug sensitivity scores

Several weighted parameters were factored into an overall drug sensitivity score. DSS were calculated by comparing the tumor cell survival, measured via BLI, to the health of the OBSC, measured via PI assay (parameter 1–8), or by quantifying the behavior of the tumor dose-response curve (parameter 9–11).

(1) Killing at maximum dose (Max Kill Window, 10% of DSS)
(2) Dose required to kill 10% of the tumor (EC10 Window, 5% of DSS)
(3) Dose required to kill 25% of the tumor (EC25 Window, 5% of DSS)
(4) Dose required to kill 50% of the tumor (EC50 Window, 10% of DSS)
(5) Dose required to kill 75% of the tumor (EC75 Window, 5% of DSS)
(6) Dose required to kill 90% of the tumor (EC90 Window, 5% of DSS)
(7) Slope through the EC50 (Slope Window, 10% of DSS)
(8) The area under the dose-response curve (AUC Window, 35% of DSS)
(9) Tumor growth acceleration (treated tumor grows faster than untreated tumor: for max growth up to 125%, window = +1; 125%–150% window = 0; over 150% window = −1; 5% of DSS)
(10) Biphasic killing (rapid killing at low doses and limited additional killing at higher doses: no biphasic curve shape, window = +1; biphasic curve shape, window = −1; 5% of DSS)
(11) Incomplete kill at the highest dose (some tumor remaining at highest dose: for <10% remaining, window = +1; 10%–25% remaining, window = 0; >25% remaining, window = −1; 5% of DSS).

For DSS parameters 1–6, therapeutic windows were calculated by comparing OBSC toxicity and tumor response at the doses where tumor kill passed through the DSS parameter (Table S1 and S3). For DSS parameter 7, the therapeutic window was calculated by comparing the slopes through the tumor EC50 and the OBSC tox EC50. For DSS parameter 8, the therapeutic window was calculated by comparing the areas under tumor kill and OBSC tox curves. Normalized therapeutic window ratios for DSS parameters 1–8 within each drug-tumor-OBSC interaction were calculated in the following manner: within each window, values ranged from +1 to −1, where values approaching +1 signify increasing tumor kill relative to normal tissue toxicity, and values approaching −1 indicate agents where tumors remained highly viable while toxicity to the normal OBSC tissue was elevated. DSS parameters 9–11 were determined based on the behavior of the tumor in response to the drug, as defined above.

All individually weighed parameters were added together to generate the DSS. Overall DSS from 0 to 100 signify increasing efficacy in tumor kill relative to OBSC toxicity, while scores from 0 to −100 describe scenarios in which tumors thrive more effectively than OBSCs for a given treatment. Dose-response values were calculated via linear interpolation of raw data, not from best-fit curve equations.

Calculating ZIP synergy scores and plots

All synergy scores and plots were calculated using https://synergyfinder.org/

Statistical analysis

All statistical tests and sample sizes are included in the Figure Legends. All data are shown as mean ± SEM. In all cases, the p values are represented as follows:***$p < 0.001$, ** $p < 0.01$, *$p < 0.05$, and not statistically significant when $p > 0.05$. In all cases, the stated ‘‘n’’

value is either number of OBSCs, number of tumor spots placed on the OBSCs, or mice with multiple independent images used to obtain data points for each. Mean values between two groups were compared using t-tests with Welch's correction when variances were deemed significant by F tests. Mean values between three or more groups were compared to the control by using one-way ANOVA followed by Dunnett's multiple comparisons test. All statistical analyses were performed using GraphPad Prism (Version 9.1.0). All statistical analysis methods and resulting p values are included within the Figure Legends. For all quantifications of immunohistology, the samples being compared were processed in parallel and imaged using the same settings and laser power.

Cell Reports Medicine, Volume *4*

Supplemental information

A living ex vivo platform for functional, personalized brain cancer diagnosis

Breanna Mann, Xiaopei Zhang, Noah Bell, Adebimpe Adefolaju, Morrent Thang, Rajaneekar Dasari, Krishna Kanchi, Alain Valdivia, Yang Yang, Andrew Buckley, Vivien Lettry, Carolyn Quinsey, Yasmeen Rauf, David Kram, Noah Cassidy, Cyrus Vaziri, David L. Corcoran, Stephen Rego, Yuchao Jiang, Lee M. Graves, Denise Dunn, Scott Floyd, Albert Baldwin, Shawn Hingtgen, and Andrew B. Satterlee

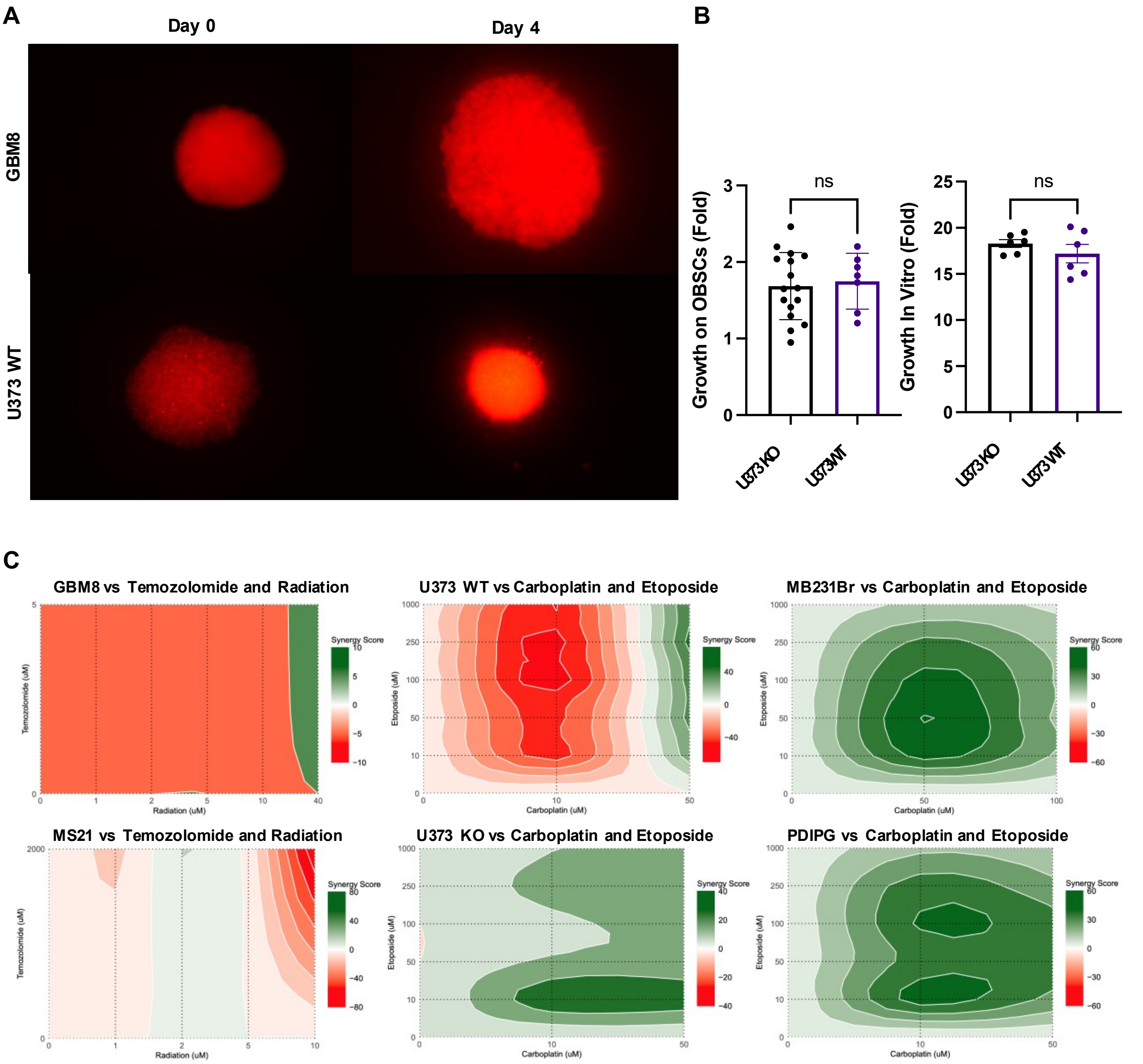

Figure S1, Related to Figures 2 and 3: Additional Tumor Growth and Synergy Plots. A) Fluorescence images of GBM8 and U373 WT tumor foci on day 0 and day 4. B) Comparison of U373WT and U373KO growth on OBSCs and *in vitro*. (n□6 technical replicates per group). Analyzed using an unpaired t-test. C) ZIP Synergy Score plots representing synergistic or antagonistic effects of combination therapies displayed in Fig 3C.

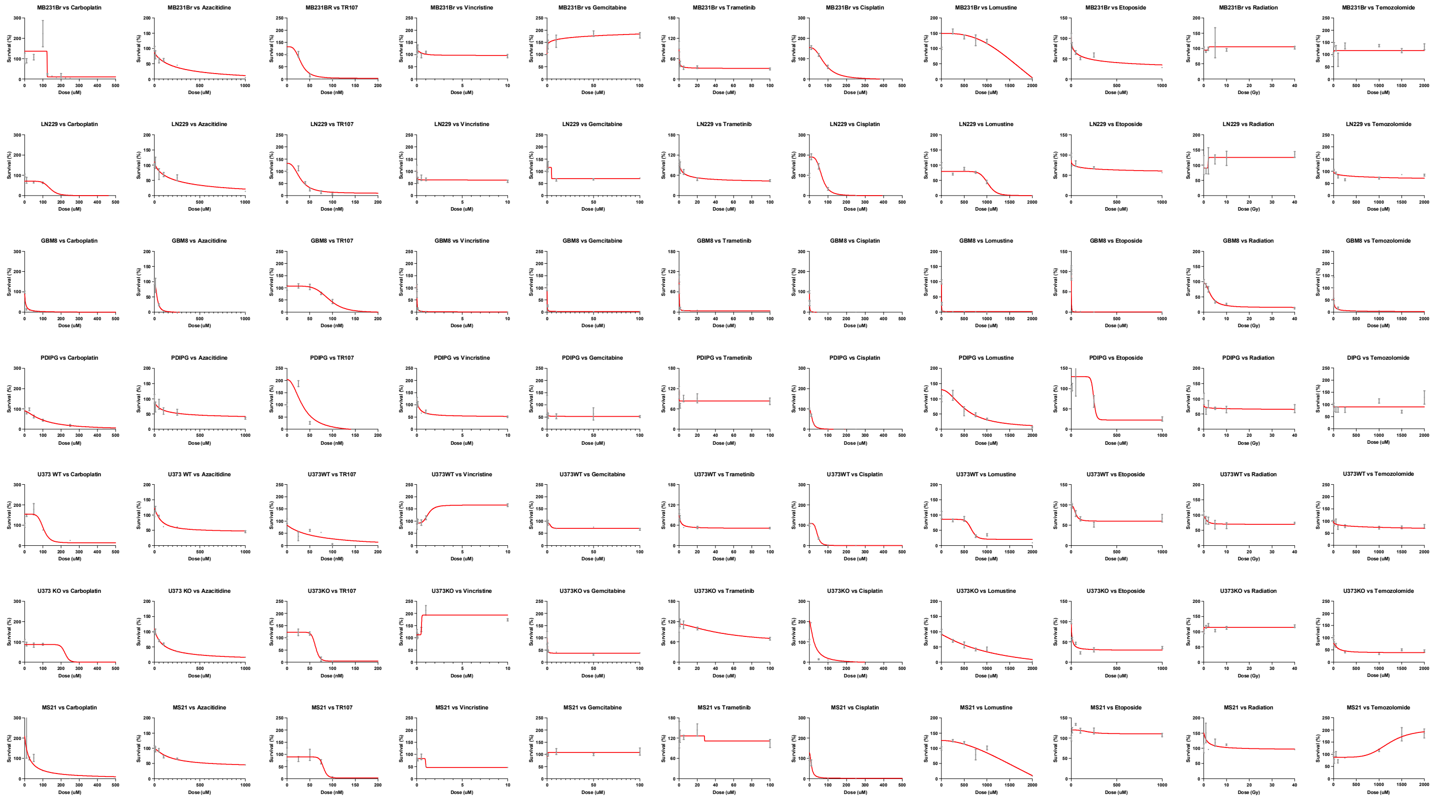

Figure S2, Related to Figure 3 and 4: Killing Curves on OBSCs. Dose-response curves of each cell line against each therapeutic. To enable qualitative comparison, all datasets are fit with an inhibitor vs response variable slope (four parameters) least squares fit. This method poorly fits some curve types, such as those with increases in growth at low concentrations. Our IC50 and DSS calculations do not use these fitted curves, but instead use linear interpolation between calculated data points.

	Carboplatin	Azacitidine	Temozolomide	TR107	Vincristine	Gemcitabine	Trametinib	Cisplatin	Lomustine	Etoposide	Radiation
**MB231BR**											
Killing at highest dose	1.00E+00	9.50E-01	-3.30E-01	9.99E-01	7.00E-02	-7.90E-01	7.00E-01	1.00E+00	9.70E-01	7.20E-01	-2.00E-02
EC10	1.17E+02	4.76E+00	NR	2.84E-02	NR	NR	3.23E-02	7.41E+01	1.28E+03	5.26E+00	NR
EC25	1.19E+02	1.89E+01	NR	3.27E-02	NR	NR	8.06E-02	8.71E+01	1.40E+03	2.50E+01	NR
EC50	1.22E+02	2.08E+02	NR	3.98E-02	NR	NR	7.84E-01	1.25E+02	1.61E+03	1.00E+02	NR
EC75	1.25E+02	6.25E+02	NR	4.69E-02	NR	NR	NR	1.88E+02	1.82E+03	NR	NR
EC90	2.13E+02	9.06E+02	NR	5.72E-02	NR	NR	NR	2.25E+02	1.94E+03	NR	NR
Slope	-8.00E-02	-1.20E-03	NR	NR	-1.89E-02	1.56E-02	-2.78E-01	-4.00E-03	-1.20E-03	-3.00E-03	NR
AUC	1.89E+02	3.36E+02	2.58E+03	4.17E-02	1.02E+01	1.74E+02	3.32E+01	6.20E+01	1.96E+03	4.75E+02	4.02E+01
**LN229**											
	Carboplatin	Azacitidine	Temozolomide	TR107	Vincristine	Gemcitabine	Trametinib	Cisplatin	Lomustine	Etoposide	Radiation
Killing at highest dose	1.00E+00	8.60E-01	3.50E-01	1.00E+00	4.10E-01	2.80E-01	5.60E-01	1.00E+00	1.00E+00	4.40E-01	-3.60E-01
EC10	4.17E+00	2.89E+01	5.94E+01	3.06E-02	6.67E-03	6.23E+00	6.68E-01	7.46E+01	4.17E+02	3.70E+00	NR
EC25	1.40E+01	4.56E+01	4.60E+02	3.44E-02	4.18E-01	8.40E+00	3.46E+00	8.13E+01	7.58E+02	9.26E+00	NR
EC50	1.33E+02	4.00E+02	NR	4.11E-02	NR	NR	1.86E+01	9.24E+01	9.60E+02	NR	NR
EC75	1.93E+02	8.17E+02	NR	4.98E-02	NR	NR	NR	1.36E+02	1.44E+03	NR	NR
EC90	2.29E+02	NR	NR	9.00E-02	NR	NR	NR	2.05E+02	1.78E+03	NR	NR
Slope	-4.20E-03	-6.00E-04	-4.00E-05	-2.87E+01	-8.89E-03	-5.45E-03	-1.47E-02	-2.20E-03	-1.24E-03	-1.73E-04	NR
AUC	1.22E+02	4.51E+02	1.53E+03	6.08E-02	6.37E+00	7.06E+01	4.96E+01	7.69E+01	9.70E+02	6.48E+02	5.01E+01
**GBM8**											
	Carboplatin	Azacitidine	Temozolomide	TR107	Vincristine	Gemcitabine	Trametinib	Cisplatin	Lomustine	Etoposide	Radiation
Killing at highest dose	1.00E+00	1.00E+00	1.00E+00	1.00E+00	1.00E+00	9.80E-01	9.80E-01	1.00E+00	1.00E+00	1.00E+00	8.69E-01
EC10	1.16E+00	8.33E+00	1.52E+00	6.30E-02	4.35E-03	6.76E-02	3.13E-02	9.06E-02	6.85E-01	5.58E-01	8.33E-01
EC25	2.91E+00	1.81E+01	3.79E+00	7.65E-02	1.29E-02	1.69E-01	7.81E-02	2.26E-01	1.71E+00	1.74E+00	2.04E+00
EC50	5.81E+00	3.38E+01	7.58E+00	9.53E-02	4.86E-02	3.38E-01	3.84E-01	4.53E-01	3.43E+00	4.60E+00	3.84E+00
EC75	8.72E+00	4.94E+01	3.12E+01	1.21E-01	8.43E-02	5.33E-01	7.79E-01	2.74E+00	5.45E+00	7.47E+00	1.37E+01
EC90	2.60E+01	8.33E+01	1.67E+02	1.39E-01	2.60E-01	2.00E+00	1.67E+00	4.20E+00	8.71E+00	9.19E+00	NR
Slope	-8.60E-02	-1.60E-02	-6.60E-02	-1.33E+01	-7.00E+00	-1.48E+00	-6.33E-01	-1.10E+00	-1.46E-01	-8.73E-02	-1.38E-01
AUC	1.03E+01	4.08E+01	9.52E+01	9.94E-02	2.38E-01	2.79E+00	4.78E+00	1.76E-01	1.54E+01	8.40E+00	1.09E+01
**PDIPG**											
	Carboplatin	Azacitidine	Temozolomide	TR107	Vincristine	Gemcitabine	Trametinib	Cisplatin	Lomustine	Etoposide	Radiation
Killing at highest dose	1.00E+00	6.40E-01	2.90E-01	1.00E+00	4.86E-01	4.80E-01	1.70E-01	1.00E+00	9.30E-01	7.50E-01	3.20E-01
EC10	4.76E+00	3.70E+00	5.00E+00	4.02E-02	2.42E-01	1.14E-01	3.00E+01	3.17E+00	3.54E+02	2.22E+02	1.11E+00
EC25	2.00E+01	9.26E+00	6.67E+02	4.25E-02	4.06E-01	2.84E-01	NR	7.94E+00	4.25E+02	2.42E+02	3.62E+00
EC50	8.61E+01	4.47E+02	1.06E+03	4.64E-02	NR	NR	NR	1.68E+01	6.88E+02	5.74E+02	NR
EC75	2.11E+02	NR	NR	5.19E-02	NR	NR	NR	2.73E+01	1.31E+03	1.00E+03	NR
EC90	5.83E+02	NR	NR	6.57E-02	NR	NR	NR	4.15E+01	1.88E+03	NR	NR
Slope	-3.59E-03	-2.53E-04	-4.80E-05	-6.40E+01	-2.32E-02	-3.03E-04	-1.00E-03	-2.73E-02	-4.80E-04	-5.87E-04	-2.86E-04
AUC	1.79E+02	5.03E+02	1.88E+03	6.60E-02	6.36E+00	5.72E+01	8.73E+01	4.87E+00	9.11E+02	6.75E+02	2.71E+01
**U373 WT**											
	Carboplatin	Azacitidine	Temozolomide	TR107	Vincristine	Gemcitabine	Trametinib	Cisplatin	Lomustine	Etoposide	Radiation
Killing at highest dose	1.00E+00	5.40E-01	2.20E-01	9.80E-01	-6.60E-01	3.30E-01	4.80E-01	1.00E+00	9.10E-01	3.30E-01	2.60E-01
EC10	9.32E+01	6.11E+01	6.73E+01	1.32E-02	8.20E-01	3.85E-01	6.25E-01	2.03E+01	1.39E+02	2.56E+01	5.88E-01
EC25	1.03E+02	8.19E+01	9.62E+01	3.29E-02	NR	5.00E+01	2.16E+00	2.84E+01	5.54E+02	6.54E+01	3.50E+00
EC50	1.76E+02	8.00E+02	NR	7.65E-02	NR	NR	NR	4.19E+01	6.65E+02	2.60E+02	NR
EC75	2.50E+02	NR	NR	8.93E-02	NR	NR	NR	6.52E+01	1.38E+03	NR	NR
EC90	7.00E+02	NR	NR	9.69E-02	NR	NR	NR	8.79E+01	1.96E+03	NR	NR
Slope	-3.40E-03	-2.00E-04	-9.14E-06	-1.96E+01	5.78E-02	-1.60E-03	-2.50E-04	-1.85E-02	-2.24E-03	-1.26E-04	-6.50E-03
AUC	3.11E+02	5.88E+02	1.52E+03	6.00E-02	1.36E+01	7.29E+01	5.40E+01	1.33E+01	8.89E+02	6.12E+02	2.82E+01
**U373 KO**											
	Carboplatin	Azacitidine	Temozolomide	TR107	Vincristine	Gemcitabine	Trametinib	Cisplatin	Lomustine	Etoposide	Radiation
Killing at highest dose	9.99E-01	8.30E-01	5.40E-01	9.50E-01	-7.40E-01	6.20E-01	3.10E-01	1.00E+00	9.70E-01	6.38E-01	-1.70E-01
EC10	9.09E+00	2.38E+01	3.85E+00	5.67E-02	NR	1.39E-01	4.58E+01	3.32E+01	8.33E+01	2.42E+00	NR
EC25	1.25E+02	4.45E+01	9.62E+00	6.02E-02	NR	3.47E-01	8.45E+01	3.65E+01	2.08E+02	6.06E+00	NR
EC50	1.72E+02	1.58E+02	1.16E+03	6.62E-02	NR	8.68E-01	NR	4.20E+01	6.07E+02	3.78E+01	NR
EC75	2.19E+02	6.47E+02	NR	7.21E-02	NR	NR	NR	4.76E+01	1.46E+03	NR	NR
EC90	2.48E+02	NR	NR	8.33E-02	NR	NR	NR	1.14E+02	1.83E+03	NR	NR
Slope	-5.29E-03	-1.73E-03	-8.67E-04	-4.20E+01	NR	-3.80E-01	-3.88E-03	-4.53E-02	-5.60E-04	-3.13E-03	4.25E-03
AUC	1.92E+02	3.39E+02	8.89E+02	9.00E-02	1.89E+01	3.67E+01	8.91E+01	1.56E+01	8.35E+02	3.37E+02	4.54E+01
**MS21**											
	Carboplatin	Azacitidine	Temozolomide	TR107	Vincristine	Gemcitabine	Trametinib	Cisplatin	Lomustine	Etoposide	Radiation
Killing at highest dose	1.00E+00	5.50E-01	-8.20E-01	9.68E-01	5.30E-01	-1.20E-01	-5.00E-02	9.99E-01	9.10E-01	-8.00E-02	5.00E-02
EC10	5.97E+01	3.15E+01	-5.00E+02	5.74E-02	5.26E-03	NR	NR	4.55E+00	1.12E+03	NR	NR
EC25	7.18E+01	9.09E+01	4.17E+02	7.04E-02	1.72E-01	NR	NR	1.16E+01	1.28E+03	NR	NR
EC50	9.19E+01	8.21E+02	8.33E+02	8.26E-02	4.60E+00	NR	NR	2.47E+01	1.55E+03	NR	NR
EC75	1.57E+02	NR	1.25E+03	9.23E-02	NR	NR	NR	3.79E+01	1.83E+03	NR	NR
EC90	2.15E+02	NR	1.50E+03	9.82E-02	NR	NR	NR	4.57E+01	1.99E+03	NR	NR
Slope	-1.24E-02	-2.80E-04	5.47E-04	-2.58E+01	-5.56E-03	NR	NR	-1.90E-02	-9.20E-04	NR	NR
AUC	1.47E+02	6.06E+02	2.37E+03	8.81E-02	5.22E+00	1.06E+02	1.26E+02	4.20E+00	1.61E+03	1.15E+03	4.25E+01

Table S1, Related to Figure 3 and 4: Killing Parameters on OBSCs. Dose-response values used to generate DSS from each cell line against each therapeutic. Values for Killing at Highest Dose are given as fraction of tumor population killed. EC values are given as small molecule drug concentrations in μM or as XRad dose in Gy. NR indicates the IC50 was not reached within the dose range. Linear interpolation between calculated data points used to calculate individual EC values. Trapezoidal rule used to calculate AUC.

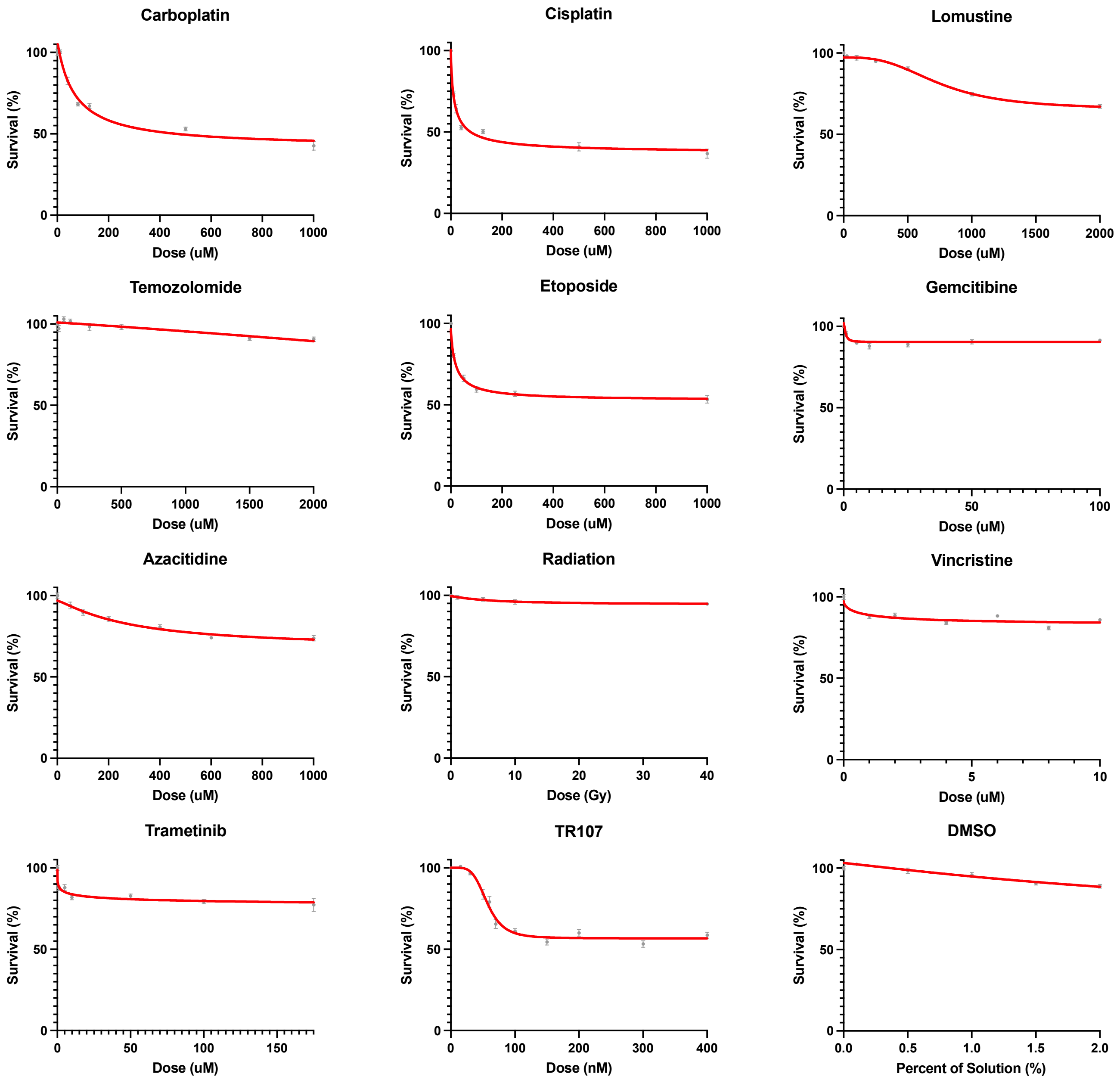

Figure S3, Related to Figure 4: OBSC Treatment Toxicity. Dose-response curves of OBSC survival via PI stain against each therapeutic. To enable qualitative comparison, all datasets are fit with an inhibitor vs response variable slope (four parameters) least squares fit. This method poorly fits some curve types, such as those with increases in growth at low concentrations. Our IC50 and DSS calculations do not use these fitted curves, but instead use linear interpolation between calculated data points.

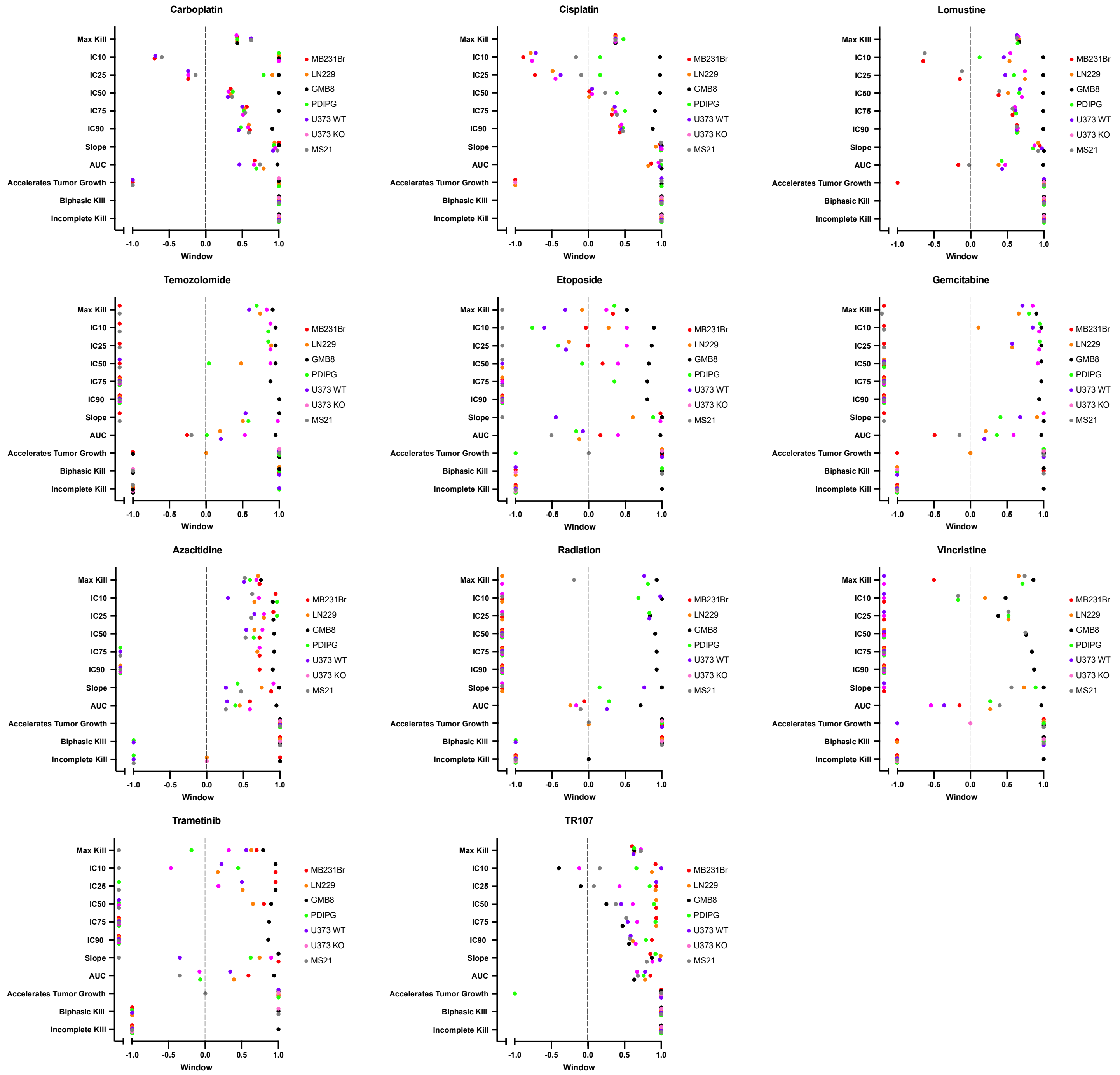

Figure S4, Related to Figure 4: DSS Window Plots. Therapeutic windows across all 11 DSS parameters for all drugs vs all tumor lines. Windows closer to 1 indicate more selective killing of the tumor. Windows closer to -1 indicate more selective killing of the OBSC. Windows left of -1 indicate the parameter was never reached. Windows for each parameter were weighted and summed to calculate a single DSS for each drug-tumor-OBSC interaction. Windows were calculated via linear interpolation of raw data, not from best-fit curve equations.

Top 250 Most Significantly Mutated Somatic Genes - From MMG-II DNAseq									
1	CCL4L2	51	USP2	101	ADGRB3	151	NDNF	201	TRIM41
2	FLG2	52	WASHC5	102	AGK	152	NPIPB5	202	TRPM2
3	NF2	53	XKR9	103	ANKRD18A	153	NRXN1	203	UBE2J1
4	ANKRD36	54	ZNF518A	104	ANKRD33B	154	NRXN2	204	UGT2A1
5	IGHV3-30	55	CENPB	105	AP4B1	155	NTN1	205	USP6
6	IGHV3-38	56	GNAS	106	APBB2	156	NUP160	206	WDFY4
7	TRBV6-8	57	IGHV3-11	107	BBS7	157	NUP58	207	WDR24
8	KCNE1B	58	IGHV3-53	108	BCL11A	158	OR4C45	208	WDR87
9	MUC4	59	MCU	109	BOP1	159	OR4Q3	209	WWC2
10	OR4M2B	60	NIBAN3	110	BRD8	160	PCDHB10	210	XPC
11	AGAP1	61	NID1	111	C10orf88	161	PDZRN4	211	ZNF138
12	AP000721.1	62	PRAMEF9	112	CDC45	162	PIGG	212	ZNF208
13	ARHGAP21	63	ACSL4	113	CELSR3	163	PLEKHG6	213	ZNF408
14	ARID4B	64	BACE1	114	CIT	164	POLD1	214	ZNF540
15	BHLHE41	65	CCZ1B	115	COL12A1	165	POLE	215	AGAP9
16	COLQ	66	IRS2	116	DMXL1	166	PPP1R12A	216	AMBRA1
17	CRLF1	67	KMT2B	117	DOCK6	167	PRAMEF14	217	ANKRD36B
18	DHCR7	68	MUC5AC	118	DOCK7	168	PRDM11	218	ATXN1L
19	DHX8	69	AGFG1	119	DRG2	169	PRPF4	219	BHLHA9
20	EIF3M	70	BNC2	120	EIF4G2	170	RAB1A	220	CASR
21	FAM160B2	71	C5orf34	121	EVC2	171	RELCH	221	CAT
22	FAM47A	72	C6orf62	122	FLG	172	RNF212B	222	CCDC15
23	FN1	73	CACNA1D	123	FO681492.1	173	RPAP3	223	CDIN1
24	GABRR1	74	CDS1	124	FUT11	174	RPGRIP1L	224	CKB
25	GTF3C2	75	DNAH1	125	GOLGA6L1	175	RSF1	225	CRAMP1
26	HIBCH	76	EPS8L2	126	GOLGA6L9	176	RSPH1	226	CSMD3
27	HIRIP3	77	FOXO6	127	GPN1	177	RWDD3	227	CYLC1
28	HIVEP1	78	GLI3	128	GTPBP8	178	SCN10A	228	DSCAML1
29	HSF4	79	IFFO1	129	HNRNPLL	179	SERAC1	229	ENOX2
30	KIAA0319	80	KANSL2	130	HOXD8	180	SERINC4	230	EPC2
31	LILRA6	81	LZTS1	131	HRC	181	SH3TC2	231	EPG5
32	MAP3K6	82	MSI2	132	HTR2B	182	SLC12A6	232	ERC1
33	OR51B6	83	NBPF14	133	IGHV2-70D	183	SLC13A4	233	FAM120B
34	OR5L2	84	NCOA6	134	IGHV4-4	184	SLC18A1	234	FBXO38
35	P3H2	85	NOVA1	135	ITIH6	185	SLC7A4	235	FHOD1
36	PLEKHS1	86	NPIPA5	136	IVD	186	SOX21	236	FOXN3
37	PLPPR3	87	NSD1	137	KANSL1	187	SOX3	237	HAT1
38	PRR29	88	PCDH19	138	KCNIP4	188	SPATA7	238	HERPUD1
39	PSG3	89	PTCHD4	139	KIAA1549	189	SULT1A1	239	HIPK1
40	RRAGC	90	PUM1	140	KIR3DL3	190	SYBU	240	HOXC11
41	RTN3	91	RALGAPA1	141	LAMC1	191	TBC1D3B	241	HPS1
42	RTP3	92	RNF11	142	LILRB3	192	TBXT	242	IL1RAPL2
43	SCAF11	93	RSPO4	143	LTN1	193	TEX10	243	KAT6B
44	SPTBN2	94	SLF1	144	LYSMD4	194	THADA	244	KIR2DL4
45	ST6GALNAC3	95	TRBV6-5	145	MBD5	195	TMEM164	245	KMT2A
46	SYNJ2	96	WNK2	146	MIDEAS	196	TMEM267	246	KMT2D
47	TMEM272	97	ZNF326	147	MTF2	197	TMPRSS15	247	KRTAP10-7
48	TRMT10B	98	AARS2	148	MYT1L	198	TNK2	248	MSL1
49	TYRO3	99	ABCA13	149	NBPF20	199	TNKS1BP1	249	PHF12
50	UNC93A	100	ADAM30	150	NBPF26	200	TOX	250	TCERG1

Table S2, Related to Figure 5: Top 250 Most Significantly Mutated Somatic Genes – From MG-II DNAseq.

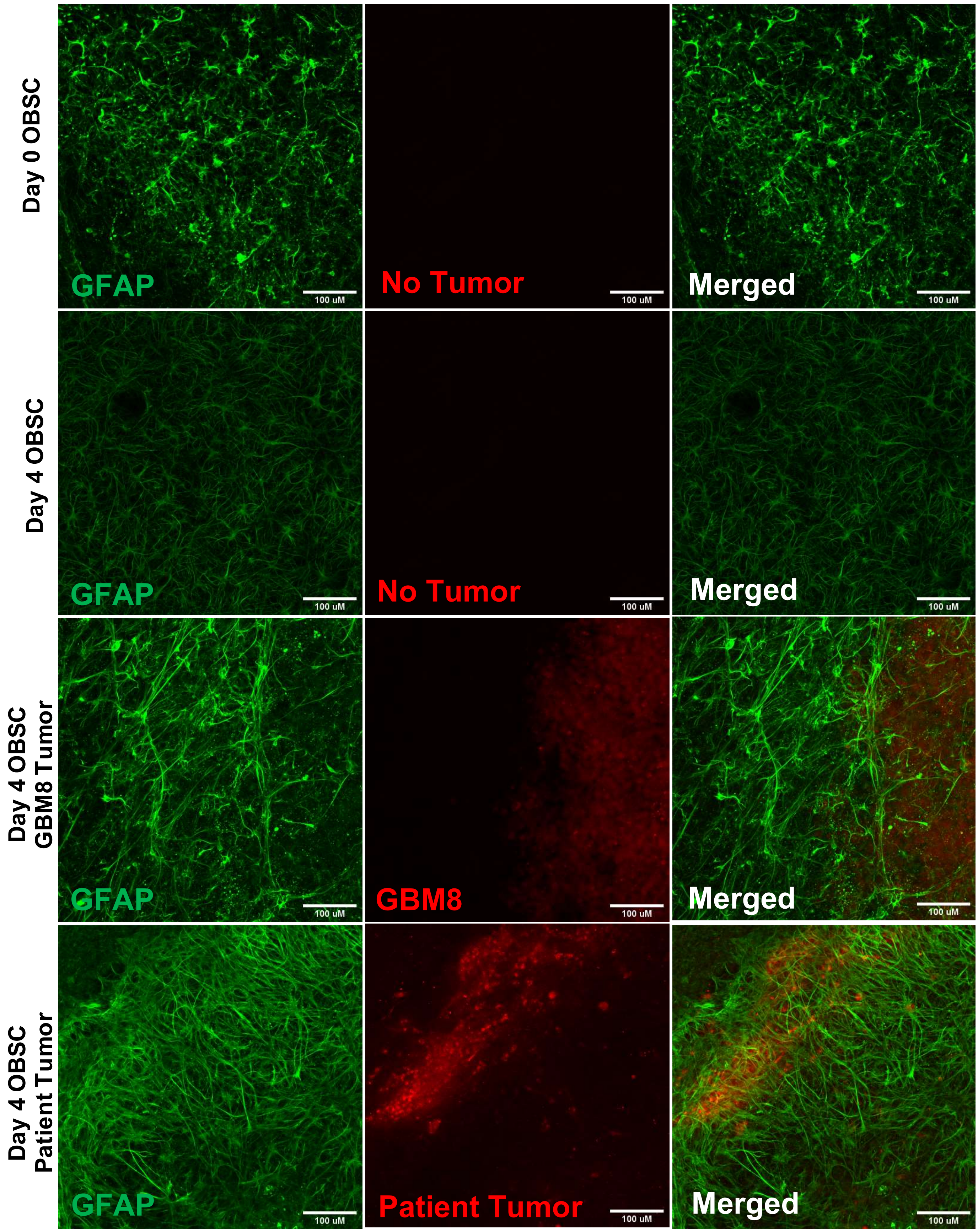

Figure S5, Related to Figures 1, 2, and 5: High-res IHC Images: High-resolution versions of IHC images shown in Figures 1, 2, and 5.

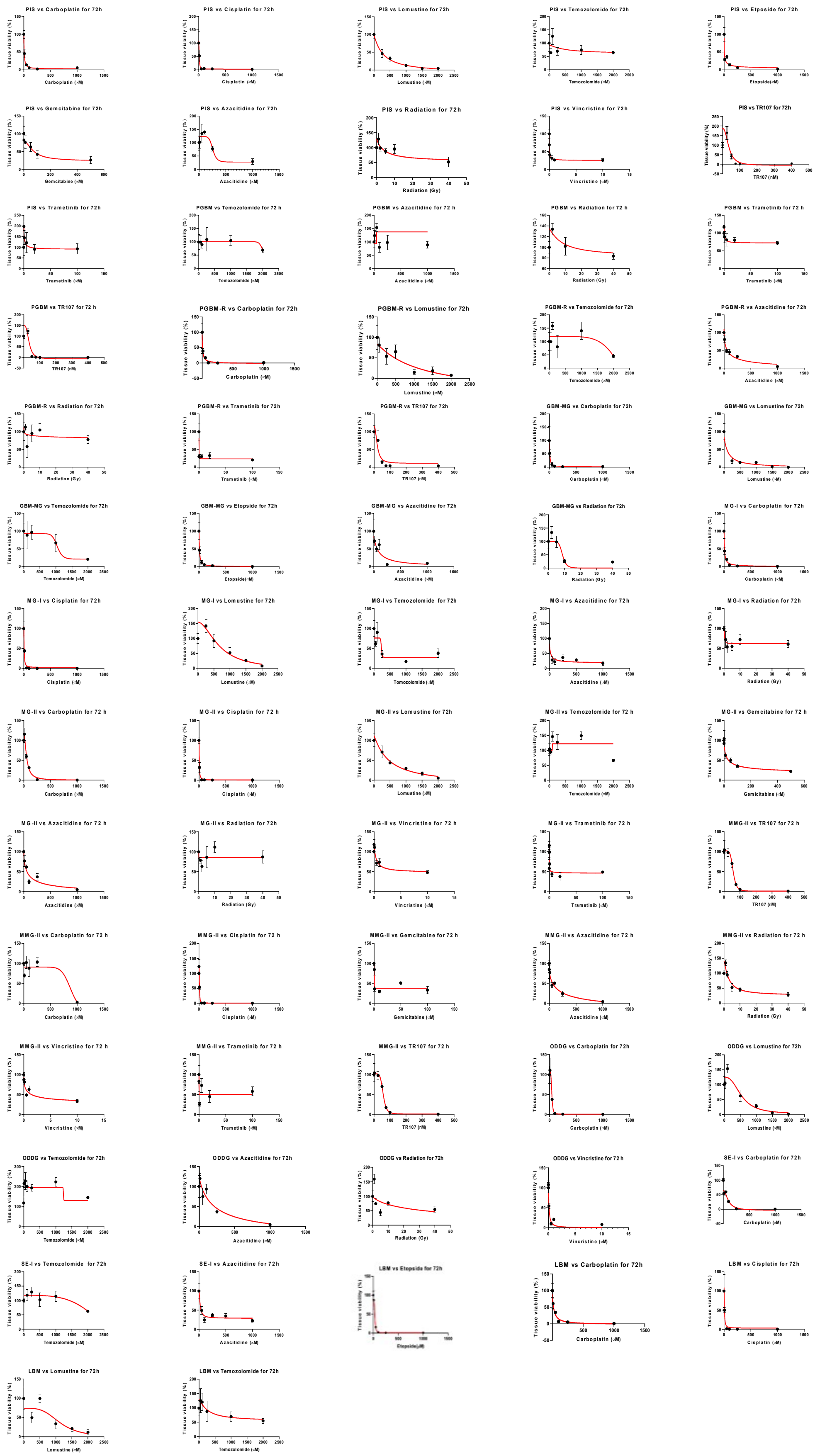

Figure S6, Related to Figure 7: Dose Response Curves for Patient Tissue Dose-response curves of each patient tumor against given therapeutic. To enable qualitative comparison, all datasets are fit with an inhibitor vs response variable slope (four parameters) least squares fit. This method poorly fits some curve types, such as those with increases in growth at low concentrations. Our IC50 and DSS calculations do not use these fitted curves, but instead use linear interpolation between calculated data points.

	**PIS**										
	Carboplatin	Azacitidine	Temozolomide	TR107	Vincristine	Gemcitabine	Trametinib	Cisplatin	Lomustine	Etoposide	Radiation
Killing at highest dose	9.47E-01	7.08E-01	3.58E-01	9.65E-01	7.41E-01	5.80E-01	6.58E-02	9.80E-01	9.55E-01	9.75E-01	4.75E-01
EC10	1.85E+00	2.19E+02	8.24E+01	3.99E-02	3.26E-03	2.78E-01	NR	2.09E+00	4.70E+01	1.40E+00	4.39E+00
EC25	4.62E+00	2.82E+02	2.06E+02	4.30E-02	8.16E-03	1.30E+01	NR	5.21E+00	1.17E+02	3.51E+00	1.77E+01
EC50	9.24E+00	6.74E+02	NR	4.81E-02	7.18E-02	8.10E+01	NR	1.17E+01	2.35E+02	7.02E+00	NR
EC75	3.62E+01	NR	NR	6.03E-02	NR	NR	NR	3.24E+01	6.78E+02	3.27E+01	NR
EC90	7.37E+01	NR	NR	7.03E-02	NR	NR	NR	4.49E+01	1.14E+03	1.74E+02	NR
Slope	-5.41E-02	-6.37E-04	-3.10E-05	-4.92E+01	-3.13E+00	-4.24E-03	-3.04E-03	-1.21E-02	-5.88E-04	-7.12E-02	-1.01E-02
AUC	5.93E+01	2.45E+02	1.47E+03	7.25E-02	2.71E+00	6.21E+01	9.67E+01	4.95E+01	4.56E+02	7.95E+01	1.47E+01
	**PGBM**										
	Carboplatin	Azacitidine	Temozolomide	TR107	Vincristine	Gemcitabine	Trametinib	Cisplatin	Lomustine	Etoposide	Radiation
Killing at highest dose		1.03E-01	3.07E-01	9.86E-01			2.80E-01				1.66E-01
EC10		9.32E+01	1.43E+03	3.22E-02			9.66E-01				2.93E+01
EC25		NR	1.84E+03	3.53E-02			7.00E+01				NR
EC50		NR	NR	4.06E-02			NR				NR
EC75		NR	NR	4.59E-02			NR				NR
EC90		NR	NR	4.90E-02			NR				NR
Slope		-1.08E-04	-3.60E-04	-4.74E+01			-1.00E-03				-6.12E-03
AUC		9.62E+02	1.92E+03	4.56E-02			7.73E+01				3.96E+01
	**PGBM-R**										
	Carboplatin	Azacitidine	Temozolomide	TR107	Vincristine	Gemcitabine	Trametinib	Cisplatin	Lomustine	Etoposide	Radiation
Killing at highest dose	9.91E-01	9.55E-01	5.33E-01	9.63E-01			7.92E-01		9.23E-01		2.23E-01
EC10	1.64E+00	5.21E+00	1.50E+02	1.04E-02			1.43E-02		5.43E+01		2.65E+01
EC25	4.10E+00	1.70E+01	5.22E+02	2.54E-02			3.58E-02		1.36E+02		NR
EC50	8.20E+00	4.73E+01	1.83E+03	3.57E-02			7.16E-02		3.26E+02		NR
EC75	3.56E+01	4.54E+02	NR	4.59E-02			7.22E+01		8.06E+02		NR
EC90	7.20E+01	8.54E+02	NR	6.12E-02			NR		1.68E+03		NR
Slope	-6.10E-02	-8.26E-03	-1.91E-04	-2.44E+01			-6.98E+00		-5.21E-04		-9.12E-03
AUC	2.72E+01	2.56E+02	2.06E+03	4.84E-02			2.77E+01		4.89E+02		3.66E+01
	**GBM-MG**										
	Carboplatin	Azacitidine	Temozolomide	TR107	Vincristine	Gemcitabine	Trametinib	Cisplatin	Lomustine	Etoposide	Radiation
Killing at highest dose	9.72E-01	9.01E-01	7.89E-01						9.96E-01	9.94E-01	7.70E-01
EC10	2.12E+00	3.64E+00	9.05E+01						3.04E+01	1.87E+00	1.37E+00
EC25	5.30E+00	9.11E+00	6.68E+02						7.61E+01	4.68E+00	6.64E+00
EC50	1.27E+01	1.33E+02	1.37E+03						1.52E+02	9.36E+00	8.39E+00
EC75	3.65E+01	2.00E+02	1.91E+03						2.28E+02	3.50E+01	2.48E+01
EC90	5.55E+01	2.40E+02	NR						1.15E+03	6.65E+01	NR
Slope	-1.05E-02	-3.73E-03	-4.58E-04						-3.29E-03	-5.34E-02	-1.43E-01
AUC	4.77E+01	1.73E+02	1.25E+03						-2.94E+00	4.37E+01	7.60E+00
	**MG-I**										
	Carboplatin	Azacitidine	Temozolomide	TR107	Vincristine	Gemcitabine	Trametinib	Cisplatin	Lomustine	Etoposide	Radiation
Killing at highest dose	9.88E-01	8.29E-01	6.20E-01					9.94E-01	9.19E-01		3.91E-01
EC10	1.79E+00	6.99E+00	1.02E+02					1.78E+00	5.27E+02		3.64E-01
EC25	4.49E+00	1.75E+01	1.43E+02					4.44E+00	7.17E+02		9.09E-01
EC50	8.97E+00	3.50E+01	2.12E+02					8.88E+00	1.05E+03		6.92E+01
EC75	4.31E+01	6.46E+02	NR					2.78E+01	1.55E+03		NR
EC90	8.41E+01	NR	NR					4.20E+01	1.95E+03		NR
Slope	-5.57E-02	-1.43E-02	-3.62E-03					-5.63E-02	-5.13E-04		-3.74E-03
AUC	4.42E+01	1.82E+02	6.50E+02					2.04E+01	1.24E+03		2.63E+01
	**MG-II**										
	Carboplatin	Azacitidine	Temozolomide	TR107	Vincristine	Gemcitabine	Trametinib	Cisplatin	Lomustine	Etoposide	Radiation
Killing at highest dose	9.95E-01	9.49E-01	3.46E-01	9.90E-01	5.22E-01	6.41E-01	5.11E-01	9.99E-01	9.44E-01		1.29E-01
EC10	2.82E+01	4.31E+00	3.53E+02	5.32E-02	3.12E-01	1.01E+00	2.39E-02	1.47E+00	8.58E+01		3.64E+00
EC25	3.90E+01	1.47E+01	1.36E+03	5.75E-02	9.64E-01	5.89E+00	5.98E-02	3.67E+00	2.15E+02		NR
EC50	6.70E+01	1.43E+02	NR	6.48E-02	9.21E+00	5.01E+01	2.85E+00	7.33E+00	4.39E+02		NR
EC75	1.31E+02	5.29E+02	NR	7.20E-02	NR	NR	NR	1.87E+01	1.19E+03		NR
EC90	2.06E+02	8.84E+02	NR	8.44E-02	NR	NR	NR	3.80E+01	1.81E+03		NR
Slope	-5.68E-03	-1.24E-03	-1.49E-04	-3.45E+01	-2.86E-02	-3.08E-03	-6.41E-02	-6.82E-02	-1.10E-03		-3.23E-03
AUC	1.03E+02	2.61E+02	2.69E+03	6.75E-02	1.93E+00	5.24E+01	4.37E+01	1.70E+01	7.14E+02		3.87E+01
	**MMG-II**										
	Carboplatin	Azacitidine	Temozolomide	TR107	Vincristine	Gemcitabine	Trametinib	Cisplatin	Lomustine	Etoposide	Radiation
Killing at highest dose	9.73E-01	9.58E-01		9.93E-01	6.61E-01	6.69E-01	5.74E-01	9.98E-01			7.20E-01
EC10	9.31E+01	4.40E+00		3.21E-02	7.86E-03	3.24E-01	4.73E-02	2.14E+00			2.33E+00
EC25	2.37E+02	1.28E+01		4.76E-02	1.78E-01	5.99E-01	2.07E+00	5.34E+00			3.39E+00
EC50	5.01E+02	4.40E+01		5.95E-02	4.84E-01	8.59E-01	1.58E+01	1.24E+01			7.03E+00
EC75	7.65E+02	2.42E+02		7.14E-02	NR	NR	NR	3.14E+01			NR
EC90	9.23E+02	7.81E+02		9.01E-02	NR	NR	NR	4.28E+01			NR
Slope	-9.49E-04	-8.02E-03		-2.10E+01	-8.17E-01	-9.63E-01	-1.78E-02	-1.32E-02			-1.14E-02
AUC	6.31E+02	2.19E+02		7.04E-02	4.98E+00	4.08E+01	4.98E+01	2.10E+01			1.82E+01
	**ODDG**										
	Carboplatin	Azacitidine	Temozolomide	TR107	Vincristine	Gemcitabine	Trametinib	Cisplatin	Lomustine	Etoposide	Radiation
Killing at highest dose	9.95E-01	9.61E-01	-4.46E-01		9.17E-01				9.86E-01		4.53E-01
EC10	2.15E+01	3.66E+01	NR		2.20E-02				1.33E+02		7.82E-01
EC25	2.98E+01	4.99E+01	NR		6.54E-02				3.33E+02		1.96E+00
EC50	4.35E+01	1.81E+02	NR		2.18E-01				6.81E+02		NR
EC75	6.83E+01	5.22E+02	NR		6.41E+00				1.07E+03		NR
EC90	8.94E+01	8.61E+02	NR		8.74E+00				1.40E+03		NR
Slope	-1.82E-02	-1.89E-03	2.23E-04		-3.76E-01				-6.90E-04		-7.52E-03
AUC	5.85E+01	3.43E+02	3.73E+03		1.24E-01				9.66E+02		9.09E+00
	**SE-I**										
	Carboplatin	Azacitidine	Temozolomide	TR107	Vincristine	Gemcitabine	Trametinib	Cisplatin	Lomustine	Etoposide	Radiation
Killing at highest dose	9.85E-01	8.04E-01	3.44E-01								
EC10	2.16E+00	2.04E+00	1.50E+03								
EC25	5.39E+00	5.11E+00	1.81E+03								
EC50	6.41E+01	1.69E+01	NR								
EC75	NR	7.23E+02	NR								
EC90	NR	NR	NR								
Slope	-6.39E-03	-1.50E-03	-4.93E-04								
AUC	7.32E+01	2.95E+02	2.00E+03								
	**LBM**										
	Carboplatin	Azacitidine	Temozolomide	TR107	Vincristine	Gemcitabine	Trametinib	Cisplatin	Lomustine	Etoposide	Radiation
Killing at highest dose	9.91E-01		4.48E-01					9.95E-01	8.79E-01	9.90E-01	
EC10	2.57E+00		2.42E+02					1.99E+00	4.98E+01	7.83E+00	
EC25	6.41E+00		5.19E+01					4.98E+00	1.25E+02	1.68E+01	
EC50	2.65E+01		NR					9.97E+00	2.49E+02	3.08E+01	
EC75	6.72E+01		NR					1.52E+01	1.35E+03	4.48E+01	
EC90	9.47E+01		NR					1.83E+01	NR	7.10E+01	
Slope	-6.66E-03		-1.50E-04					-5.02E-02	-2.01E-03	-1.79E-02	
AUC	6.97E+01		1.50E+03					1.46E+01	9.28E+02	4.31E+01	

Table S3, Related to Figure 7: Killing Parameters on OBSCs. Dose-response values used to generate DSS from each cell line against each therapeutic. Values for Killing at Highest Dose are given as fraction of tumor population killed. EC values are given as small molecule drug concentrations in μM or as XRad dose in Gy. NR indicates the IC50 was not reached within the dose range. Linear interpolation between calculated data points used to calculate individual EC values. Trapezoidal rule used to calculate AUC.

Tumor Name	Primary pathology diagnosis	Age	Mutations	Treatment
PGBM	GBM	Pediatric	IDH1/2 IDH1 c.395G>A mutation detected, TERT-, methylated MGMT, GFAP+, OLIG2+, IDH1132H+, H3K27M-, patchy expression of BRAF V600E, MS-Stable, IDH1 = R132H, PIK3CA = H1047R, CDK4 amplification, PAX5 = V129M, TP53 = R273C	Xrad, Veliparib, TMZ
MG-I	WHO I meningioma	Adult	Synaptophysin+, GATA3+, growth hormone-, ACTH-, prolactin-	No Further Treatment
GBM-MG	GBM w/ WHO I meningioma	Adult	GBM: GFAP+, rare positivity for OLIG2, IDH1-, ATRX-, p53-, p16-, Ki-67 5-10%. WHO I meningioma: GFAP-,	TMZ (treatment delayed)
LBM	Stage III NSCLC (adenocarcinoma)	Adult	Mock 31+, CK7+, rare scattered cells positive for TTF-1 and CDX-2, Napsin-A-, GATA 3-, CK20-, p40-, PAX 8-,	Xrad; GammaTiles internal radiation
PIS	Intracranial sarcoma	Pediatric	DICER1-mutant+	Xrad, etoposide, ICE
SE-I	WHO I subependymoma	Adult	GFAP+, EMA+, Ki-67 <1%	No Further Treatment
PGBM-R	GBM (Recurrent tumor from same patient as PGBM)	Pediatric	MS-stable, CDK4 amplification, IDH1 R132H, PAX5 V129M, PIK3CA H1047R, TP53 R273C	TMZ; REMIND Trial: associated antigen T-cell infusion
MG-II	WHO grade II meningioma	Adult	EMA+ with subset of cells, GFAP+, Ki-67+ with proliferation index at 15-20 percent	Xrad
MMG-II	WHO grade II meningioma (multiple tumors)	Adult	Ki-67+ with proliferation index at 5-10 percent	Xrad
ODDG	WHO grade III oligodendroglioma	Adult	GFAP+, OLGI2+, IDH1 R132H+, MGMT+, ATRX+, P16-, P53-, Positive for TERT promoter -146C>T (C250T) mutation, Abnormal FISH Result Consistent with Codeletion of 1p and 19q	TMZ + Xrad
GG-I	WHO grade I ganglioglioma	Adult	GFAP+, no immunohistochemical evidence of alterations in IDH1, BRAF, or H3 K27M, and low Ki-67 proliferation index	No Further Treatment

Table S4, Related to Figure 7: Patient Tumor Clinical Data. Data was derived from clinical records in accord with our IRB-approved protocol.

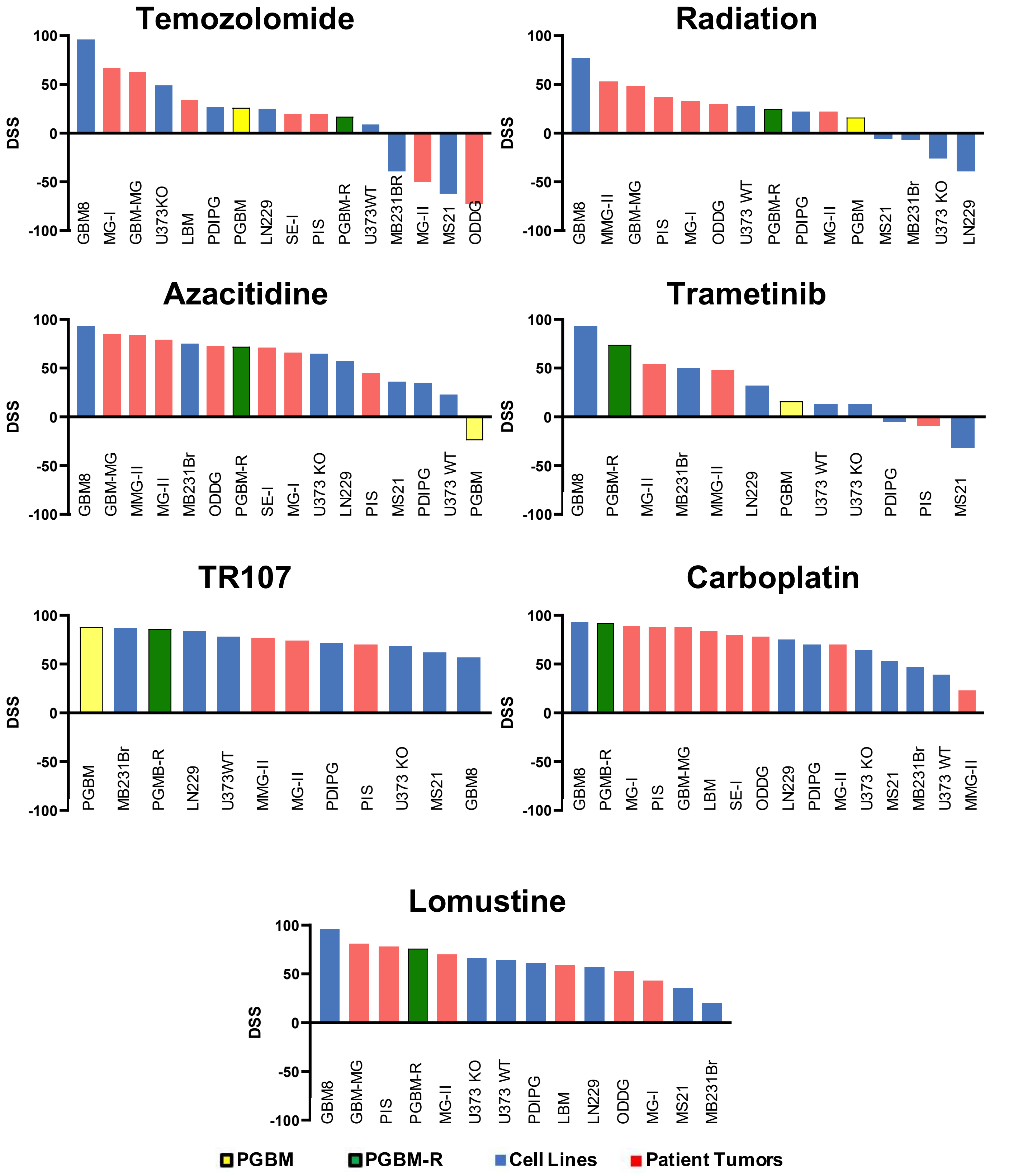

Figure S7, Related to Figure 7: Waterfall Plots with All Tumor IDs. Waterfall plots depicting relative sensitivities to individual therapeutics. All tumors treated with each therapeutic are included and identified on x-axis. Yellow lines with black outline = DSS for PGBM; green lines with black outline = DSS for PGBM-R; blue lines = DSS for established tumor lines; red lines = DSS for patient tumor tissue.
